# Deep learning based approach for actinidia flower detection and gender assessment

**DOI:** 10.1038/s41598-024-73035-1

**Published:** 2024-10-18

**Authors:** Isabel Pinheiro, Germano Moreira, Sandro Magalhães, António Valente, Mário Cunha, Filipe Neves dos Santos

**Affiliations:** 1 Institute for Systems and Computer Engineering, Technology and Science (INESC TEC), Porto, 4200-465 Portugal; 2 School of Science and Technology, University of Trás-os-Montes e Alto Douro, Vila Real, 5000-801 Portugal; 3 Faculty of Sciences, University of Porto, Porto, 4169-007 Portugal; 4 Faculty of Engineering, University of Porto, Porto, 4200-465 Portugal

**Keywords:** Computer vision, Kiwifruit, Object detection, Precision agriculture, Environmental sciences, Engineering

## Abstract

Pollination is critical for crop development, especially those essential for subsistence. This study addresses the pollination challenges faced by Actinidia, a dioecious plant characterized by female and male flowers on separate plants. Despite the high protein content of pollen, the absence of nectar in kiwifruit flowers poses difficulties in attracting pollinators. Consequently, there is a growing interest in using artificial intelligence and robotic solutions to enable pollination even in unfavourable conditions. These robotic solutions must be able to accurately detect flowers and discern their genders for precise pollination operations. Specifically, upon identifying female Actinidia flowers, the robotic system should approach the stigma to release pollen, while male Actinidia flowers should target the anthers to collect pollen. We identified two primary research gaps: (1) the lack of gender-based flower detection methods and (2) the underutilisation of contemporary deep learning models in this domain. To address these gaps, we evaluated the performance of four pretrained models (YOLOv8, YOLOv5, RT-DETR and DETR) in detecting and determining the gender of Actinidia flowers. We outlined a comprehensive methodology and developed a dataset of manually annotated flowers categorized into two classes based on gender. Our evaluation utilised k-fold cross-validation to rigorously test model performance across diverse subsets of the dataset, addressing the limitations of conventional data splitting methods. DETR provided the most balanced overall performance, achieving precision, recall, F1 score and mAP of 89%, 97%, 93% and 94%, respectively, highlighting its robustness in managing complex detection tasks under varying conditions. These findings underscore the potential of deep learning models for effective gender-specific detection of Actinidia flowers, paving the way for advanced robotic pollination systems.

## Introduction

Pollination is a fundamental biological process essential for the development and yield of many crops. This process involves the transfer of pollen from the male anthers to the female stigma. Nevertheless, the specific characteristics of each plant species can introduce complexities to pollination^1^. In most flowering plants, hermaphroditism is prevalent, with individual flowers containing both male and female reproductive organs. However, a minority of species exhibit dioecy, with male and female flowers on distinct plants. For successful pollination across different individuals, it is crucial to synchronise the flowering periods of male and female plants^2^.

In Actinidia, a dioecious plant, successful pollination requires the transfer of pollen from the male anthers to the female stigma. The female flowers exhibit both female (multi-carpellary stigma) and male (anthers) reproductive organs. However, the pollen found in the anthers of female flowers is not viable. In contrast, male flowers only possess male reproductive organs (anthers) containing substantial amounts of viable pollen grains^3^. These viable pollen grains from the male anthers can fertilize the ovules of the female flowers, leading to the formation of seeds. A higher number of seeds triggers hormone production and leads to larger fruits with a more uniform shape^3–5^, which impacts quality and yield. This critical phase of the vegetative cycle is typically carried out by wind (anemophilous pollination) or insects (entomophilous pollination).

Insects are the most extensive group of pollinators, with bees leading the way in pollinating approximately 71 of the 100 crops that produce 90% of the world’s food^6^. Bees are recognised as the best pollinators due to the large size of their colonies and their remarkable floral constancy. These characteristics increase the efficiency and effectiveness of pollen transfer between flowers of the same species^7^. Plants can produce two primary resources of interest to bees: pollen and nectar. Even though pollen is important for developing larvae, bees are mainly attracted to flowers by their nutritious nectar.

Actinidia flowers contain only protein-rich pollen, so pollinators do not tend to seek out these flowers. Therefore, Actinidia flowers are not very attractive to insects, especially when there is a diversity of nearby flora, leading to deficiencies in the pollination process and, consequently, in the yield and quality of the fruit^3,5^.

Natural pollination (anemophilous and entomophilous) is considered inefficient in commercial kiwi orchards, prompting growers to seek solutions to improve or complement natural pollination. Hence, kiwi producers often use assisted pollination techniques to achieve a higher success rate of fertilised flowers, better uniformity of fruit shape, and more consistent production. Assisted pollination can occur through the manual or mechanical application of pollen, using dry or wet methods, with pollen that has been previously collected and preserved^4^.

The principal assisted pollination techniques utilised are economically inefficient, pose environmental risks, and contribute to biodiversity loss^8^. In this way, there has been increasing interest in robotic solutions. These engineering-based solutions can potentially increase production regularisation, enhance crop quality, and decrease the environmental impact of agriculture by reducing the reliance on natural pollinators and increasing pollination efficiency^9^.

A crucial aspect of these robotic solutions for precise pollination is the perception system, particularly in accurately detecting the flower and assessing its gender. This capability enables the robotic solution to execute precise actions according to the flower’s gender: (1) female flower detection must trigger the robotic solution to approach the stigma and release pollen; (2) male flower detection must trigger the robotic solution to approach the anthers and collect the pollen.

Computer Vision (CV) is a highly effective and widely used technology for robotic solutions in agriculture, capable of extracting valuable information and constructing explicit and meaningful descriptions of physical objects from images or videos. In recent years, the development of computer vision and perception algorithms has been significantly influenced by the strong learning capabilities of deep learning (DL)^10,11^. Convolutional Neural Networks (CNNs) are the primary DL algorithms used for computer vision tasks and are extensively utilised in agriculture^12^. CNNs can analyse, combine and extract features from images, demonstrating high effectiveness and efficiency in classification, localisation and object detection tasks. The literature presents multiple CNN-based DL models, such as Faster Region-based Convolutional Neural Networks (R-CNN)^13^, Single Shot Multibox Detector (SSD)^14^, and You Only Look Once (YOLO)^15^, among others.

The accessibility and visibility of Actinidia flowers are two significant challenges that CV systems face. Problems such as varying light intensity and the overlapping and occlusion of identified flowers make it difficult to achieve the desired goal. Unlike traditional methods, DL techniques have been the most robust and accurate alternative, offering results with superior metrics in the above-mentioned challenges (occlusion and overlap).

Recent studies have focused on enhancing pollination efficiency by improving the recognition of Actinidia flowers and stamens. One such investigation employed a modified YOLOv5s model to address flower overlap and enhance feature extraction accuracy, ultimately reducing detection errors. The study involved optimizing the model to improve overlapping flower recognition, calculating tilt angles more precisely, and accurately identifying flower coordinates. These improvements were achieved by augmenting the original dataset from 880 quality images to 3344 images. The modified YOLOv5s model was then compared with Faster R-CNN ResNet50, Faster R-CNN VGG, SSD VGG, and SDD MobiliNet v2, with YOLOv5s achieving a 97% accuracy, the best value among the models analysed^16^.

Further research has explored the real-time detection of Actinidia flowers and buds using YOLOv3 and YOLOv4 models for robotic pollination. The study initially used a dataset of 830 images of *Actinidia chinensis* cv. ’Hayward’, later expanded to 3790 images after augmentation. The YOLOv4 model outperformed YOLOv3, achieving a 97% of AP for the flower class^17^.

Another study investigated the detection and segmentation of Actinidia flowers using the SOLOv2 model. Initially based on 267 images of *Actinidia chinensis* cv. ’Hayward’, the dataset was expanded to 1210 images after augmentation. The SOLOv2 model’s performance was compared with YOLOv5, Mask R-CNN, SOLO, and YOLACT models, with YOLOv5 obtaining the best metrics in flower segmentation with 67% accuracy^18^.

In a pioneering approach, Deep Neural Networks (DNN) have been integrated into an autonomous robotic system for Actinidia flower detection and pollination. This study aimed to explore DNN for the detection of Actinidia flowers by investigating two state-of-the-art object detectors, Faster R-CNN and SSD Net, and feature extractors, Inception Net V2 and Neural Architecture Search (NAS) Net, with real-world orchard datasets. The dataset used has 1,451 manually annotated images. The Faster R-CNN Inception V2 model has the best overall metric values with 97% accuracy, 68% recovery, and 79% F1 score^19^.

Another study evaluated the effectiveness of robotic pollination in kiwifruit production by focusing on Actinidia flower detection using deep learning methods. The dataset used has 1451 images, and flower annotation was performed manually, with the stigma area on all flowers annotated, including all flowers obscured or obstructed by the canopy. The Faster R-CNN Inception v2 model achieved 91% precision, 80% recall, and 85% F1 score^20^.

Finally, research into multiclass detection of Actinidia flowers and their spatial distribution in orchards has highlighted the importance of accounting for asynchronous flowering times. The study aimed to identify the phenological stages of flowers to optimize pollination strategies. The state of development of each flower (phenological stage) plays a crucial role in pollination. Identifying the right flowers to pollinate based on phenology and flower distribution is essential for optimising production. For this purpose, 355 images were acquired (manually annotated), and by performing augmentation operations, 1704 images were obtained. The YOLOv5l model grouped nine classes of flowers and achieved a 93% AP^21^.

The article identifies two research gaps: (1) the lack of flower detection according to gender and (2) the absence of recent DL models in this context. This study aims to address these gaps through the following main contributions:

- A comprehensive review of the related work in object detection of Actinidia flowers, with a focus on gender detection;
- The generation of the *Actinidia chinensis* cv. ’Hayward’ Flower Dataset 2024^22^, a novel and publicly available dataset comprising 556 images with a resolution of $3000 \times 3000$, annotated by flower gender in both Pascal VOC and YOLO formats;
- The presentation of *Actinidia chinensis* cv. ’Hayward’ Flower Dataset 2024 (augmented version)^23^, which includes the 556 original images and 3332 augmented images with the corresponding annotations (Pascal VOC and YOLO format) by flower gender;
- Performance analysis of four recent DL models for detecting Actinidia flowers and assessing their gender.

Implementing this innovative approach enables the development of a robotic pollination system to identify male Actinidia flowers for pollen collection and then locate female Actinidia flowers for pollination. This method ensures precise and efficient pollination, increasing homogeneity and productivity in Actinidia cultivation.

## Materials and methods

**Fig. 1:**
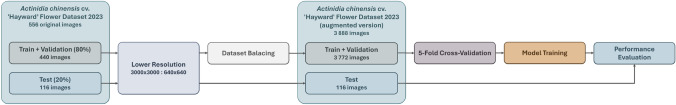
Workflow of the research methodology with distinct dataset creation, model training and performance evaluation phases for *Actinidia chinensis* cv. ’Hayward’ flower detection and gender determination.

This section outlines the methodology used in this research, covering the entire process from data collection to model training and performance evaluation. Figure 1 gives an overview of the process flow used in the study, which is detailed step by step below. (An enlarged version can be found as Suplementary Fig. S1.)

Figure 1 detailed workflow of the research methodology for *Actinidia chinensis* cv. ’Hayward’ flower detection and gender determination. The *Actinidia chinensis* cv. ‘Hayward’ Flower Dataset 2024 consists of 556 original images, which were split into 80% for training and validation (440 images) and 20% for testing (116 images). After reducing the resolution, the number of images in the training + validation set was increased to create a well-balanced dataset of 3888 images. The *Actinidia chinensis* cv. ‘Hayward’ Flower Dataset 2024 (augmented version) was used for 5-fold cross-validation to ensure robust model training. Finally, the performance of the trained model was evaluated using the test set to ensure accurate flower detection and gender determination.

### Data collection

The initial step in creating the original dataset for *Actinidia chinensis* cv. ’Hayward’ Flower Dataset 2024 involved systematically collecting data to meet the research objectives. During this study, the data acquisition phase was conducted at Quinta do Carrazedo (41.32170534304873, $-8.661668130688255$), utilizing a single camera of iPhone Xr to capture images at a resolution of $3000 \times 3000$ pixels.

A comprehensive assortment of images was deliberately amassed under varying lighting conditions and perspectives to form a rich dataset with ample visual information. The collected imagery encompasses both individual flowers and floral arrangements, introducing complexity that enables the evaluation of model performance in occluded and overlapping flowers, as influenced by diverse plant structures, including leaves, stems, trunks, and neighbouring flowers. This image compilation culminated in a total of 556 original images.

**Fig. 2:**
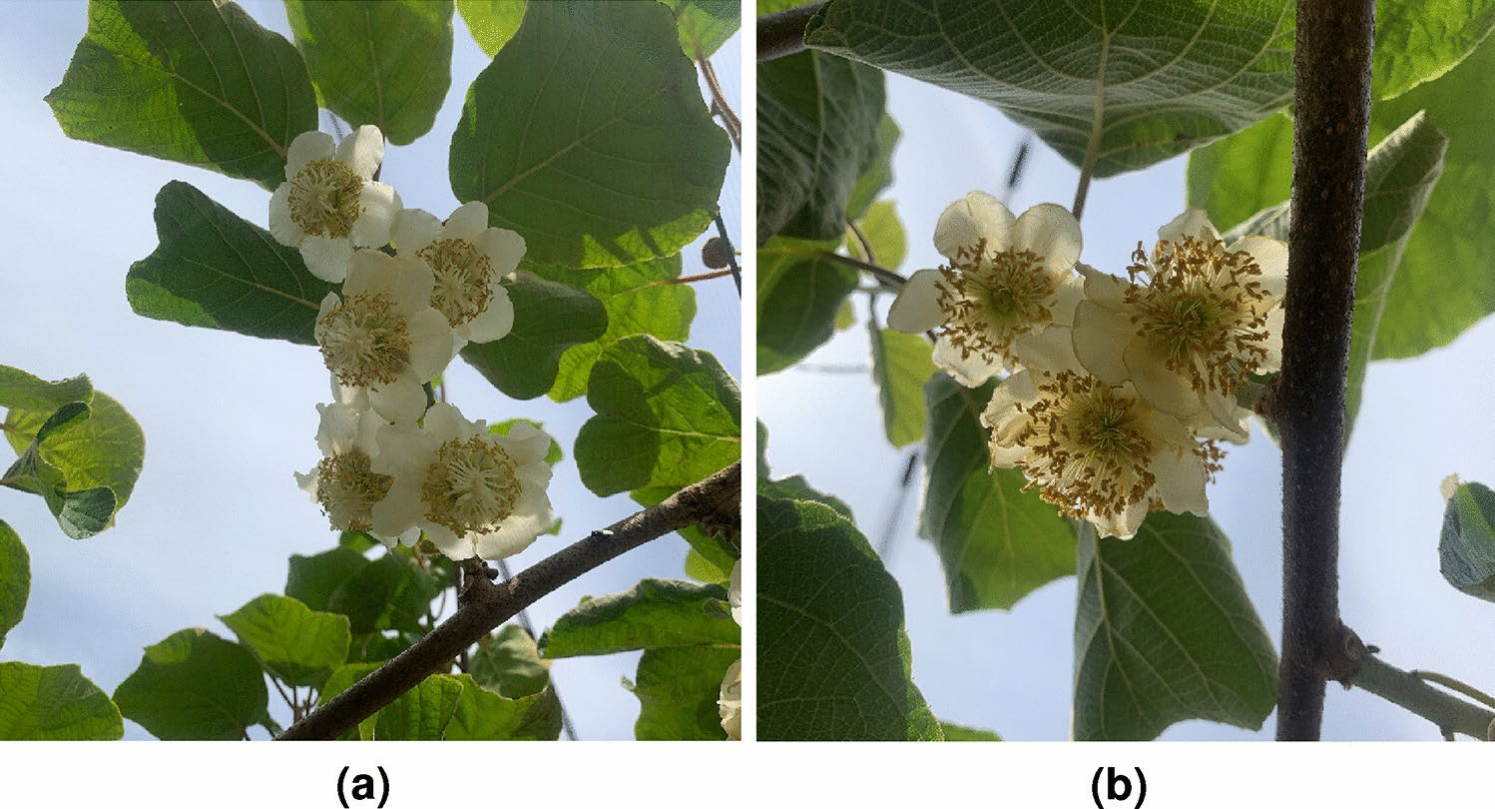
Examples of images from the *Actinidia chinensis* cv. ’Hayward’ Flower Dataset 2024. (**a**) Female Actinidia flowers. (**b**) Male Actinidia flowers..

This study aimed to detect Actinidia flowers and determine their gender. The dataset collected contains detailed information on various flowers under different conditions. Figure 2 illustrates examples of Actinidia female and Actinidia male flowers from the dataset.

### Data generation

After acquiring the images, each object underwent manual annotation using the Computer Vision Annotation Tool (CVAT). Every annotation included specific details of a bounding box, delineating the object and providing information about its area, position, and class.

**Fig. 3:**
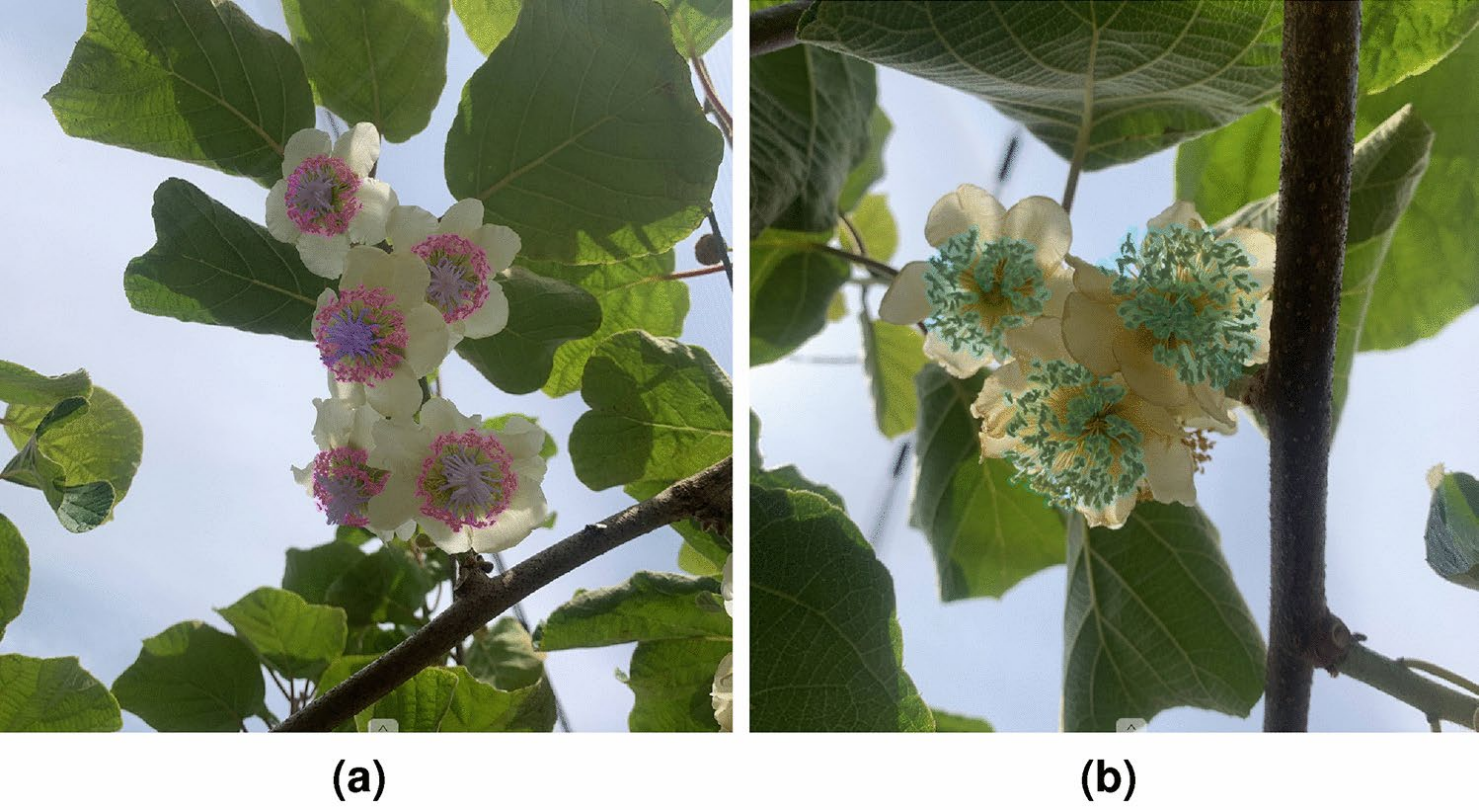
Key morphological characteristics of *Actinidia chinensis* cv. ’Hayward’ flowers according to gender. (**a**) Female Actinidia flowers. The purple shadow indicates the star-shaped stigma. The pink shadow indicates the anthers with non-viable pollen. (**b**) Male Actinidia flowers. The blue shadow indicates well-developed anthers..

The process of annotating Actinidia flowers according to gender required careful consideration of key morphological characteristics. Female flowers are distinguished by their prominent, star-shaped stigma. Although these flowers may have anthers, they usually contain minimal amounts of sterile, non-viable pollen^3^. Figure 3a shows the star-shaped stigma shaded in purple and the anthers with non-viable pollen shaded in pink. On the other hand, the male flowers only have numerous, well-developed anthers containing abundant viable pollen grains^3^. Figure 3b shows the well-developed anthers typical of the male flower shaded in blue.

**Fig. 4:**
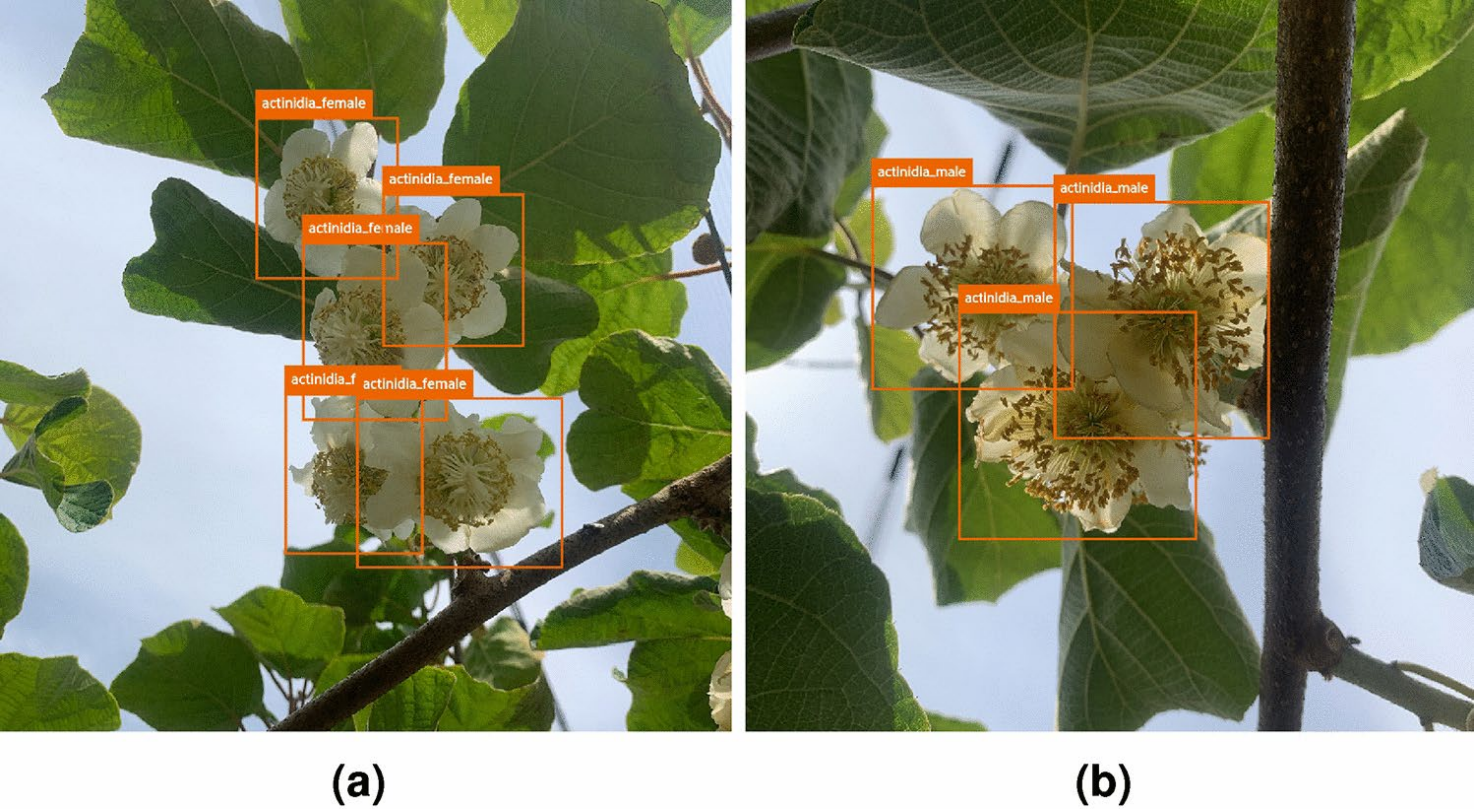
Examples of annotations from the *Actinidia chinensis* cv. ’Hayward’ Flower Dataset 2024. (**a**) Female Actinidia flowers annotation. (**b**) Male Actinidia flower annotation..

The *Actinidia chinensis* cv. ’Hayward’ Flower Dataset 2024^22^ was carefully annotated to include two distinct classes related to gender: actinidia_female and actinidia_male. Figure 4 illustrates images from the dataset with manual annotations represented by orange bounding boxes for each class. The images were exported from CVAT under the Pascal Visual Object Classes (VOC) format, a commonly known format related to the Pascal VOC Challenge, where each image file has a linked Extensible Markup Language (XML) file with annotations.

The distribution of images and annotations across individual classes and the aggregated totals within the *Actinidia chinensis* cv. ‘Hayward’ Flower Dataset 2024 is presented in Table 1. Some images include objects from both classes, while others lack discernible objects (such as leaves, stems, trunks, or flower components). The disparity in object counts across classes is due to the planting environment at Quinta do Carrazedo, where a ratio of one male Actinidia plant to six female Actinidia plants is maintained to facilitate pollination. Consequently, a higher representation of female flowers in the images is expected.

**Table 1:** Number of images and annotations for each class in the *Actinidia chinensis* cv. ’Hayward’ Flower Dataset 2024.

	Actinidia_female	Actinidia_male	Total
Images	488	68	556
Annotations	2347	289	2636

**Fig. 5:**
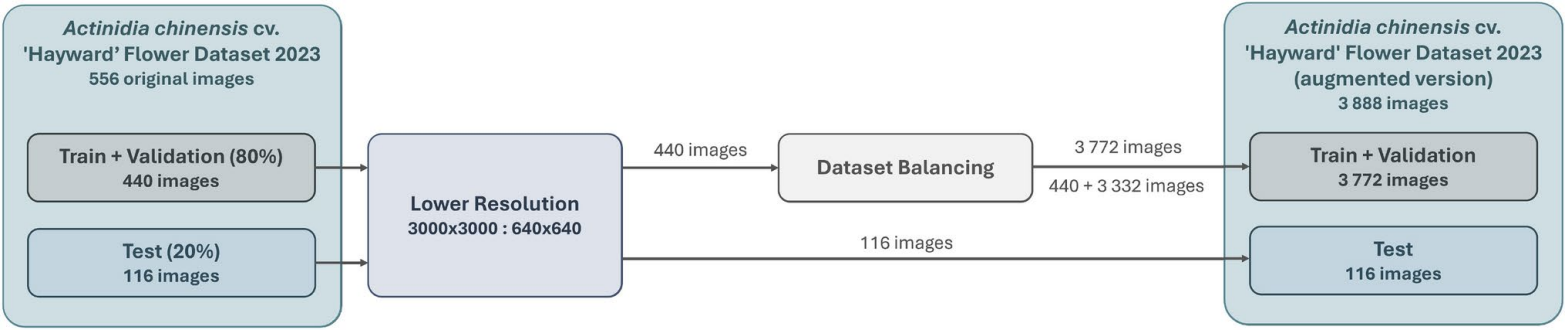
Workflow to reach the *Actinidia chinensis* cv. ’Hayward’ Flower Dataset 2024 (augmented version) with two distinct lower resolution and dataset balancing phases.

The *Actinidia chinensis* cv. ‘Hayward’ Flower Dataset 2024 underwent different operations to optimize data for the DL models’ performance. Figure 5 outlines the different processes to acquire the *Actinidia chinensis* cv. ’Hayward’ Flower Dataset 2024 (augmented version).

The original resolution of $3000 \times 3000$ pixels resulted in a substantial data volume for neural network analysis, increasing complexity and processing time. To address this, the resolution was reduced to $640 \times 640$ pixels, thereby decreasing complexity while preserving the aspect ratio. However, this reduction also led to a loss of image detail.

Splitting the dataset into training, validation, and test sets is crucial to maximize the robustness and generalizability of deep learning models and avoid over-reliance on memorization of the training data. The training set serves as the foundation for model learning, while the validation set facilitates hyperparameter tuning and prevents overfitting during the training process. Finally, an independent test set objectively assesses the model’s performance on previously unseen data, demonstrating its real-world relevance.

While the *Actinidia chinensis* cv. ‘Hayward’ Flower Dataset 2024 was not used for training the neural networks, it was imperative to split the dataset into a training + validation set and a test set in this phase. This separation is crucial because all augmentation processes must be applied exclusively to the training + validation set. The Actinidia chinensis cv. ’Hayward’ Flower Dataset 2024 was divided into two sets, with 80% dedicated to the train + validation set and 20% allocated to the test set. The image allocation aimed to ensure an equitable distribution of both classes across the two sets. Table 2 indicates the number of images and annotated objects per class within each specified set. In this table, the number indicated for the total number of images in the dataset may be less than the sum of the number of images in each class since both classes may appear in one image.

**Table 2:** Number of images and annotated objects per class in each set of *Actinidia chinensis* cv. ’Hayward’ Flower Dataset 2024.

Classes	Train + validation		Test	
	Images	Annotation	Images	Annotations
Actinidia_female	386	1897	102	450
Actinidia_male	54	219	14	70
Total	440	2116	116	520

**Fig. 6:**
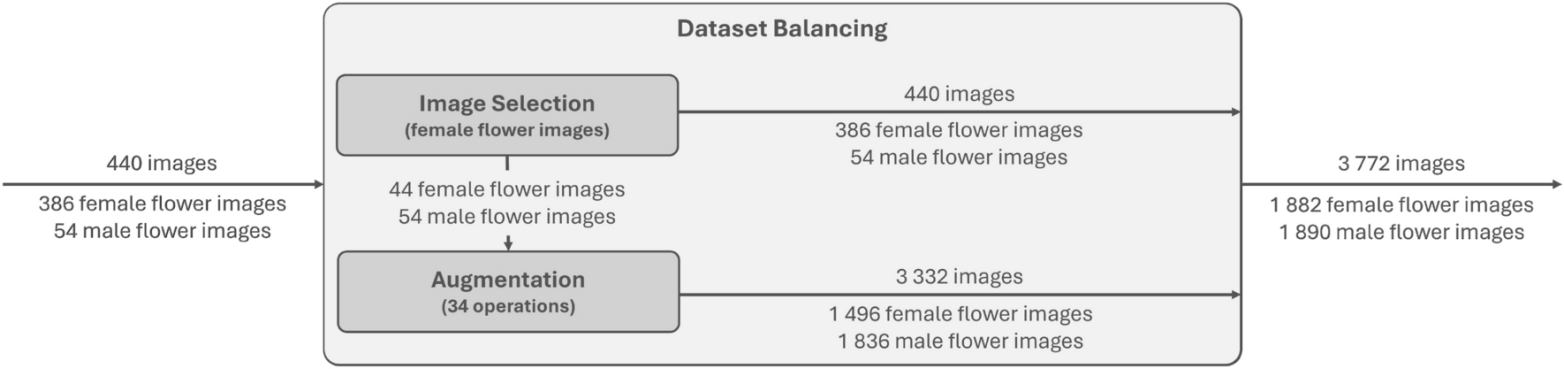
Workflow to reach dataset balancing with two distinct image selection and augmentation phases.

Ensuring optimal performance of the DL models required addressing the dataset imbalance, a critical step due to the significant difference between the 386 images with actinidia_female annotations and the 54 images with actinidia_male annotations. This disparity posed a potential bias towards the majority class, jeopardizing the model’s ability to accurately classify and generalize both classes. Rectifying the dataset imbalance aims to mitigate bias, enhance accuracy and recovery for the minority class, and fortify the overall robustness and generalizability of the model. As DL training models rely heavily on extensive data to effectively process new information, it was decided to use augmentation operations in order to equalise the dataset. Therefore, failure to execute this step may compromise the accuracy and precision of the models. As a result, a more reliable and equitable performance within real-world applications is anticipated. Figure 6 presents the methods utilised to achieve class balance in detail.

Before applying thirty-four augmentation operations, images were meticulously selected from the train + validation set to create a balanced dataset with the largest possible number of images. All 54 images of male flowers were included in the augmentation process, while a carefully chosen subset of 44 images from the 386 available female flower images was used. This selection ensured that the final dataset contained approximately equal numbers of male and female flower images, with 1882 female flower images and 1890 male flower images.

**Fig. 7:**
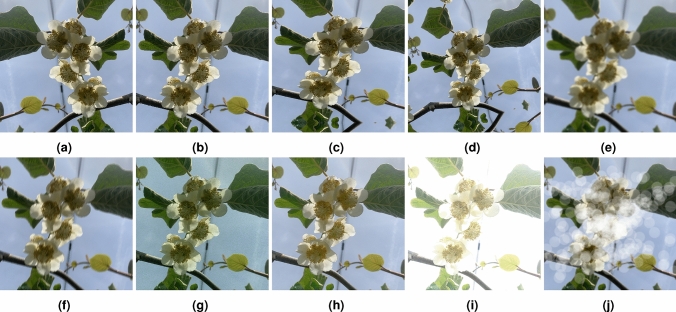
Augmentation operations applied to *Actinidia chinensis* cv. ’Hayward’ Flower Dataset 2024. (**a**) Flip. (**b**) Downscale. (**c**) GridDistortion. (**d**) SafeRotation. (**e**) GaussianBlur. (**f**) MotionBlur. (**g**) PixelDropout. (**h**) ISONoise. (**i**) RandomBrightnessContrast. (**j**) RandomFog.

The augmentation operations were performed with the resource of Albumentations^24^, a Python library designed for image augmentations. The operations were carefully selected, and only those generating realistic images were applied. Table 3 describes the specified operations performed on the *Actinidia chinensis* cv. ’Hayward’ Flower Dataset 2024 and Fig. 7 presents an example of each augmentation operation.

**Table 3:** Description of the augmentation operations selected to increase the *Actinidia chinensis* cv. ’Hayward’ Flower Dataset 2024.

Operation	Description
Flip	Mirror the image horizontally, vertically, or both horizontally and vertically
Downscale	Decrease the image quality by downscaling and upscaling back
GridDistortion	Grid distortion and elasticity transform the image
SafeRotation	Rotate the image by an angle selected randomly from the uniform distribution
GaussianBlur	Blur the image using a Gaussian filter with a random kernel size
MotionBlur	Apply motion blur to the input image using a random sized kernel
PixelDropout	Set pixels to 0 with some probability
ISONoise	Apply camera sensor noise
RandomBrightnessContrast	Randomly changes the brightness and contrast of the image
RandomFog	Simulates fog for the image

The combination of these operations can generate compelling images that enhance the dataset. The first four operations (Flip, Downscale, GridDistortion, and SafeRotation) involve displacing or removing pixels without altering their values. The remaining six operations (GaussianBlur, MotionBlur, PixelDropout, ISONoise, RandomBrightnessContrast, and RandomFog) modify the pixel values. For this reason, was decided to combine the operations based on these two groups, as detailed by Buslaev et al.^24^. This integration resulted in 34 operations, which were applied to a set of 98 selected images, resulting in 3332 augmented images.

The *Actinidia chinensis* cv. ‘Hayward’ Flower Dataset 2024 (augmented version)^23^ merged these 3332 augmented images with the original 440 images, resulting in a comprehensive dataset of 3772 images. Table 4 indicates the number of images and annotated objects per class within each specified set.

**Table 4:** *Actinidia chinensis* cv. ’Hayward’ Flower Dataset 2024 (augmented version) division into two sets, with the number of images and annotated objects per class in each set.

Classes	Train + validation		Test	
	Images	Annotation	Images	Annotations
Actinidia_female	1919	9024	102	450
Actinidia_male	1859	7356	14	70
Total	3772	16,380	116	520

### Model configuration and training

The DL models selected for benchmarking include YOLOv5, YOLOv8, RT-DETR, and DETR. These models were specifically chosen for their ability to achieve an optimal balance between accuracy and speed, aligning with the proposed objective of enabling precise, real-time flower detection and gender assessment^15^.

The YOLOv5 is a single-stage object detection algorithm that predicts bounding box localization and classification probability from the input image. This algorithm is recognized for its exceptional accuracy in detecting objects and fast inference speed, making it well-suited for real-time applications. As an evolution of the YOLO family, it has been optimized to perform effectively on large-scale datasets. Key attributes of YOLOv5 include the anchor-free split Ultralytics head, which revolutionizes object detection by removing the need for predefined anchor boxes, thereby improving performance across various scenarios. The architecture of YOLOv5 integrates CSPDarknet53 as the backbone for feature extraction, PANet as the neck for feature aggregation, and a custom head for prediction, ensuring a balance between speed and accuracy^25^.

The release of YOLOv8 signifies a substantial progression within the YOLO series, encompassing advancements over its predecessors. These enhancements involve improved handling of small objects and augmented generalization capabilities for real-time object detection tasks. In contrast to YOLOv5, YOLOv8 incorporates advanced backbone and neck architectures that further bolster feature extraction and overall detection performance. Moreover, it continues to utilize the anchor-free split Ultralytics head to enhance accuracy and efficiency in object detection, distinguishing it from earlier YOLO iterations. Specifically designed to achieve a harmonious balance between accuracy and speed, YOLOv8 is well-suited for demanding real-time applications in diverse environments^26^.

The DEtection TRansformer (DETR) marks a significant breakthrough in object detection, utilizing transformer architecture, initially developed for natural language processing, to achieve cutting-edge performance. In contrast to traditional object detection models that rely on anchor boxes, DETR introduces a transformer-based encoder-decoder framework. This structure allows DETR to process entire images and directly predict object bounding boxes and class probabilities without needing heuristic anchors. The transformer encoder efficiently encodes the spatial information of the input image into a sequence of feature maps. At the same time, the decoder generates a set of object queries and refines their positions through iterative self-attention mechanisms. By embracing this approach, DETR streamlines the object detection pipeline and enhances accuracy and generalization across diverse datasets. Its capacity to handle objects of different scales and aspect ratios consistently makes it well-suited for complex and varied real-world applications^27^.

The Real-Time DEtection TRansformer (RT-DETR) extends DETR’s capabilities to address real-time object detection scenarios. This is achieved by introducing an efficient hybrid encoder architecture, combining convolutional neural networks (CNNs) with transformers. This approach optimizes inference speed while maintaining precise object localization across various scales and orientations. By leveraging this hybrid architecture, RT-DETR significantly accelerates inference times, making it well-suited for applications requiring rapid decision-making capabilities, such as autonomous systems and video analytics. RT-DETR represents a substantial advancement in high-performance, real-time object detection solutions, building upon the foundational achievements of DETR to effectively meet real-time operational requirements^28^.

In summary, these DL models are all advanced object detection models. Each model has unique strengths and features, and the choice of which model to use depends on the project’s specific requirements, such as accuracy, speed, deployment constraints, and available resources. Evaluating and comparing their performance on the dataset and task is recommended to determine the most suitable model for your needs.

The smaller versions of each DL model, YOLOv5n, YOLOv8n, RT-DETR-L, and DETR 50, were chosen to balance performance with computational efficiency. These compact variants offer faster inference times, lower memory usage, and efficient object detection for real-time applications like robotic pollination. Despite their smaller size, they maintain robust detection performance and are suitable for deployment on resource-constrained devices in challenging environments.

The conventional practice of partitioning data into train, validation, and test sets is a prevalent method for assessing the efficacy of DL models^29,30^. Nevertheless, this approach presents limitations, particularly when applied to small or augmented datasets. The rigid allocation of data into three distinct sets undermines the optimal utilization of available data and introduces variability in performance assessments. Addressing these concerns, k-fold cross-validation introduces a dynamic partitioning of the training and validation sets, thereby mitigating the aforementioned challenges.

The k-fold cross-validation process begins with shuffling and dividing the complete dataset into k equally sized folds^31^. During each iteration of k-fold cross-validation, one fold assumes the role of the validation set, while the remaining k − 1 folds are employed for model training. This rigorous approach ensures exposure to a diverse range of training and validation data, thereby significantly enhancing the model’s ability to generalize to unobserved datasets and mitigating the risk of overfitting on specific data subsets^31^. The method furnishes a more dependable evaluation of the model’s efficacy by averaging the performance metrics from each fold, surpassing the outcomes of a rigid conventional split. Although demanding computationally because of the recurrent training and validation cycles, k-fold cross-validation optimizes available data and the creation of resilient DL models capable of demonstrating high performance across diverse datasets^32^.

Selecting a k value of 5 offers an optimal balance between computational efficiency and robust evaluation for a dataset comprising 556 original images augmented to 3888 images. This approach divides the train + validation set into five subsets of approximately 754 images each, ensuring that every image participates in the validation set exactly once across the five iterations^33^. By rotating the validation set while training on the remaining folds in each iteration, the model encounters diverse subsets of data, enhancing its ability to generalize and reducing the risk of overfitting to specific augmented samples^34^. Despite the computational cost inherent in repeated training and validation cycles, 5-fold cross-validation balances computational efficiency and robust evaluation, providing reliable performance metrics that reflect the model’s effectiveness on unseen data^31^. This method leverages the augmented dataset’s increased variability, ensuring thorough model evaluation and optimizing its capability to perform well across different scenarios^33^.

**Fig. 8:**
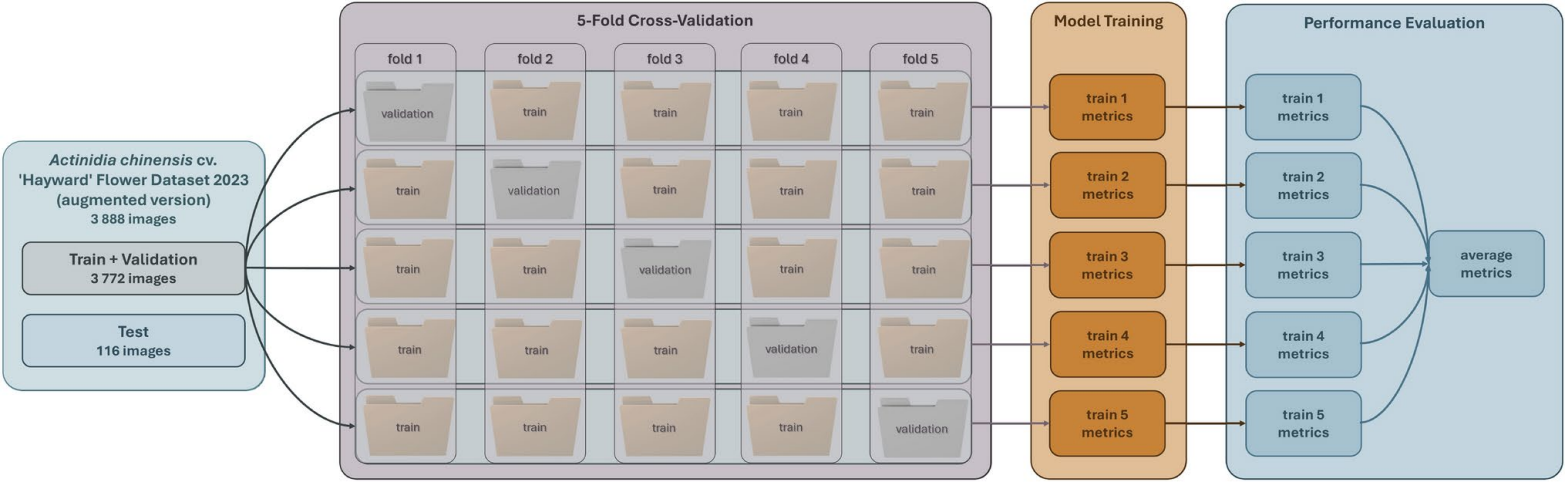
Process flow with 5-fold cross-validation method to reach DL models performance.

The train + validation set was utilised as input for the k-fold cross-validation method to precisely define the dynamic splits for the train and validation sets. Figure 8 visually represents the 5-fold cross-validation process with the *Actinidia chinensis* cv. ’Hayward’ Flower Dataset 2024 (augmented version).

The graphical representation in Fig. 8 illustrates the rigorous training process undergone by each DL model, encompassing five distinct training using different training and validation sets. Table 5 shows the division of the dataset into five folders, discriminating the number of images and annotations for each class.

**Table 5:** *Actinidia chinensis* cv. ’Hayward’ Flower Dataset 2024 (augmented version) division into five folders, with the number of images and annotated objects per class in each set.

Sets	Classes	Train 1		Train 2		Train 3		Train 4		Train 5	
		Images	Annotation	Images	Annotations	Images	Annotation	Images	Annotations	Images	Annotations
Train	Actinidia_female	1506	7124	1529	7187	1565	7364	1525	7133	1551	7288
	Actinidia_male	1521	6015	1496	5949	1455	5742	1486	5936	1478	5782
	Total	3017	13,139	3017	13,136	3018	13,106	3018	13,069	3018	13,070
Validation	Actinidia_female	413	1900	390	1837	354	1660	394	1891	368	1736
	Actinidia_male	338	1341	363	1407	404	1614	373	1420	381	1574
	Total	755	3241	755	3244	754	3274	754	3311	754	3310

As shown in Table 5, dividing the dataset into five folds did not result in any imbalance in terms of images or annotations for any class. This thorough exposure alleviates potential imbalances in individual factors, leading to more dependable performance appraisals and augmenting the model’s capacity to generalize effectively to novel, unseen data^33,34^.

Table 6 shows the characteristics of the models during the training. As anticipated, the training of DETR and RT-DETR models, due to their greater computational complexity, required significantly more memory and longer training times per epoch compared to YOLOv5 and YOLOv8, even when using the same batch size.

**Table 6:** Comparison of memory consumption, training time per epoch, and batch size for the selected models.

Model	Memory (GiB)	Time per epoch (s)	Batch size
YOLOv5	3.7	12	16
YOLOv8	3.8	12	16
DETR	40.3	121	16
RT-DETR	14.1	63	16

To assess each training outcome, the Fiftyone^35^ platform was used to analyse the predictions made by the four networks during the training, allowing comprehensive observation of their detection capabilities. The performance of each DL model was characterised by averaging the metrics from the five trainings, ensuring a comprehensive and robust evaluation.

## Results

(The metrics utilised are comprehensively analyzed and detailed in the research conducted by Pinheiro et al.^36^.)

**Fig. 9:**
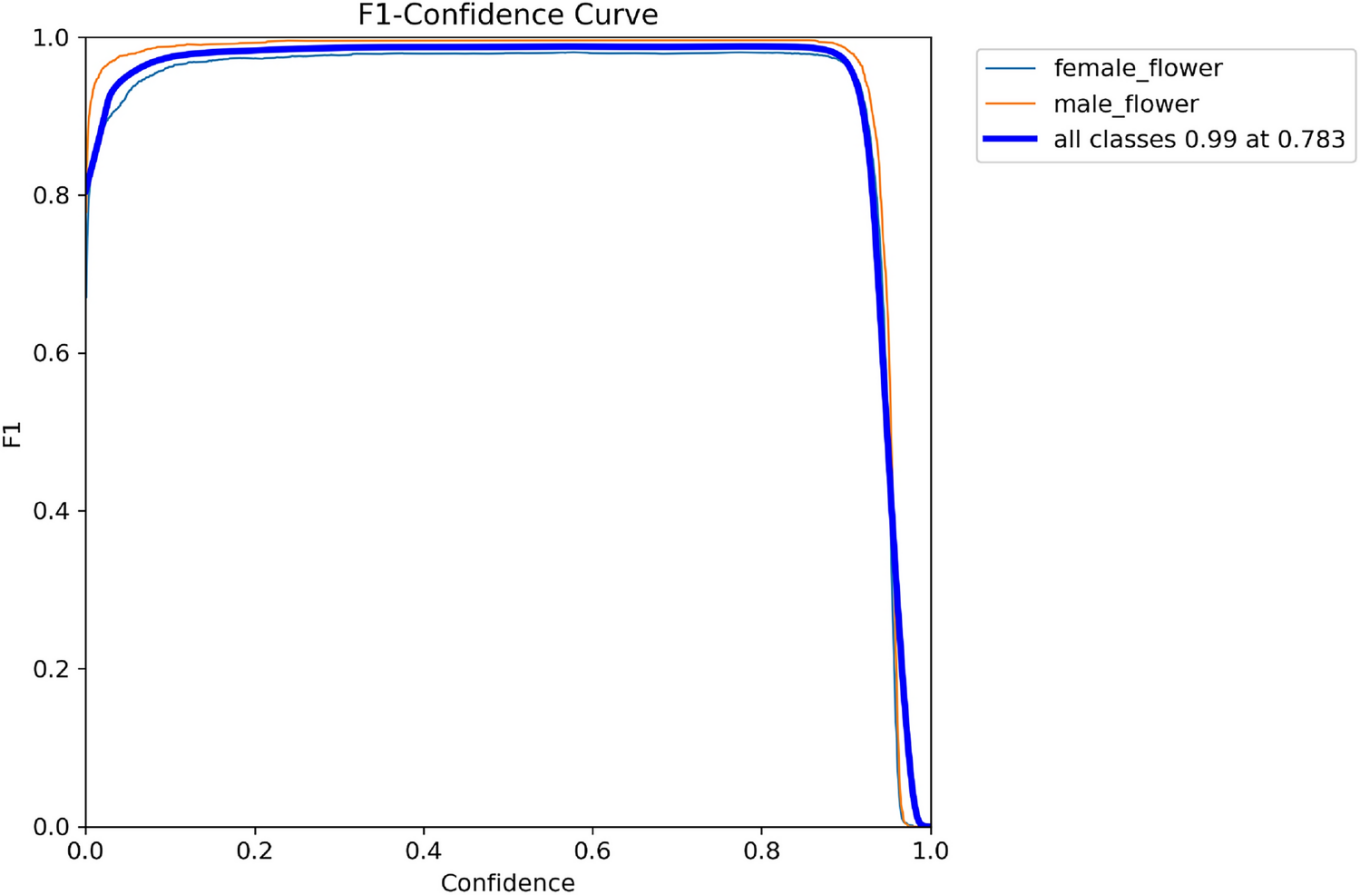
A visual representation of the F1 Score curve for the determination of the confidence threshold that optimizes the F1 Score for the YOLOv8 model train 1.

The performance metrics for each training iteration were assessed using the validation set, which was also utilised to determine the confidence threshold for inference. The confidence threshold was selected to optimize model performance by maximizing the F1 score, thereby balancing precision and recall. A high F1 score ensures that the model effectively identifies relevant instances while minimizing both the number of wrong-identified instances and reducing the number of unidentified instances. Consequently, the selected confidence threshold for each train of every model was chosen to achieve the highest possible F1 score, the best trade-off to identify the maximum number of Actinidia flower detections while minimizing the number of incorrect detections. Table 7 indicates the best F1 score and the corresponding confidence threshold for each train of each model. Figure 9 illustrates the F1 Score curve, visually representing the process of selecting the confidence threshold for the YOLOv8 model train 1.

**Table 7:** Best F1 score value and the corresponding confidence threshold for each train of every model in the validation set.

Model	Metrics	Train 1	Train 2	Train 3	Train 4	Train 5
YOLOv5	Confidence threshold (%)	72	71	72	79	65
	F1 Score (%)	99	99	99	100	99
YOLOv8	Confidence threshold (%)	78	66	79	78	77
	F1 Score (%)	99	99	99	99	99
DETR	Confidence threshold (%)	96	72	90	85	97
	F1 Score (%)	94	95	94	95	94
RT-DETR	Confidence threshold (%)	80	76	80	81	74
	F1 Score (%)	99	99	99	100	99

The confidence thresholds led to the best balance between the precision and recall metrics. As shown in Table 7, all trains performed with every model achieved a maximum F1 score above 90%, indicating a good balance between precision and recall.

To evaluate the performance of each model, the test set was utilised for inference, employing an intersection over union (IoU) of 50% and a confidence threshold that maximised the F1 score in the validation set. Precision, recall, F1 score, and mean Average Precision (mAP) were selected as the key metrics to evaluate model performance. A high precision ensures that the model accurately identifies a significant number of relevant instances while minimizing the number of wrong-identified instances. A high recall provides a large percentage of all relevant instances, thereby reducing the number of instances unidentified. Additionally, mAP quantifies the model’s effectiveness in ranking relevant instances across all classes, reflecting the overall detection quality. Table 8 indicates the average metrics of each model, with the best result achieved in each metric in bold. For detailed information about metrics results of each train iteration, consult Suplementary Table S1.

**Table 8:** Average metrics results with the test set for each model.

Model	Precision (%)	Recall (%)	F1 Score (%)	mAP (%)
YOLOv5	95	94	94	86
YOLOv8	96	93	94	85
DETR	89	97	93	94
RT-DETR	95	96	95	89

The results indicate strong performance across all considered models on the test set. YOLOv8 achieves the highest precision at 96%, while DETR excels in recall with 97% and in mAP with 94%. RT-DETR demonstrates the best F1 Score at 95%, reflecting an optimal balance between precision and recall. Although YOLOv5 and YOLOv8 show competitive precision and recall, respectively, their mAP scores are slightly lower than those of DETR and RT-DETR. These results highlight the distinct strengths of each model.

**Fig. 10:**
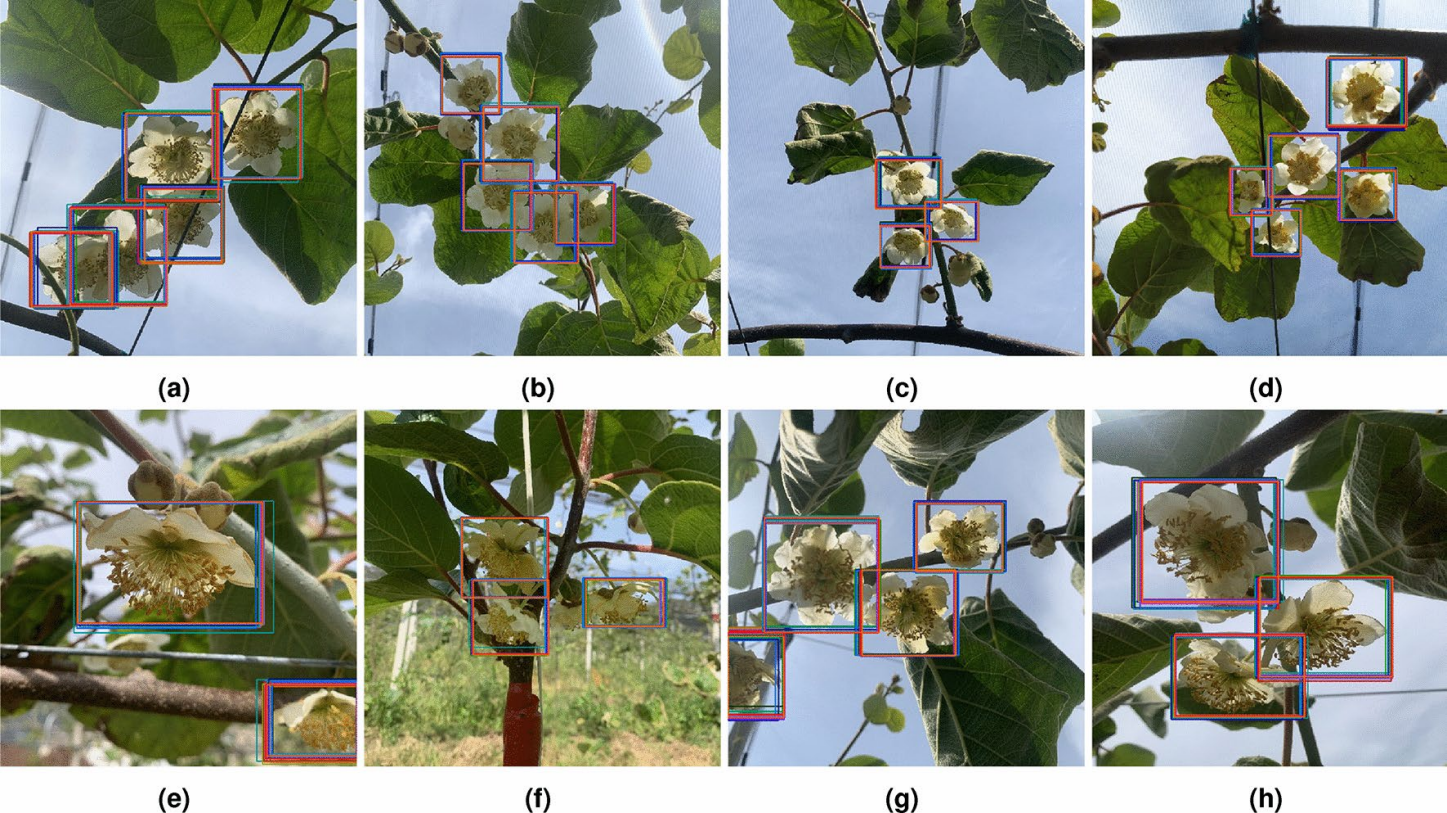
Examples of commom flower detections between YOLOv5, YOLOv8, DETR and RT-DETR in the test set. (**a**–**d**) Female flower detections. (**e**–**h**) Male flower detections.

Figure 10 displays examples of common flower detections between YOLOv5, YOLOv8, DETR and RT-DETR in the different trainings for each class. The examples presented demonstrate successful detections under challenging conditions, including overlapping flowers, varying lighting conditions, and different distances.

The metrics for each class were analysed individually to gain a deeper understanding of the results. Table 9 displays the average performance metrics for each model per class, with the best value for each metric per class highlighted in bold. For detailed information about metrics results per class of each train iteration, consult Suplementary Table S2.

**Table 9:** Average metrics with the test set for each model per class.

Model	Class	Precision (%)	Recall (%)	F1 Score (%)	AP (%)
YOLOv5	actinidia_female	95	97	96	96
	actinidia_male	97	77	86	75
YOLOv8	actinidia_female	96	96	96	96
	actinidia_male	98	76	85	75
DETR	actinidia_female	90	97	93	95
	actinidia_male	88	95	91	91
RT-DETR	actinidia_female	95	98	96	96
	actinidia_male	96	82	88	81

The results indicate that, across recall, F1 score, and AP, the models generally perform better for female Actinidia flowers than male flowers. The key findings from these results are that YOLOv8 achieves the highest precision and AP for both classes, while DETR shows notable strength in recall, F1 score and AP for male Actinidia flower. DETR was the model that achieved the most consistent results overall, with a range of values between 88 and 97%.

Precision-recall curves could provide valuable insight into the AP of each class. For precision-recall curves of each train iteration of YOLOv5, YOLOv8, DETR and RT-DETR consult Suplementary Figs. S2–S5, respectively. The precision-recall curves for each train of every model utilised in this study are shown in the annexes. For every model, the actinidia_male curve consistently shows lower precision than the actinidia_female curve, resulting in a lower AP. Notably, the DETR model demonstrates a reduced disparity between the two classes.

For the best model performance, the key is to maximize true positives (TP) and average precision (AP) while minimizing false positives (FP) and false negatives (FN). High TP and AP show effective and accurate detection and classification of relevant instances while reducing FP and FN, which indicates fewer classification errors and missed detections. Hence, achieving this balance ensures that the model performs reliably and accurately. To better understand the precision and recall metrics, it is crucial to analyse the number of FP and FN, respectively, and the number of TP. On the other hand, studying the AP for each class led to a better understanding of the mAP. Table 10 indicates the number of each type of detection and the corresponding AP for each class of each model, with the best value for each metric per class highlighted in bold. For detailed information about detection results per class of each train iteration, consult Suplementary Table S3.

**Table 10:** Average detection results with the test set for each model per class.

Model	Class	TP	FP	FN
YOLOv5	actinidia_female	435	22	15
	actinidia_male	54	2	16
YOLOv8	actinidia_female	432	18	18
	actinidia_male	53	1	17
DETR	actinidia_female	438	50	12
	actinidia_male	66	9	4
RT-DETR	actinidia_female	439	26	11
	actinidia_male	57	2	13

The results for each model with the test set should detect 521 objects, including 455 female Actinidia and 66 male Actinidia flowers (Table 4). YOLOv8 performs notably well, achieving the lowest false positives for both classes, as expected, given high precision scores. Despite having a relatively high number of FP, DETR excels with the highest TP and the lowest FN for male Actinidia flowers. RT-DETR achieves the highest number of TP and the lowest number of FN for the female Actinidia flowers. Overall, DETR was the most consistent model, achieving the highest TP and the lowest FN for male Actinidia flowers, which was the most critical class.

Analyzing FP and FN across train iterations and models is crucial for comprehensively understanding models’ performance and reliability. By systematically evaluating FP and FN, specific scenarios or conditions under which the models consistently fail can be identified, thus highlighting potential weaknesses in detection capabilities. This deeper insight facilitates targeted improvements in model architecture and training processes, ultimately leading to enhanced precision and recall. Additionally, examining FP and FN across multiple models and training runs provides a robust assessment of generalization performance, ensuring that the model’s strengths and limitations are thoroughly understood and addressed.

**Fig. 11:**
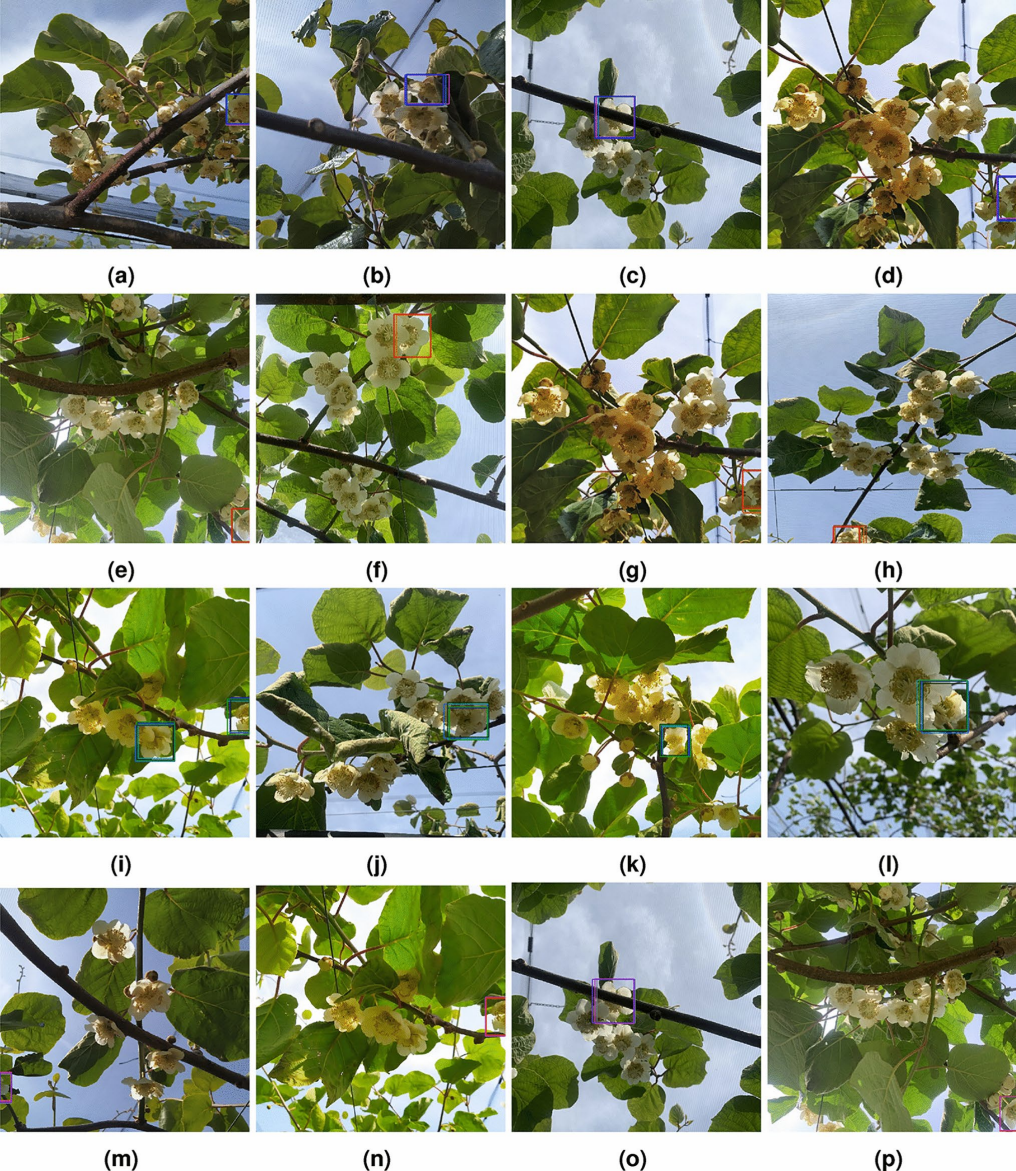
Examples of false positives in the test set for female Actinidia flowers across all training iterations. (**a**–**d**) YOLOv5. (**e**–**h**) YOLOv8. (**i**–**l**) DETR. (**m**–**p**) RT-DETR.

Figure 11 presents examples of FP in the test set for female Actinidia flowers in the four models across all training iterations.

All the examples illustrate instances where the models have identified a female Actinidia flower without visual access to the flower’s centre, where the female flower’s stigma should be. The presence of the stigma is crucial for differentiating between male and female Actinidia flowers. If this area of the flower is not present in the image, the flower should not be detected by the models.

In the test set for Actinidia female flowers, there were only two images where all models obtained FP at different training iterations. These suggest that each model identifies and learns distinct features from the training set.

**Fig. 12:**
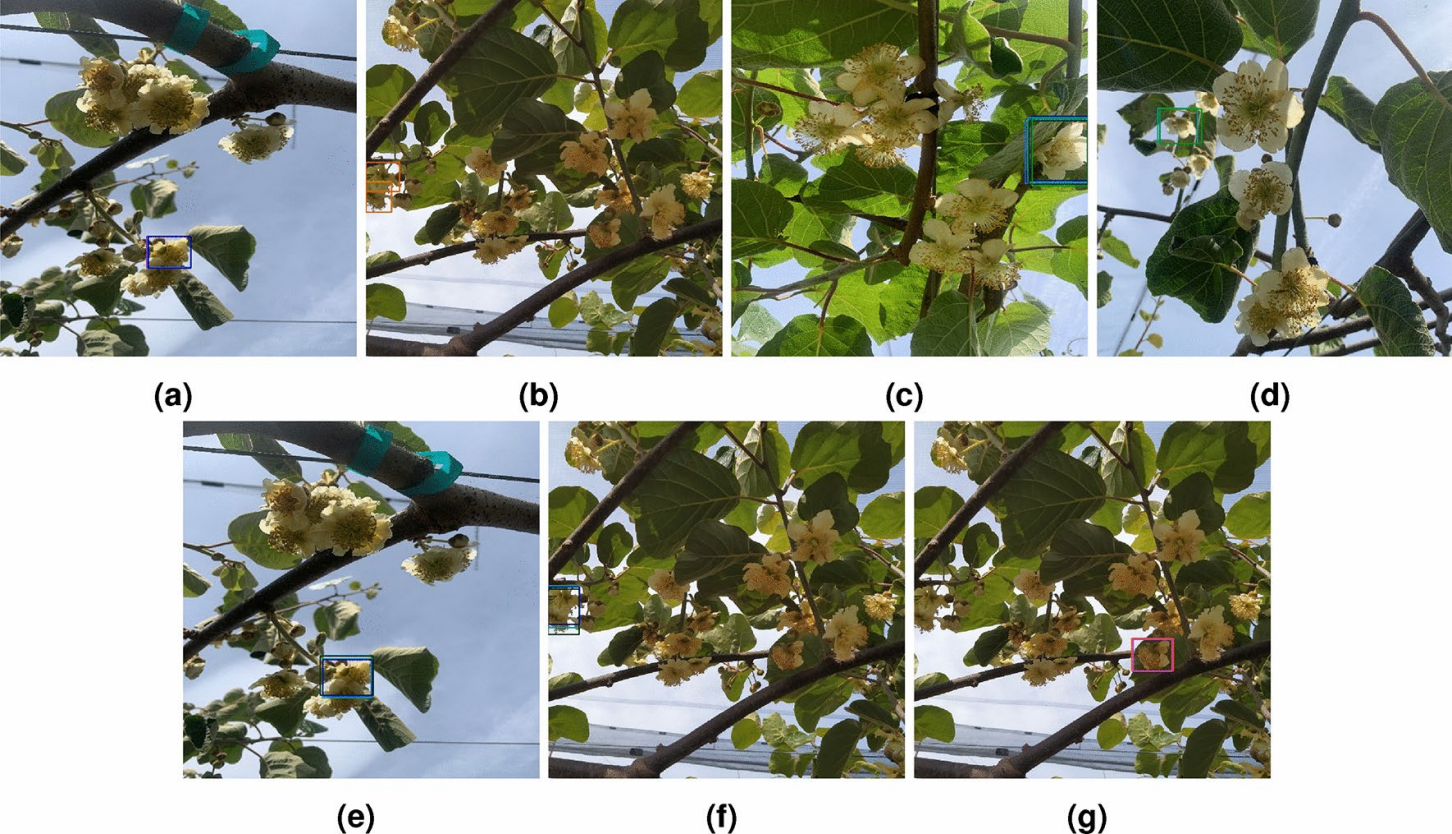
Examples of false positives in the test set for male Actinidia flowers across all training iterations. (**a**) YOLOv5. (**b**) YOLOv8. (**c**–**f**) DETR. (**g**) RT-DETR.

Figure 12 presents examples of FP in the test set for male Actinidia flowers in the four models across all training iterations.

Most of the examples demonstrate instances in which the models have detected a male Actinidia flower without visual access to the centre of the flower, which has the presence or not of the female flower’s stigma. Identifying the presence of the stigma is essential for distinguishing between female and male Actinidia flowers. Therefore, if this flower region is missing in the image, the models should not detect the flower.

The first image of DETR presents a distinct case in which a female Actinidia flower was mistakenly classified as a male Actinidia flower. This model constantly detected the female as a male Actinidia flower across all training iterations.

Evaluating YOLOv8’s performance across five training iterations on male Actinidia flowers, the model identified a total of seven FP. Notably, each FP was unique, suggesting that the model progressively adapted to the diverse examples more frequently encountered within the dataset used for training.

In the test set for Actinidia male flowers, there was no single image where all the models across all training iterations resulted in FP. This finding was in line with expectations (Table 10), as within this class, YOLOv5, YOLOv8, and RT-DETR each reported FP, with counts of two, one, and two, respectively.

**Fig. 13:**
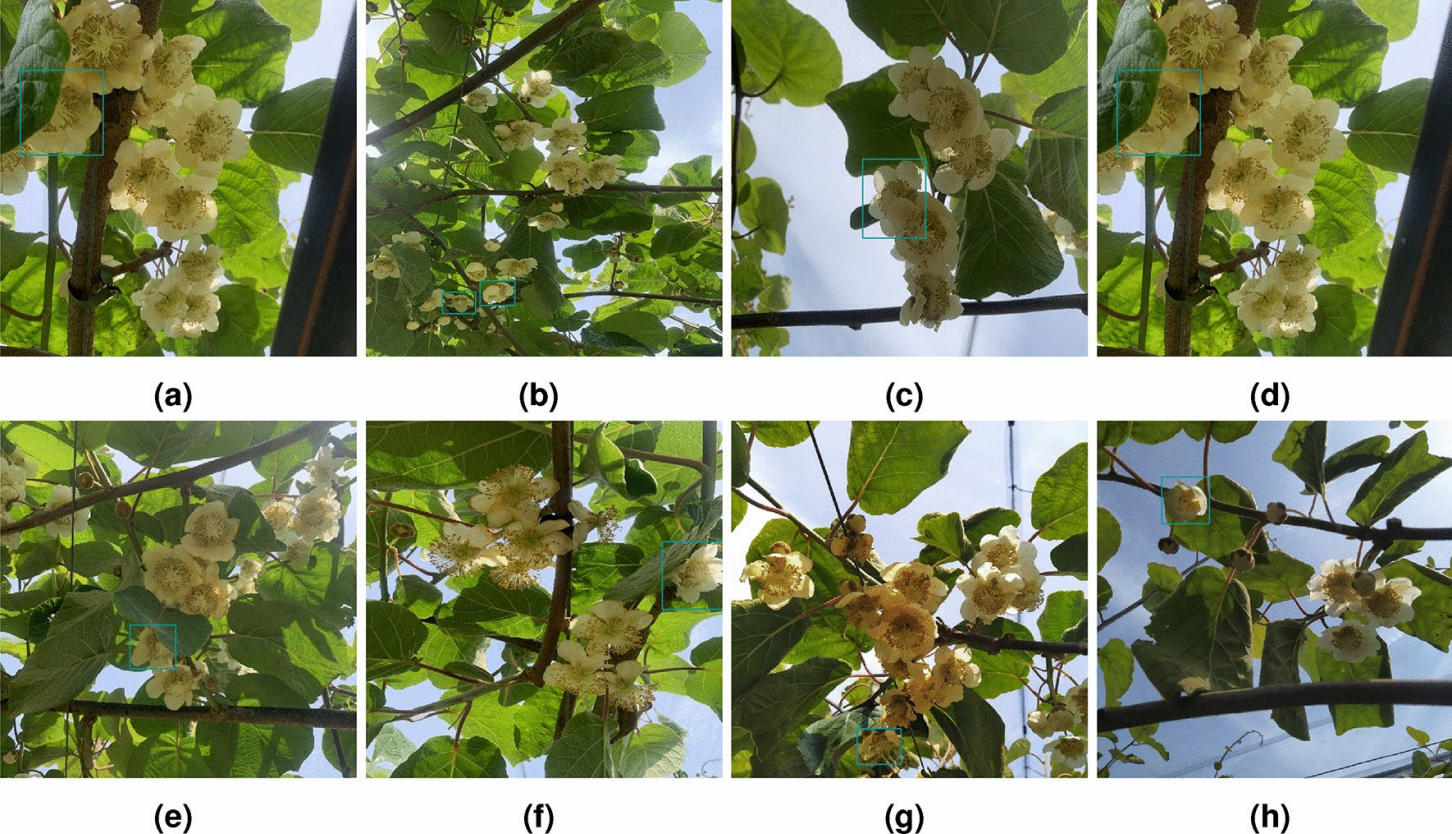
Examples of false negatives in the test set for female Actinidia flowers across all training iterations. (**a**–**c**) YOLOv5. (**d**–**e**) YOLOv8. (**f**) DETR. (**g**–**h**) RT-DETR.

Figure 13 presents examples of FN in the test set for female Actinidia flowers in the four models across all training iterations.

Examples of FN in the test set for female Actinidia flowers by YOLOv5 and YOLOv8 were often due to the occlusion of the stigma, a critical feature for distinguishing between female and male flowers. Even when this key region is only partially visible in an image, the models should ideally detect the female Actinidia flower.

In the case of DETR, the image shows a female Actinidia flower misclassified as a male Actinidia flower, corresponding to the example of DETR’s FP for the male Actinidia flower. The misclassification between the two classes generates an FP and an FN.

Lastly, RT-DETR demonstrates two distinct cases. The first involves an image depicting a female Actinidia flower, characterized by a reduced number of petals, which remained undetected. Despite the flower being in an advanced phenological stage, with a visible stigma, the model should have detected the female Actinidia flower. The second case involves an image where the stigma was partially obscured during blooming, yet RT-DETR should have been capable of detecting the female Actinidia flowers.

In the test set, no images were common among the FN obtained by all the models across all training iterations for both female and male Actinidia flowers.

**Fig. 14:**
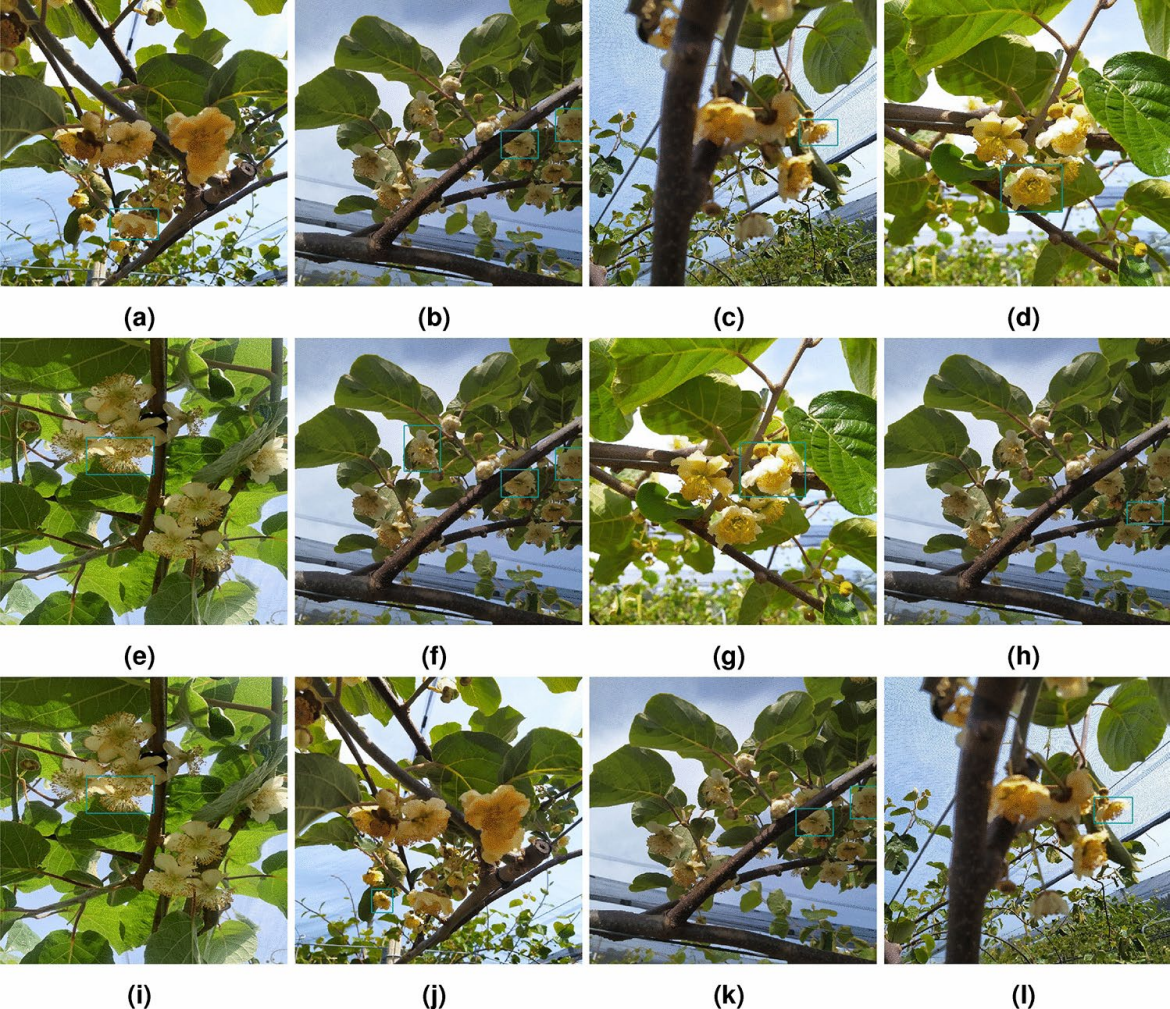
Examples of false negatives in the test set for male Actinidia flowers across all training iterations. (**a**–**d**) YOLOv5. (**e**–**g**) YOLOv8. (**h**) DETR. (**i**–**l**) RT-DETR.

Figure 14 presents examples of FN in the test set for male Actinidia flowers in the four models across all training iterations.

In the provided examples of FN in the test set for male Actinidia flowers across all models, the orientation of male Actinidia flowers results in their nondetection by the different models. Conversely, a female Actinidia flower in an identical orientation would present a visible stigma, resulting in the model incorrectly detecting the flower as a male Actinidia flower. In the test set for Actinidia male flowers, there were no images where all models obtained FN at different training iterations. These suggest that each model identifies and learns distinct features from the training set.

## Discussion

In the evaluation of the DL models for Actinidia flower detection and gender classification, various architectures were assessed, including YOLOv5, YOLOv8, DETR, and RT-DETR. The performance metrics used to measure model effectiveness included precision, recall, F1 score, and mAP. The average results (Table 8) of all models for Actinidia flower detection and gender assessment presented satisfactory results above 85% across all metrics. Optimizing the confidence threshold to maximise the F1 score proved essential for harmonising the metric results, leading to a significant decrease in FP. The models can detect Actinidia flowers and their gender in several complex scenarios, even considering occlusions, overlaps and variations in lighting conditions.

However, the average results (Table 9) of the Actinidia flower detection and gender assessment dataset in the test set for each class presented results above 75% across all metrics, with the metrics results for male Actinidia flowers being the lowest. Although YOLOv8 achieved the highest precision and AP for both classes, DETR showed notable strength in recall, F1 score and AP for male Actinidia flower. As DETR was the model that achieved the most consistent results (with a range of values between 88 and 97%), it was considered the model with the best performance.

Analyzing FP and FN across all training iterations for each model was crucial for understanding model performance and reliability. This evaluation identifies consistent failure scenarios, revealing potential weaknesses and guiding targeted model architecture and training process improvements. Most of the errors were common between all models, implying a need for new images of these specific cases to complement the dataset.

Evaluating YOLOv8’s performance across five training iterations on male Actinidia flowers revealed unique FP. This pattern suggests that the model progressively adapted to the diverse examples encountered in the training dataset. The slight increase in the dataset with images of male Actinidia flowers could increase YOLOv8 metrics, surpassing DETR.

Although DETR was considered the best network under the conditions presented, YOLOv8 was considered a very promising network with an increase in the dataset of images of male Actinidia flowers. In this way, YOLOv8 could become more interesting, as it has a shorter training time and less need for GPU memory (Table 6).

Table 11 summarizes the results from both the state-of-the-art literature and those obtained in this study, facilitating a comparison of model performances. This comparison is crucial for evaluating the significance of the results and identifying potential areas for improvement. Notably, none of the existing studies in the literature have addressed the task of detecting flowers by gender-a more complex challenge than merely detecting flowers. Gender classification requires first detecting the flowers and then accurately categorizing them based on gender.

**Table 11:** Comparison between the proposed models and the state-of-the-art DL for flower detection.

Application	DL models	Results					Article
		Precision (%)	Recall (%)	F1 score (%)	AP (%)	mAP (%)	
		Flower	Flower		Flower		
Flower and stamen detection	Modified YOLOv5s	96.7	89.1	–	–	90.1	^16^
	Faster R-CNN + ResNet50	57.4	98.9	–	–	92.6	
	Faster R-CNN + VGG	68.5	98.9	–	–	92.6	
	SDD + VGG	76.6	87.4	–	–	82.3	
	SDD MobileNetv2	86.7	70.2	–	–	81.1	
Flower and bud detection	YOLOv4	–	–	–	92.47	91.49	^17^
	YOLOV3	–	–	–	85.73	80.98	
Flower detection	Faster R-CNN NAS	96.8	68.0	79.0	–	–	^19^
	Faster R-CNN Inception v2	90.4	75.8	82.0	–	–	
	SDD Inception v2	78.5	61.2	68.1	–	–	
Flower detection	Faster R-CNN Inception v2	91	80	85	–	–	^20^
Flower and bud detection	YOLOv5l	–	–	–	93.12	93.23	^21^
Flower and gender detection	YOLOv5	95	94	94	–	86	Proposed
	YOLOv8	96	93	94	–	85	
	DETR	89	97	93	–	94	
	RT-DETR	95	96	95	–	89	

The models proposed for detecting flowers and assessing gender demonstrated superior performance over several state-of-the-art models that are limited to flower detection, as evaluated across various metrics. Notably, the DETR model outperformed all other models in achieving the highest F1 score and mAP. Although precision was exceeded by models from Zhou et al.^16^, Li et al.^17^ and Williams et al.^20^, DETR maintained strong overall performance, with consistent results ranging between 89% and 97% across all evaluated metrics. This consistency emphasizes DETR’s robustness and efficacy in executing tasks related to flower and gender detection.

While previous research has focused predominantly on general flower detection, our study introduces a novel dimension by incorporating flower gender assessment. This advancement represents a significant innovation in the field. Our experiments demonstrated that the DETR delivered the highest recall and balanced performance across metrics. These findings underscore the effectiveness and originality of our approach in both flower detection and gender classification, marking a substantial advancement beyond the scope of existing literature.

The results were achieved using the acquired dataset, which is very diverse, with different perspectives and lighting conditions. This diversity of information is crucial for developing a robust dataset. The selection of augmentation operations was also a fundamental aspect of obtaining the metrics presented. Compared to the other displayed articles, the datasets acquired do not include 5000 images, and some do not use augmentation operations.

## Conclusions

This study advances beyond previous research by focusing on detecting Actinidia flowers and assessing their gender. This capability is crucial for optimizing pollination practices. By distinguishing between male and female flowers, it becomes possible to collect pollen from males for future pollination of females, reducing production costs and mitigating the risks associated with disease transmission between plants.

In this paper, we outline a comprehensive methodology for acquiring images and applying four pretrained models to identify and classify the gender of Actinidia flowers (actinidia_female or actinidia_male). Considering class balance, an augmented dataset comprising 3,888 images of Actinidia flowers was developed as the DL training models require extensive data. K-fold cross-validation was implemented for model validation, thereby augmenting the reliability of our findings and facilitating a thorough evaluation of model performance across various data subsets.

In the evaluation of DL models for Actinidia flower detection and gender assessment, YOLOv5, YOLOv8, DETR, and RT-DETR were assessed using metrics such as precision, recall, F1 score, and mAP. All models demonstrated relevant performance, with average results exceeding 85% across these metrics, effectively detecting Actinidia flowers and their gender under complex conditions, including occlusions, overlaps, and varying lighting.

Despite these, the test set results showed that performance for male Actinidia flowers was lower than female Actinidia flowers, with all metrics remaining above 75%. YOLOv8 achieved the highest precision and AP for both classes, while DETR excelled in recall, F1 score, and AP for male flowers. DETR’s consistent performance, with results ranging from 88% to 97%, marked it as the top-performing model.

YOLOv8 showed unique FP for male Actinidia flowers, which suggests that can adapted progressively to diverse examples in the training dataset. Increasing the dataset with additional male Actinidia images could further enhance YOLOv8’s performance, making it a promising alternative given its shorter training times and lower GPU memory requirements than DETR.

Future research initiatives should precede expanding the dataset to encompass a more balanced representation of male flowers. Integrating public datasets is imperative to enhance variability and mitigate potential risks of overfitting. Furthermore, the separate classification of anthers and stigmas by gender can significantly enhance the efficiency of pollen collection and pollination activities. This contribution aims to reinforce the sustainable development and high-quality production of Actinidia fruits by optimizing the essential pollination phase, directly influencing fruit size and quality.

The detection of flowers and gender assessment remains a relatively underexplored area, underscoring the need to delineate future work, which can be bifurcated into two key steps. Firstly, augmenting the male flower dataset is imperative to rectify class imbalances. The assimilation of public datasets is essential for augmenting variability and mitigating the risk of model overfitting. Subsequently, the gender-based segmentation of anthers and stigmas is poised to enhance the efficiency of pollen collection and pollination tasks. This effort is designed to bolster sustainable development and facilitate the high-quality production of Actinidia fruits. These advancements are instrumental in ensuring the success of the pollination phase, a pivotal stage in this plant’s lifecycle, given that the quantity of pollinated seeds directly influences the size and quality of Actinidia fruits.

## Supplementary Information

Supplementary Information 1.

## Data Availability

The data presented in this study are openly available in the digital repository Zenodo: Actinidia chinensis cv. ’Hayward’ Flower Dataset 2024 (augmented version)—https://doi.org/10.5281/zenodo.13692222 (accessed on 5 September 2024).
