# Supplementary figures and images for: Deep learning based approach for actinidia flower detection and gender assessment (part 2 of 2)
Source: Sci Rep. 2024 Oct 18;14:24452. doi: 10.1038/s41598-024-73035-1 (PMC11489756; doi:10.1038/s41598-024-73035-1)

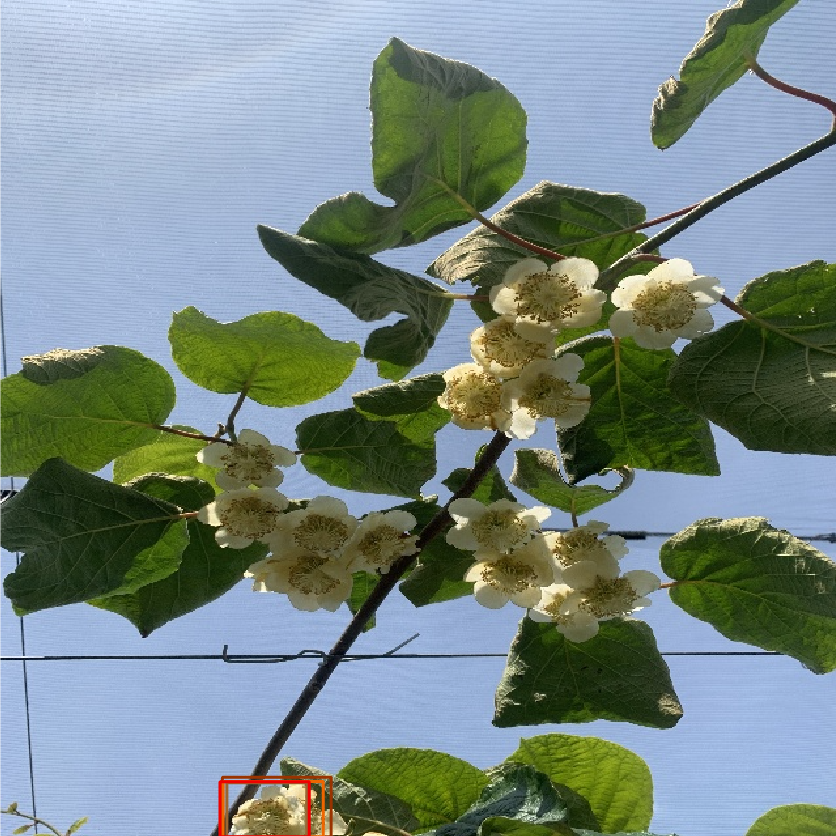

Supplement: Supplementary file 1 — Supplementary Information 1. [file 41598_2024_73035_MOESM1_ESM.zip › images/yolov8_female_fp4.png]

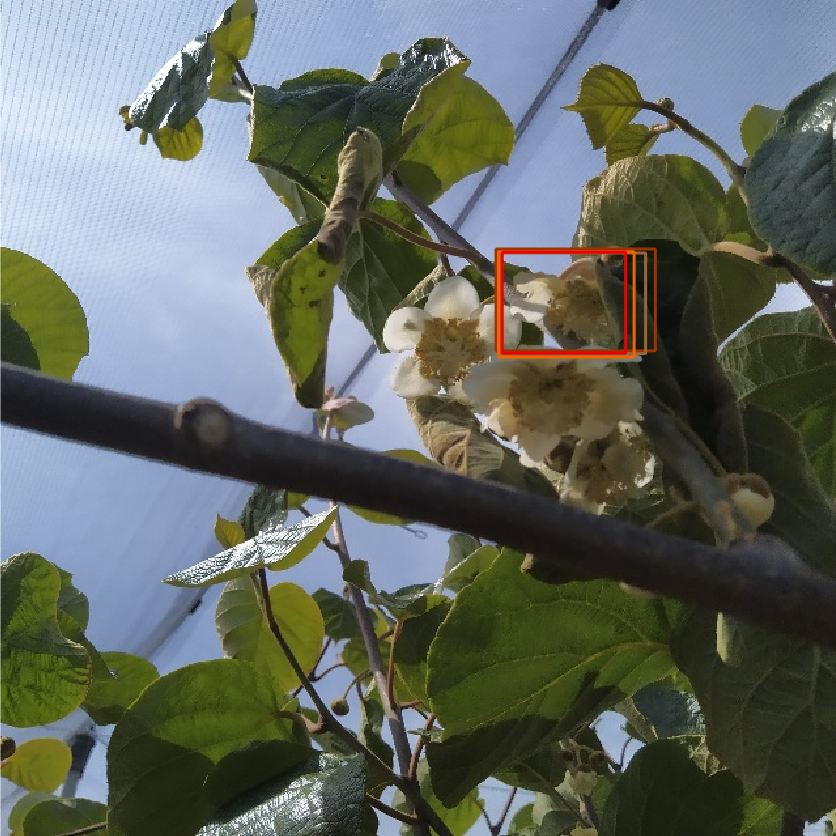

Supplement: Supplementary file 1 — Supplementary Information 1. [file 41598_2024_73035_MOESM1_ESM.zip › images/yolov8_female_fp5.png]

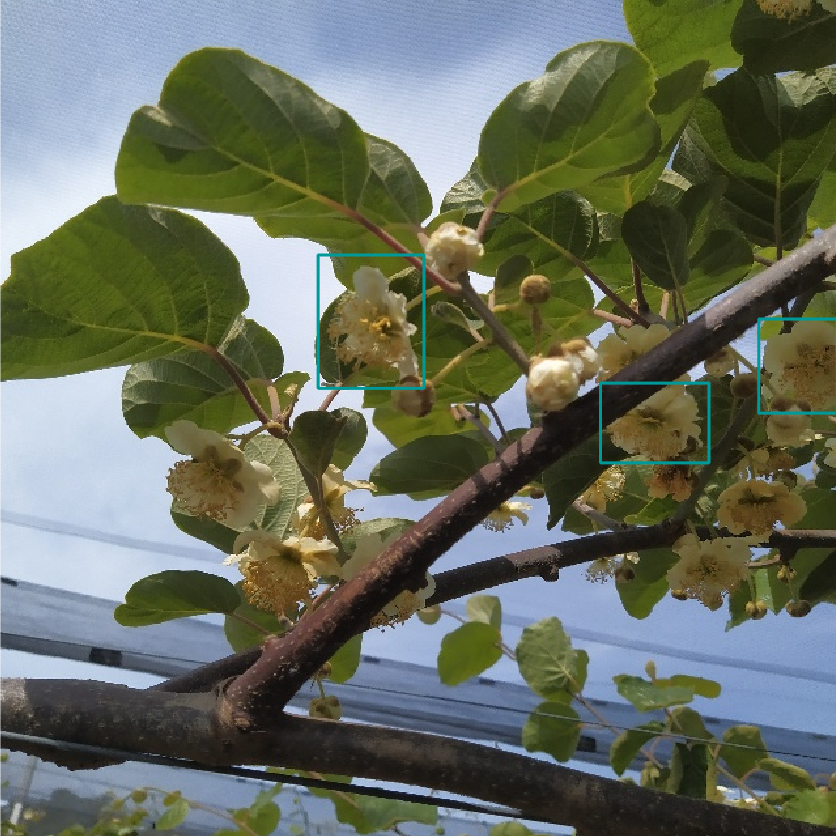

Supplement: Supplementary file 1 — Supplementary Information 1. [file 41598_2024_73035_MOESM1_ESM.zip › images/yolov8_male_fn2.png]

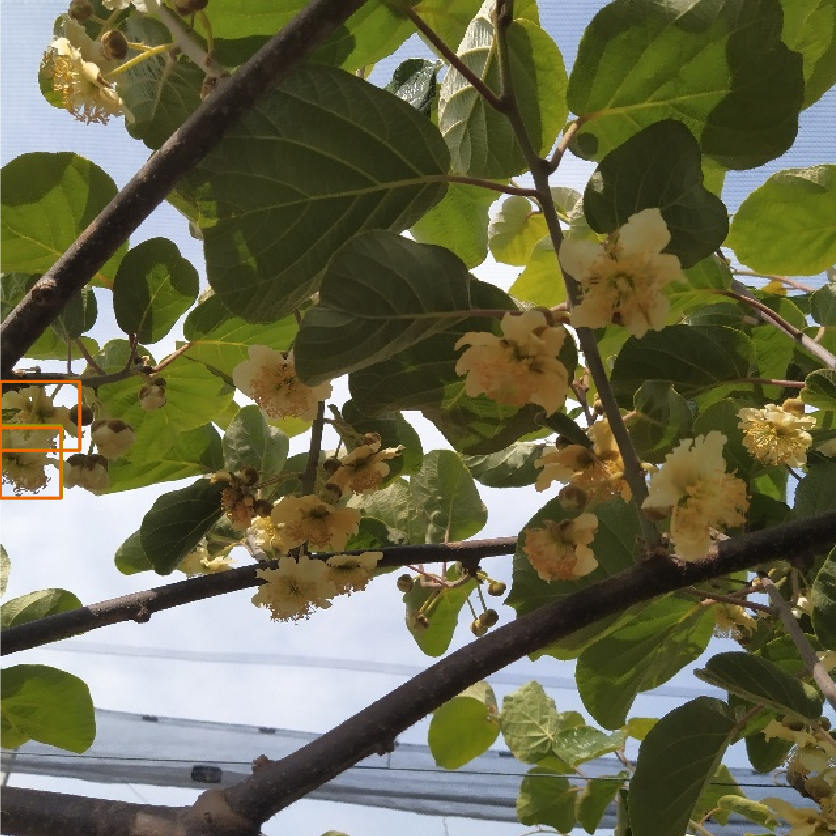

Supplement: Supplementary file 1 — Supplementary Information 1. [file 41598_2024_73035_MOESM1_ESM.zip › images/yolov8_male_fp1.png]

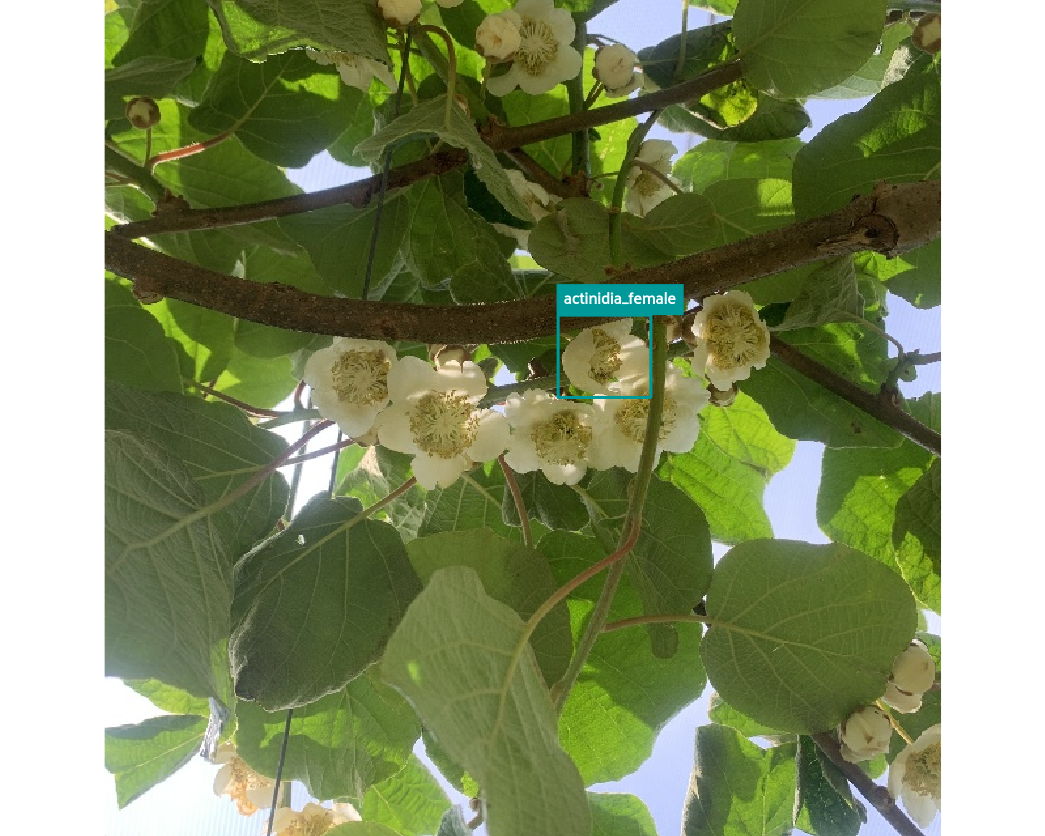

Supplement: Supplementary file 1 — Supplementary Information 1. [file 41598_2024_73035_MOESM1_ESM.zip › images/all_female_fn1.png]

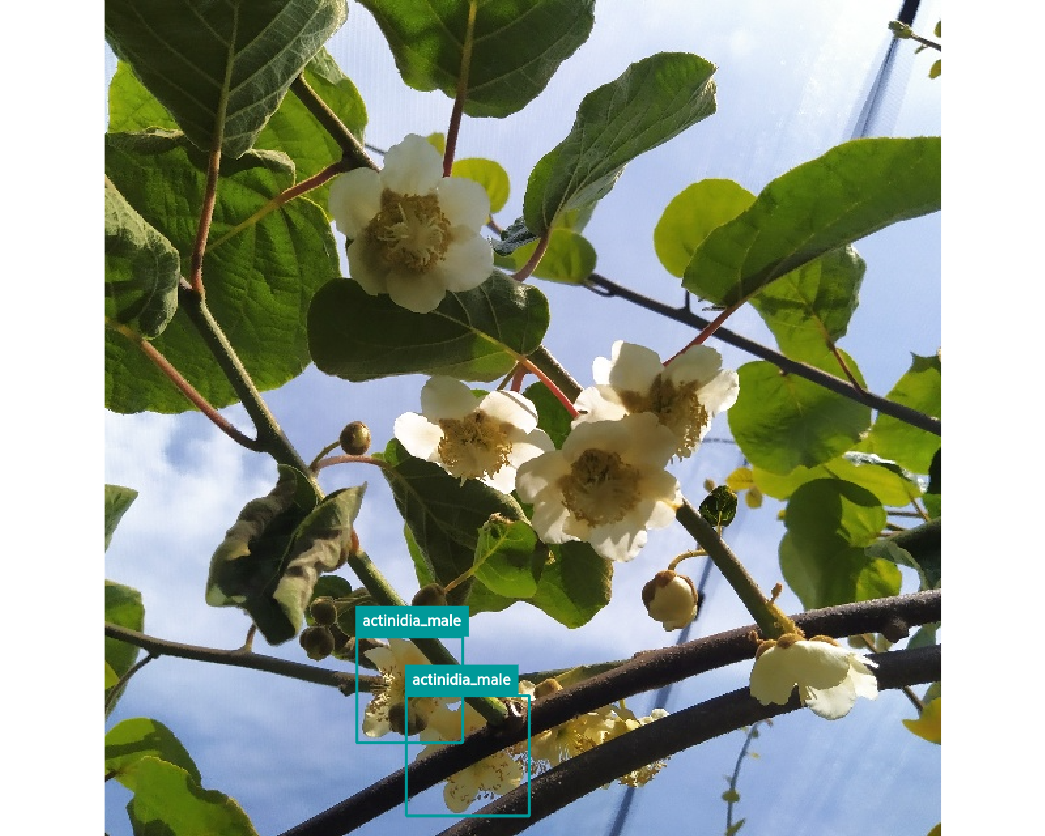

Supplement: Supplementary file 1 — Supplementary Information 1. [file 41598_2024_73035_MOESM1_ESM.zip › images/all_male_fn1.png]

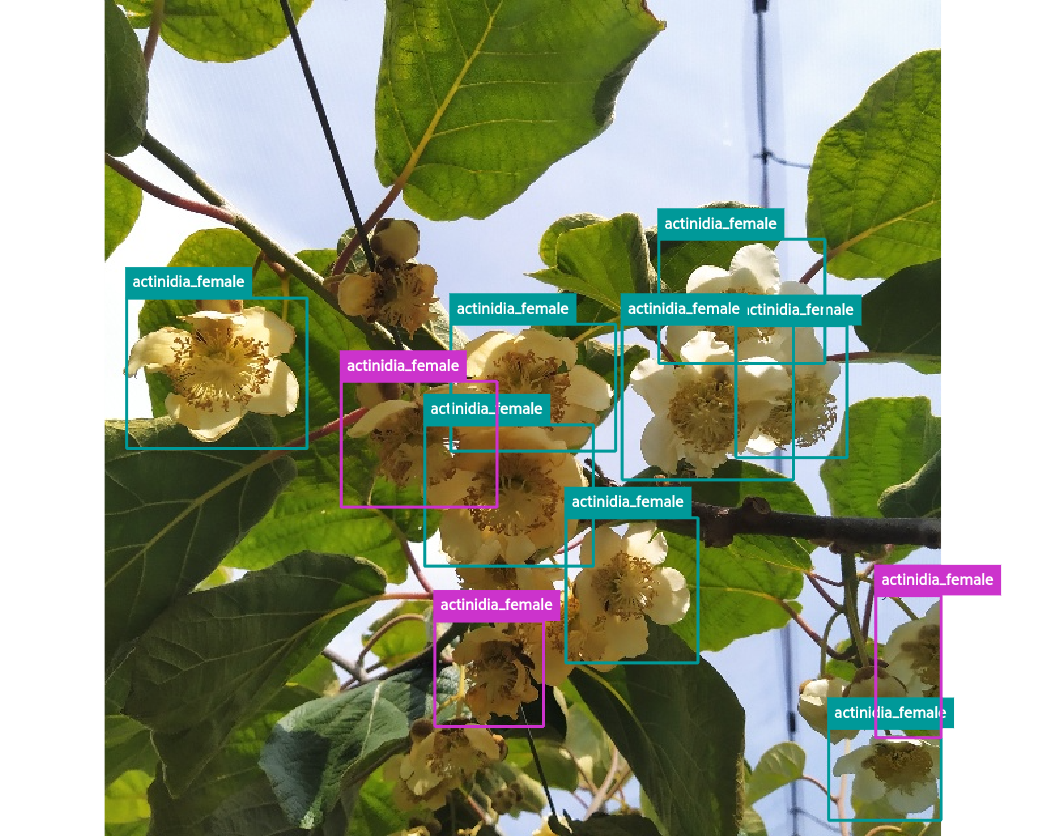

Supplement: Supplementary file 1 — Supplementary Information 1. [file 41598_2024_73035_MOESM1_ESM.zip › images/all_female_fp1.png]

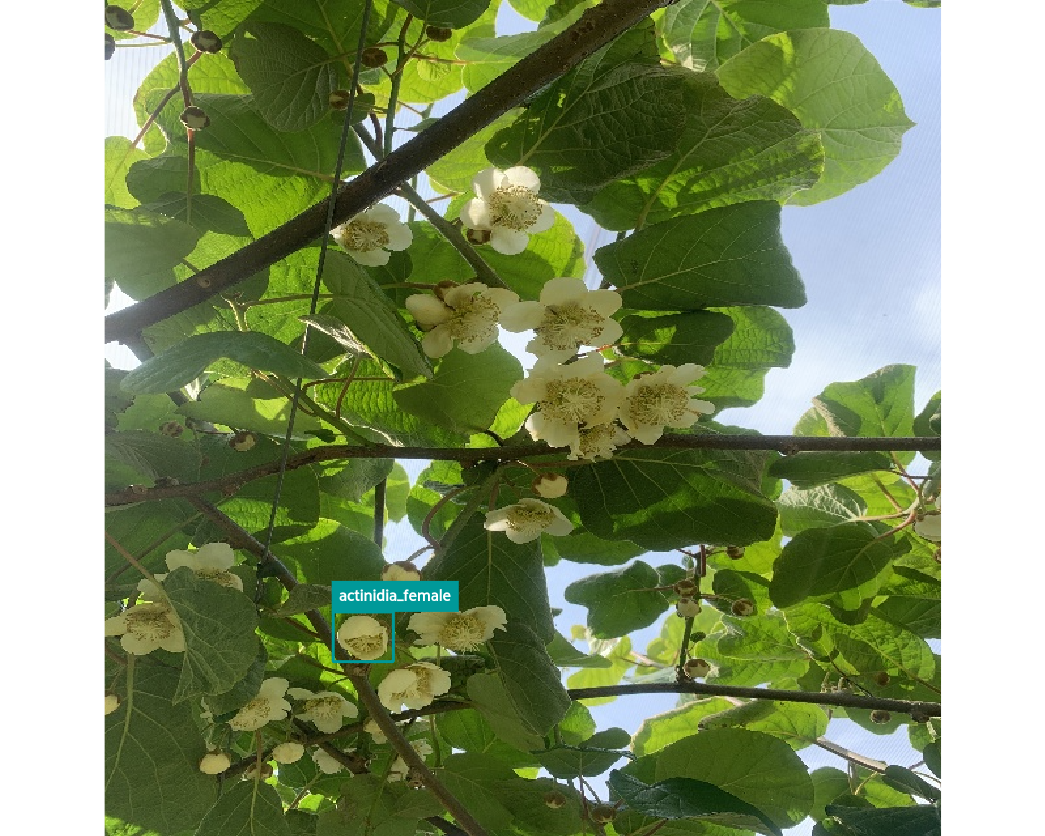

Supplement: Supplementary file 1 — Supplementary Information 1. [file 41598_2024_73035_MOESM1_ESM.zip › images/all_female_fn3.png]

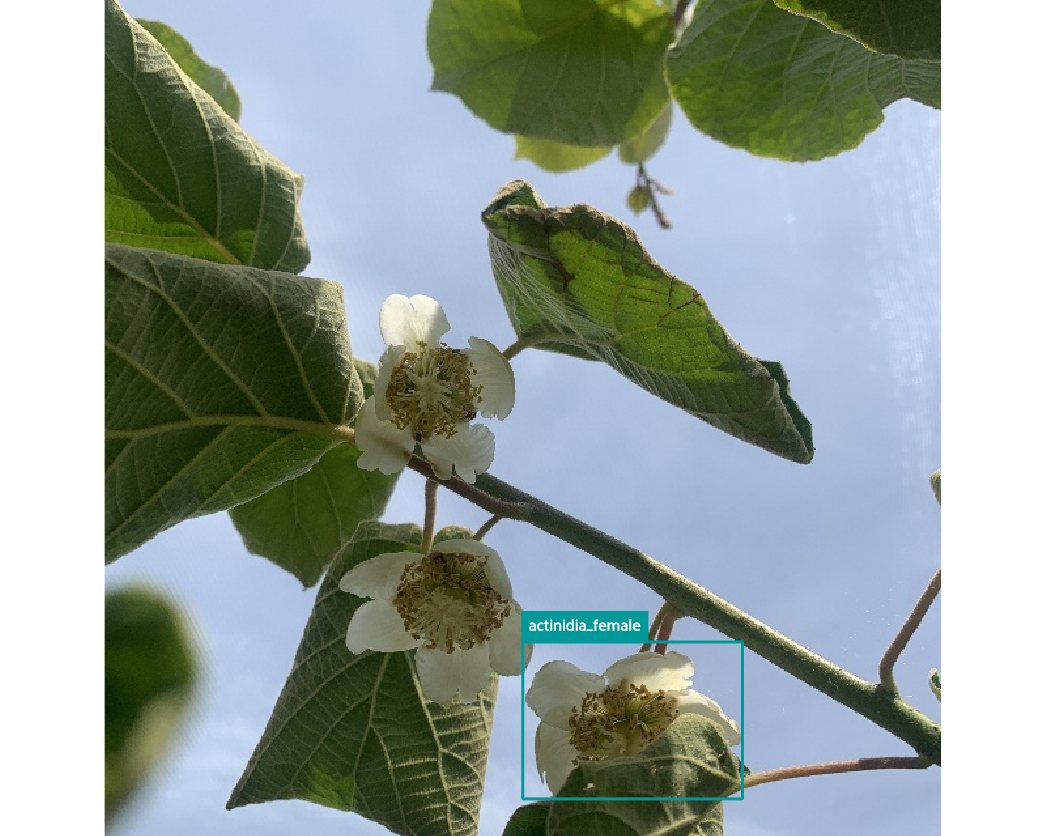

Supplement: Supplementary file 1 — Supplementary Information 1. [file 41598_2024_73035_MOESM1_ESM.zip › images/all_female_fn2.png]

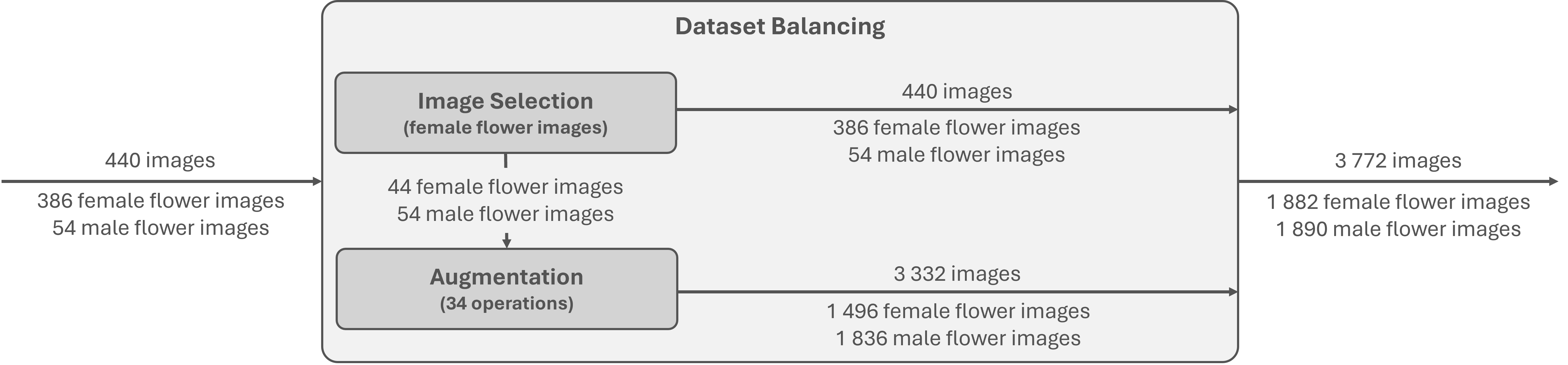

Supplement: Supplementary file 1 — Supplementary Information 1. [file 41598_2024_73035_MOESM1_ESM.zip › images/balancing.png]
