# Supplementary figures and images for: Deep learning based approach for actinidia flower detection and gender assessment (part 1 of 2)
Source: Sci Rep. 2024 Oct 18;14:24452. doi: 10.1038/s41598-024-73035-1 (PMC11489756; doi:10.1038/s41598-024-73035-1)

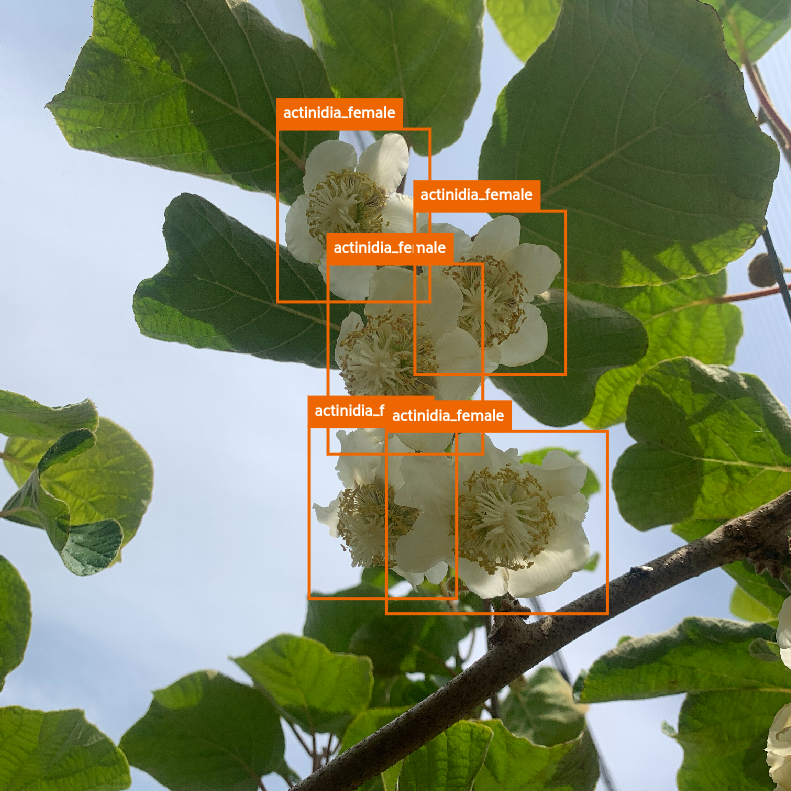

Supplement: Supplementary file 1 — Supplementary Information 1. [file 41598_2024_73035_MOESM1_ESM.zip › images/actinidia_female.png]

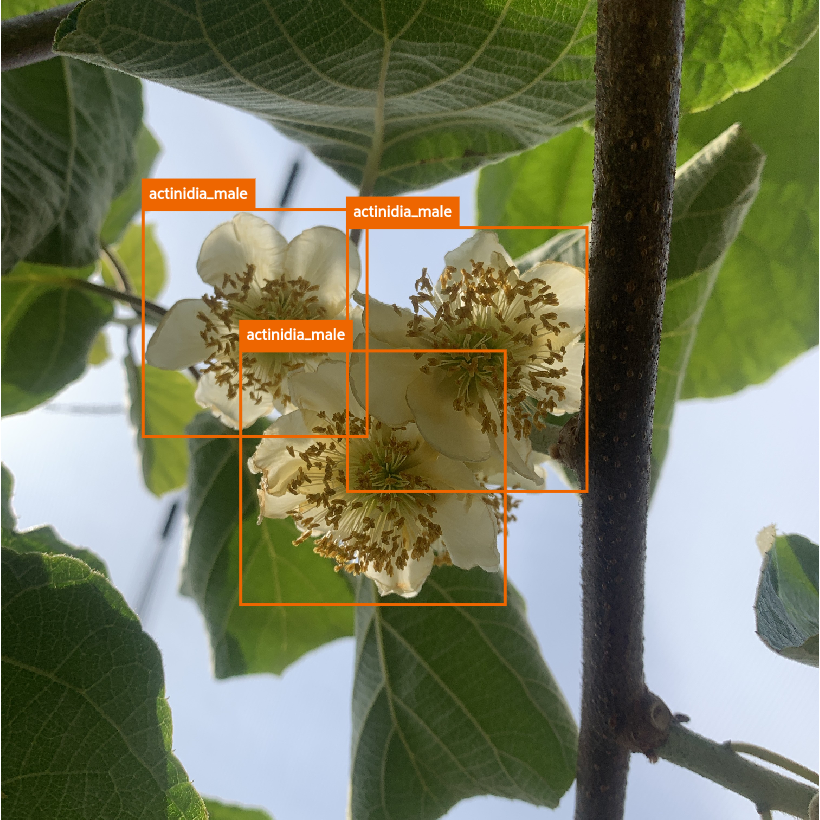

Supplement: Supplementary file 1 — Supplementary Information 1. [file 41598_2024_73035_MOESM1_ESM.zip › images/actinidia_male.png]

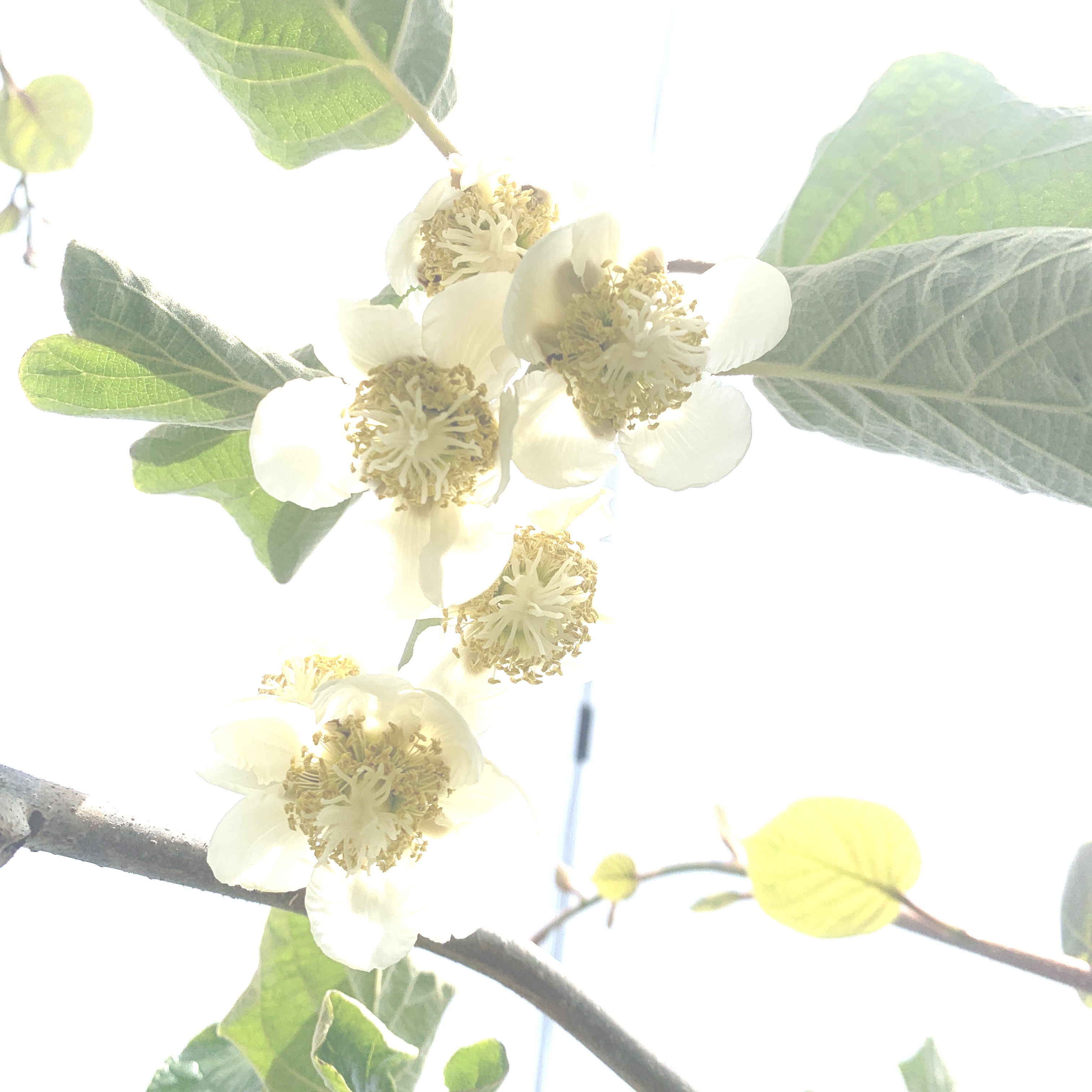

Supplement: Supplementary file 1 — Supplementary Information 1. [file 41598_2024_73035_MOESM1_ESM.zip › images/bright_contrast__female.jpg]

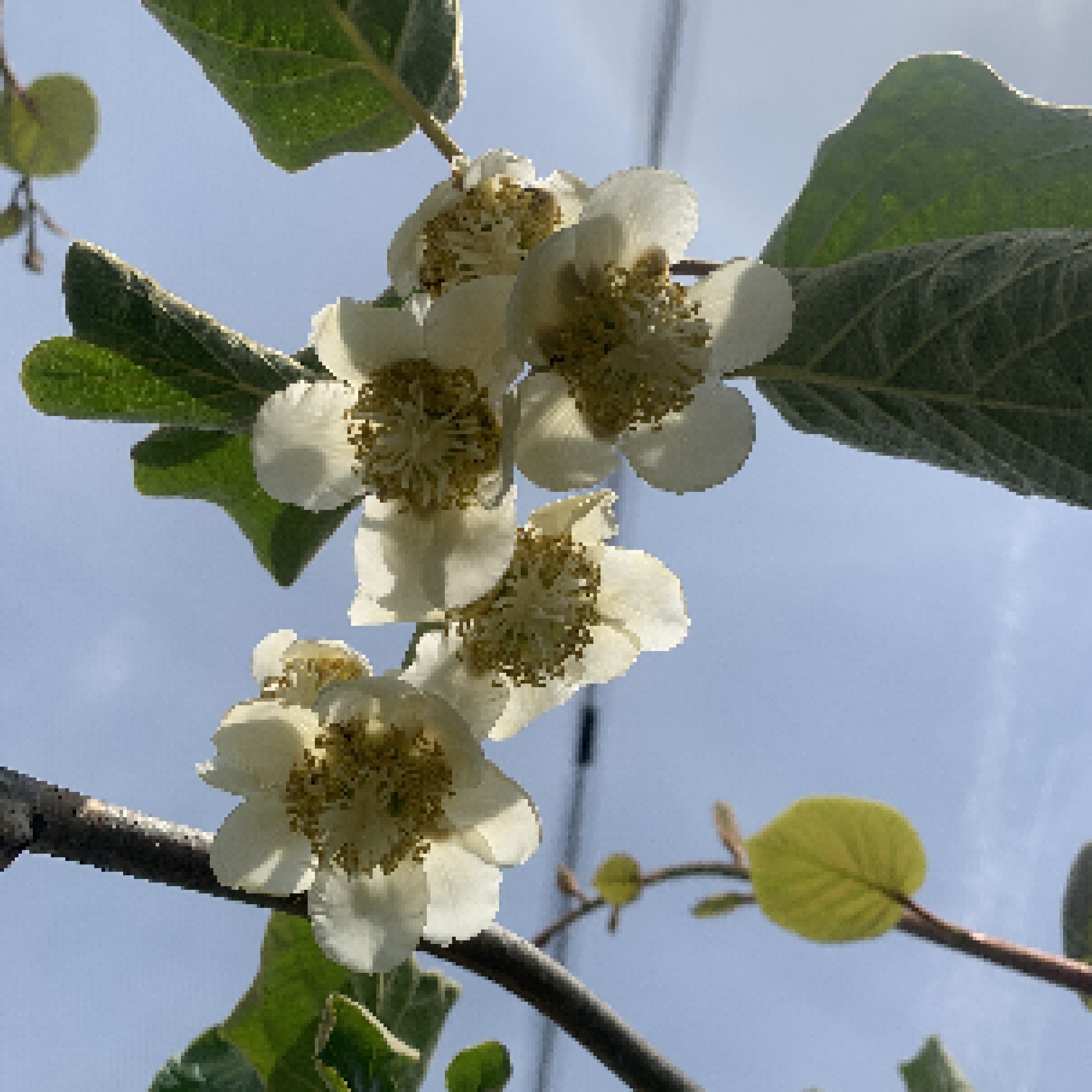

Supplement: Supplementary file 1 — Supplementary Information 1. [file 41598_2024_73035_MOESM1_ESM.zip › images/downscale__female.jpg]

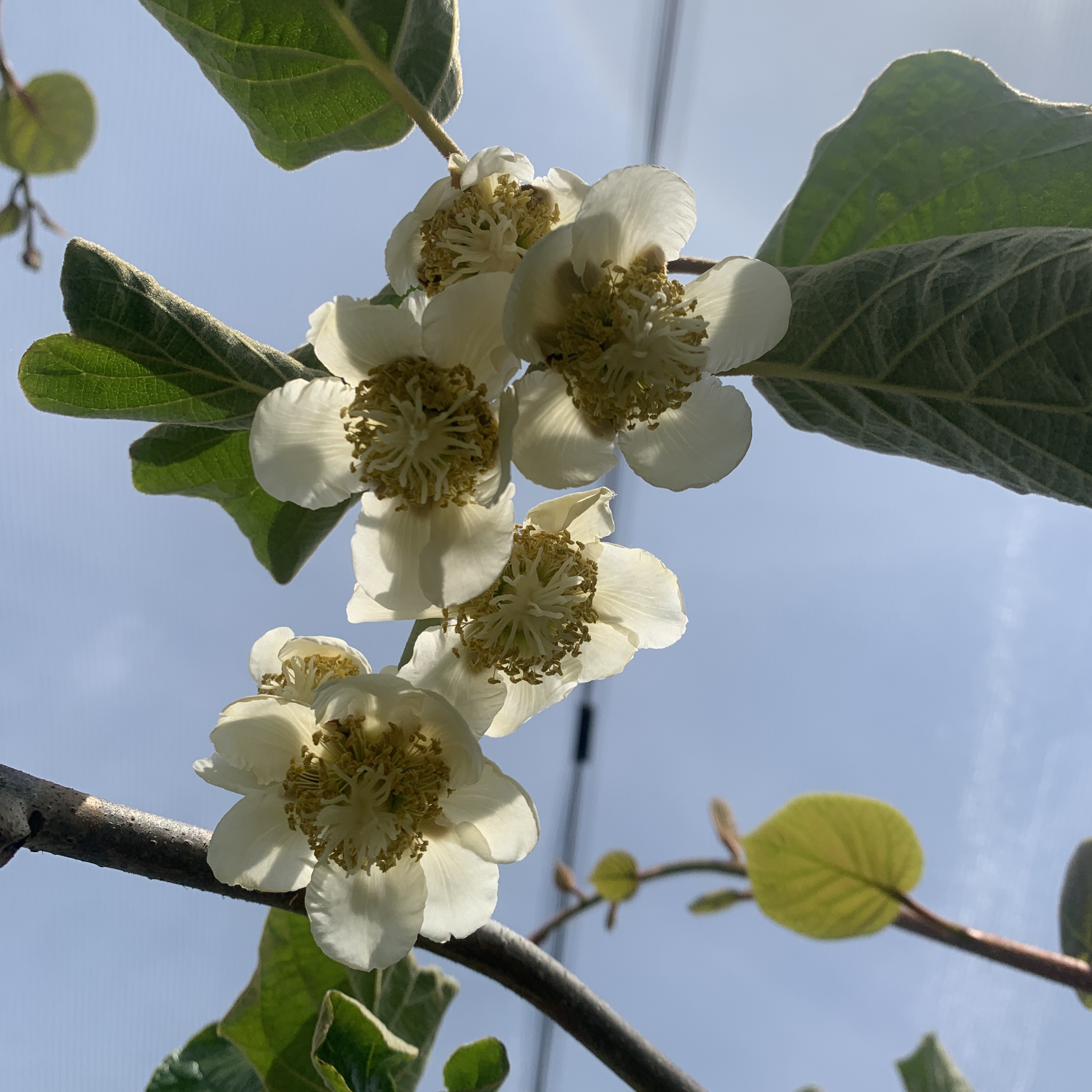

Supplement: Supplementary file 1 — Supplementary Information 1. [file 41598_2024_73035_MOESM1_ESM.zip › images/female.jpg]

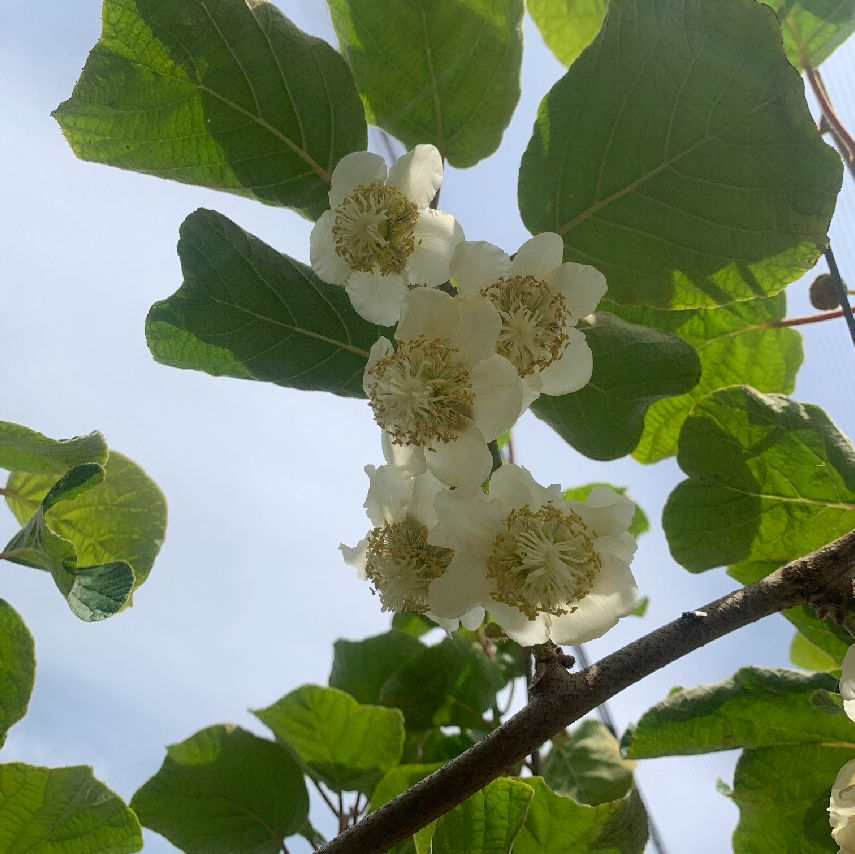

Supplement: Supplementary file 1 — Supplementary Information 1. [file 41598_2024_73035_MOESM1_ESM.zip › images/female.png]

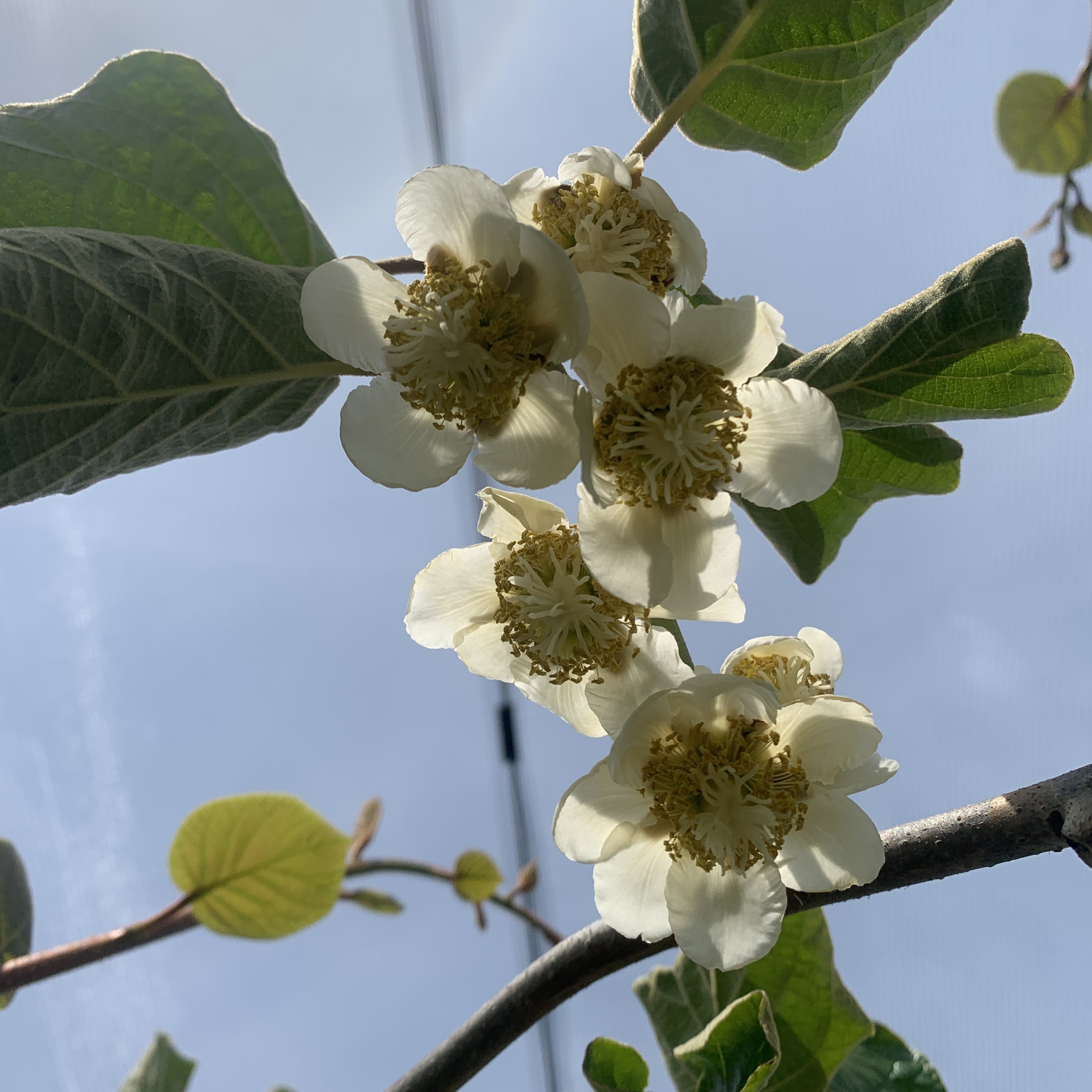

Supplement: Supplementary file 1 — Supplementary Information 1. [file 41598_2024_73035_MOESM1_ESM.zip › images/flip__female.jpg]

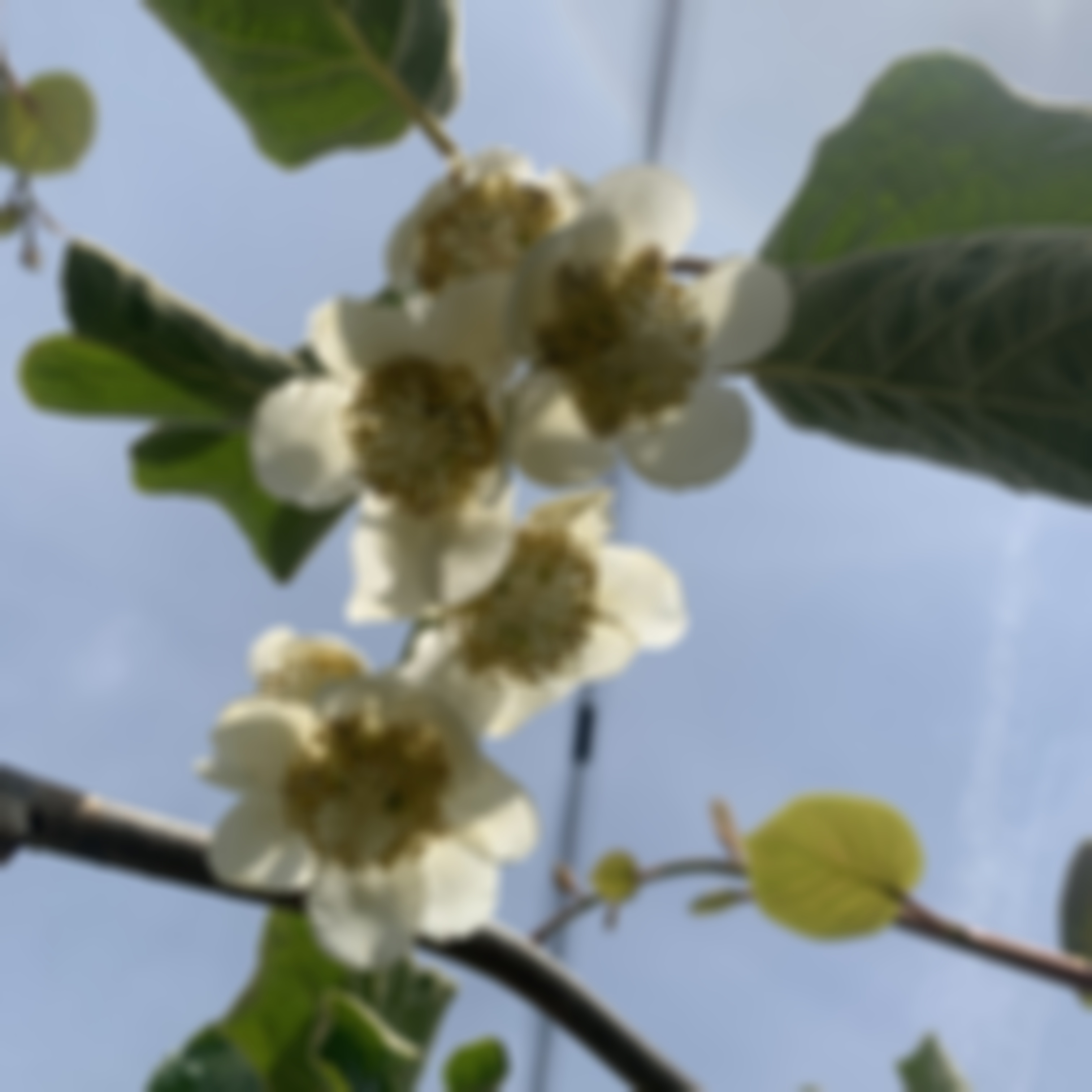

Supplement: Supplementary file 1 — Supplementary Information 1. [file 41598_2024_73035_MOESM1_ESM.zip › images/gaussian_blur__female.jpg]

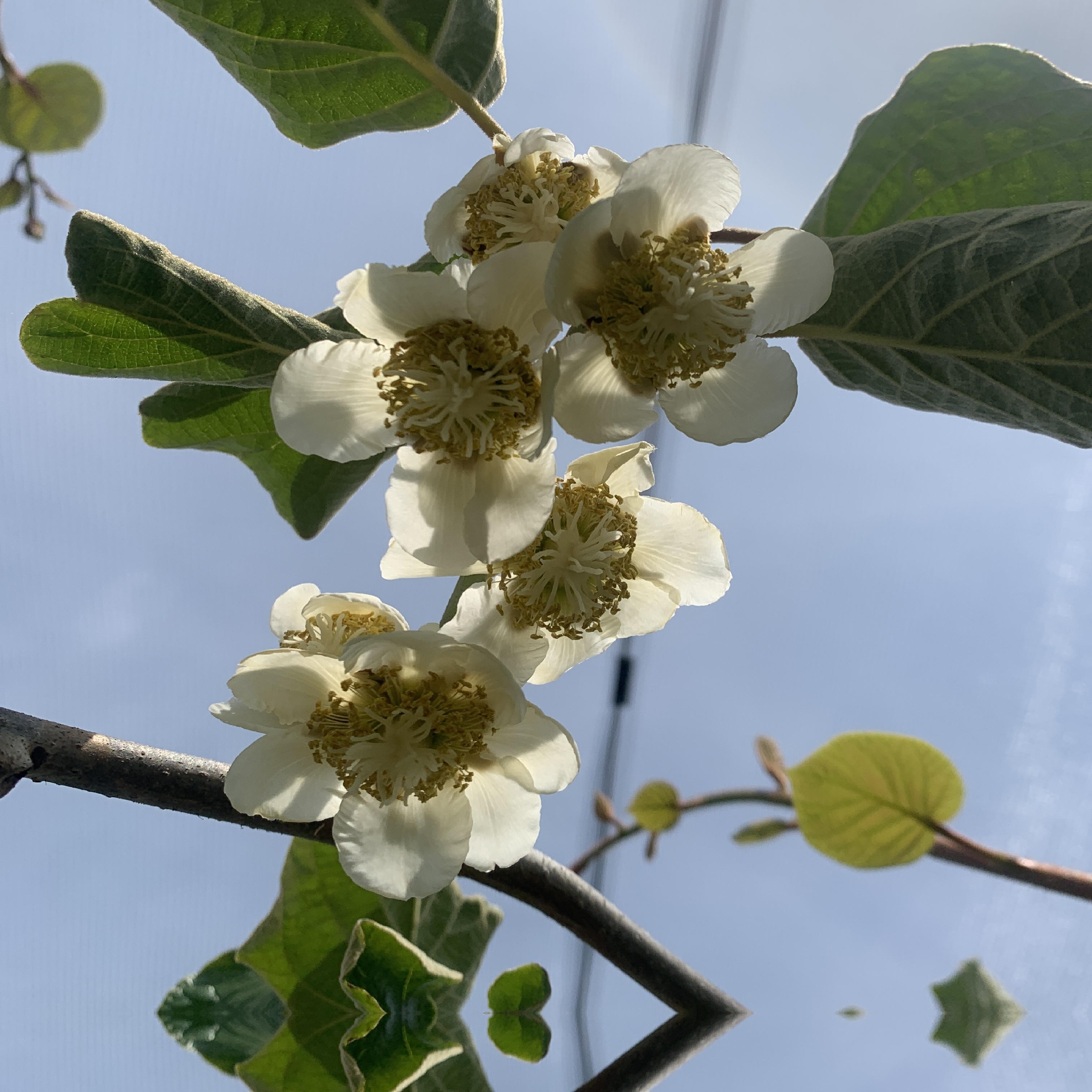

Supplement: Supplementary file 1 — Supplementary Information 1. [file 41598_2024_73035_MOESM1_ESM.zip › images/grid_distortion__female.jpg]

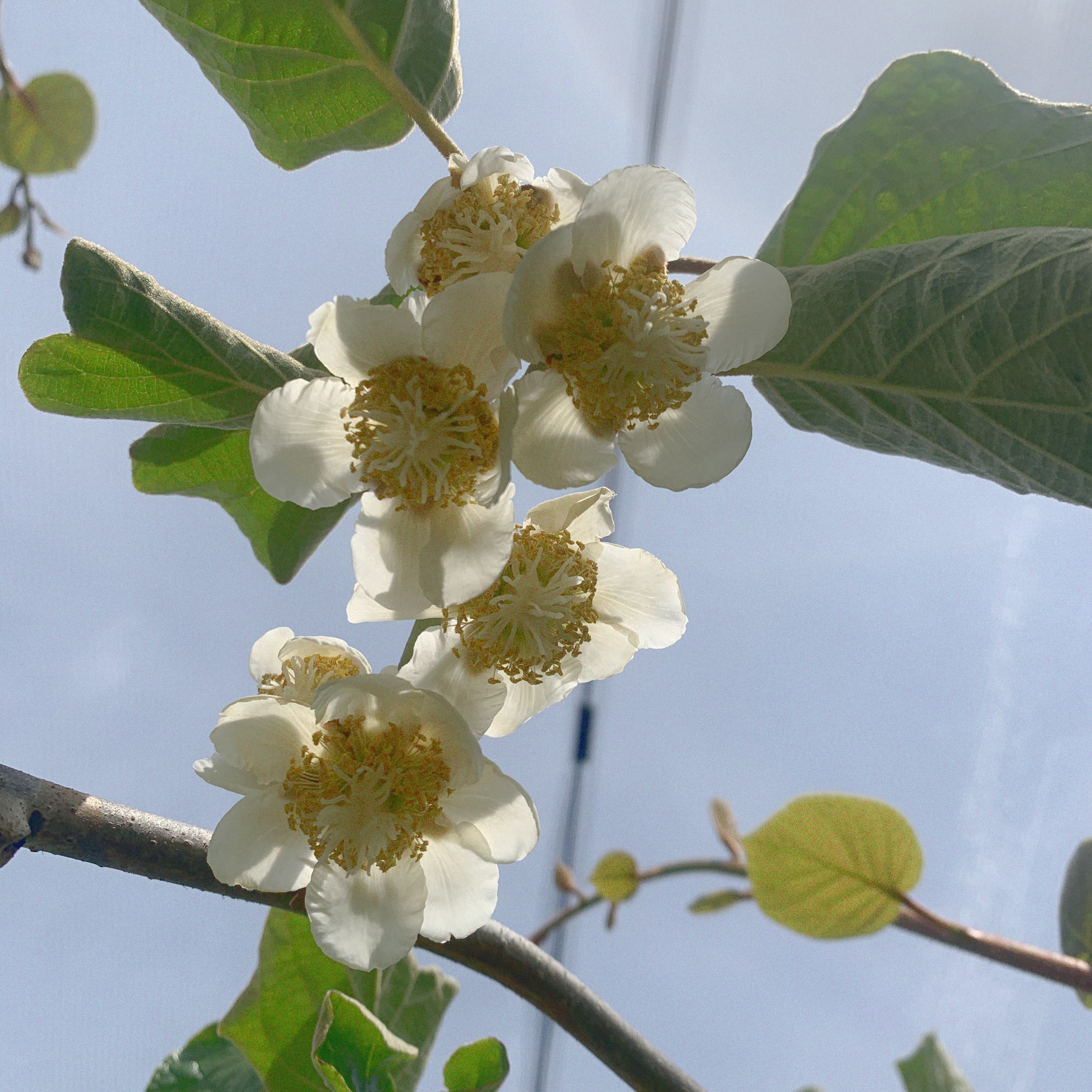

Supplement: Supplementary file 1 — Supplementary Information 1. [file 41598_2024_73035_MOESM1_ESM.zip › images/iso_noise__female.jpg]

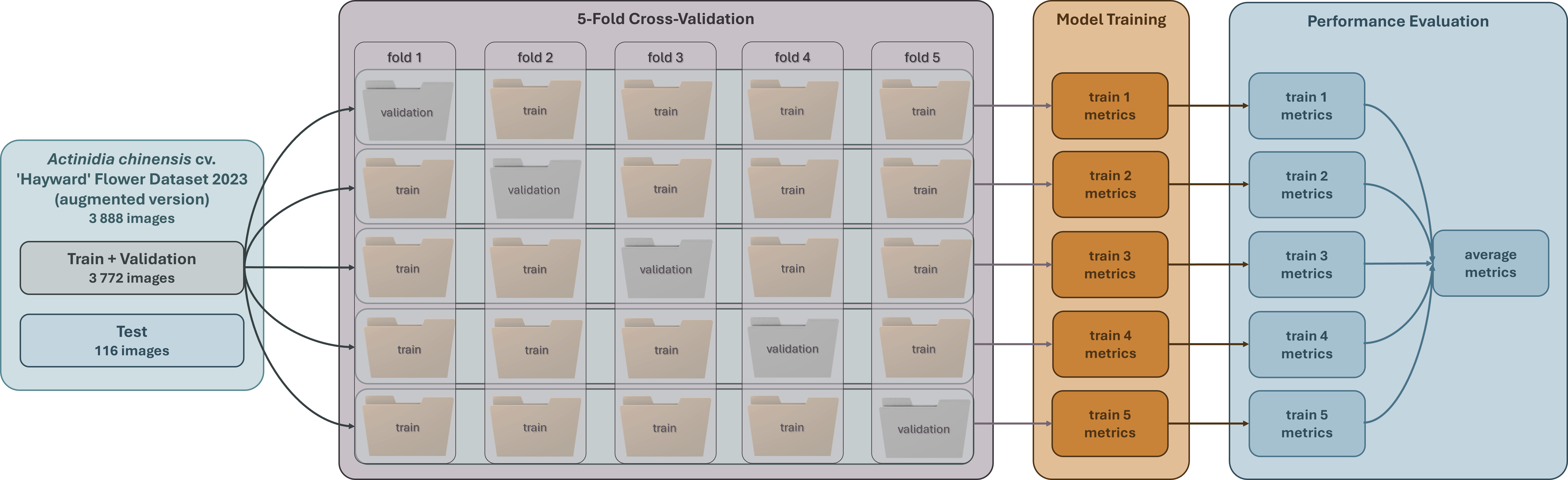

Supplement: Supplementary file 1 — Supplementary Information 1. [file 41598_2024_73035_MOESM1_ESM.zip › images/kfold.png]

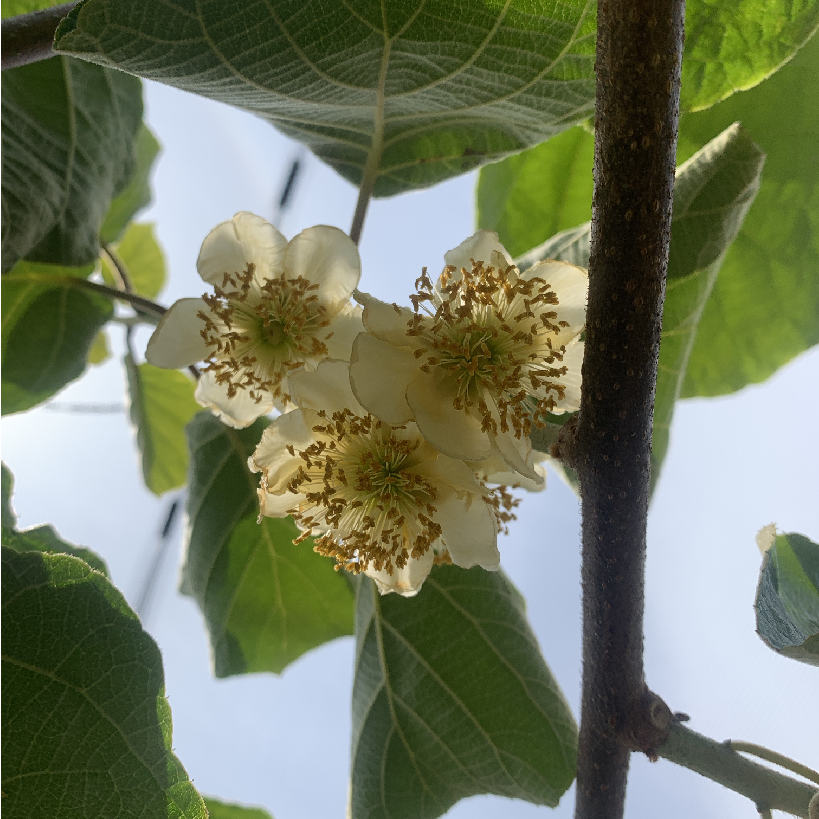

Supplement: Supplementary file 1 — Supplementary Information 1. [file 41598_2024_73035_MOESM1_ESM.zip › images/male.png]

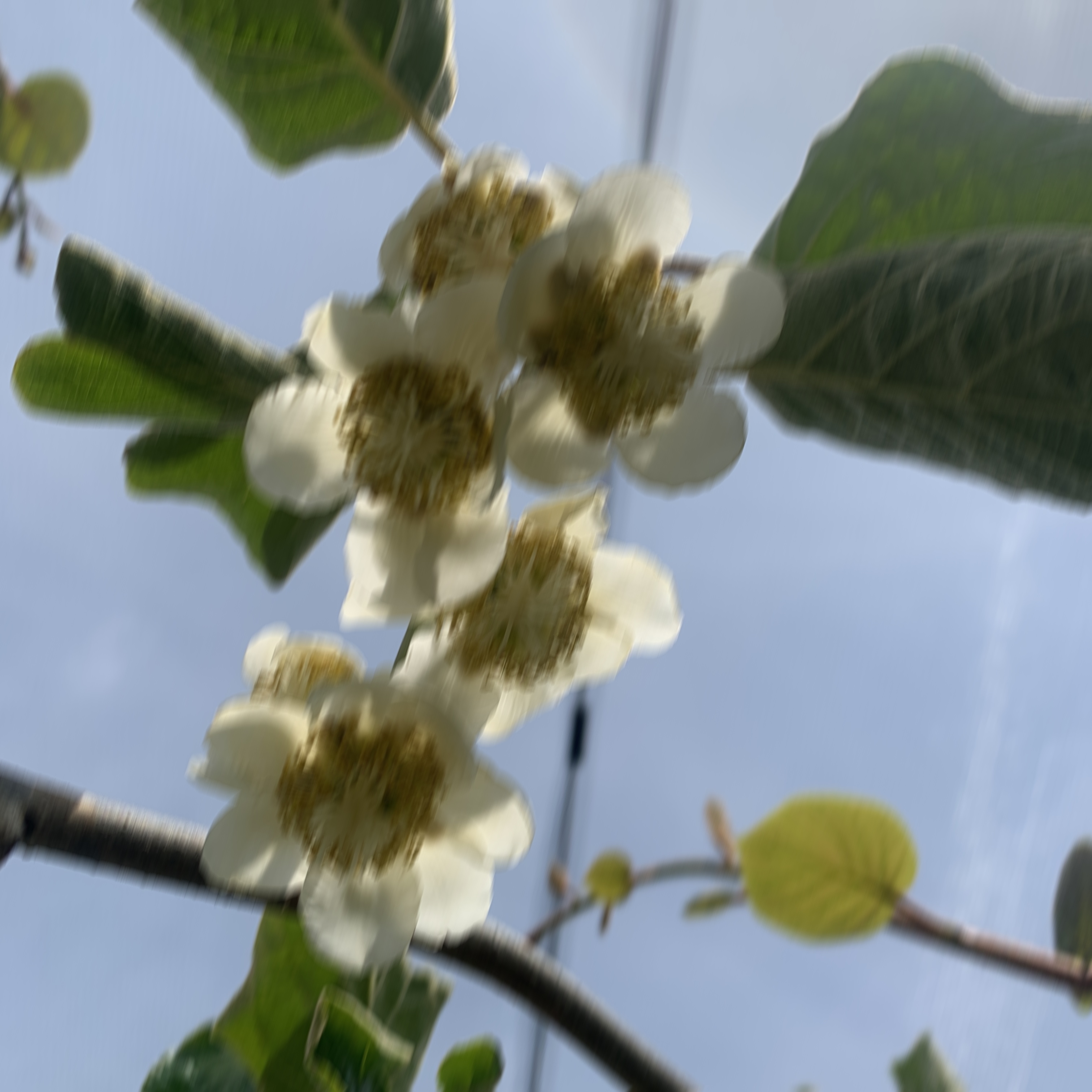

Supplement: Supplementary file 1 — Supplementary Information 1. [file 41598_2024_73035_MOESM1_ESM.zip › images/motion_blur__female.jpg]

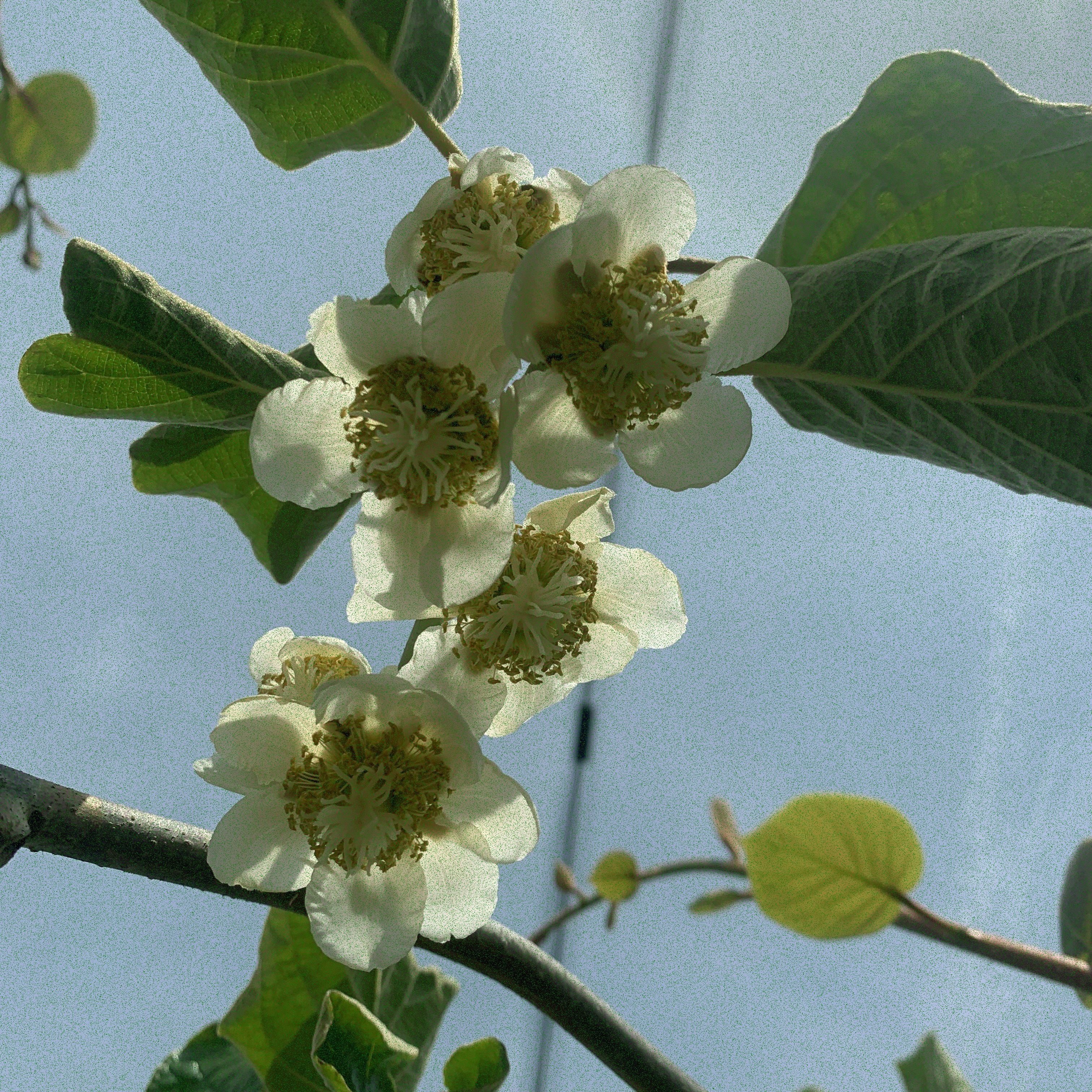

Supplement: Supplementary file 1 — Supplementary Information 1. [file 41598_2024_73035_MOESM1_ESM.zip › images/pixel_dropout__female.jpg]

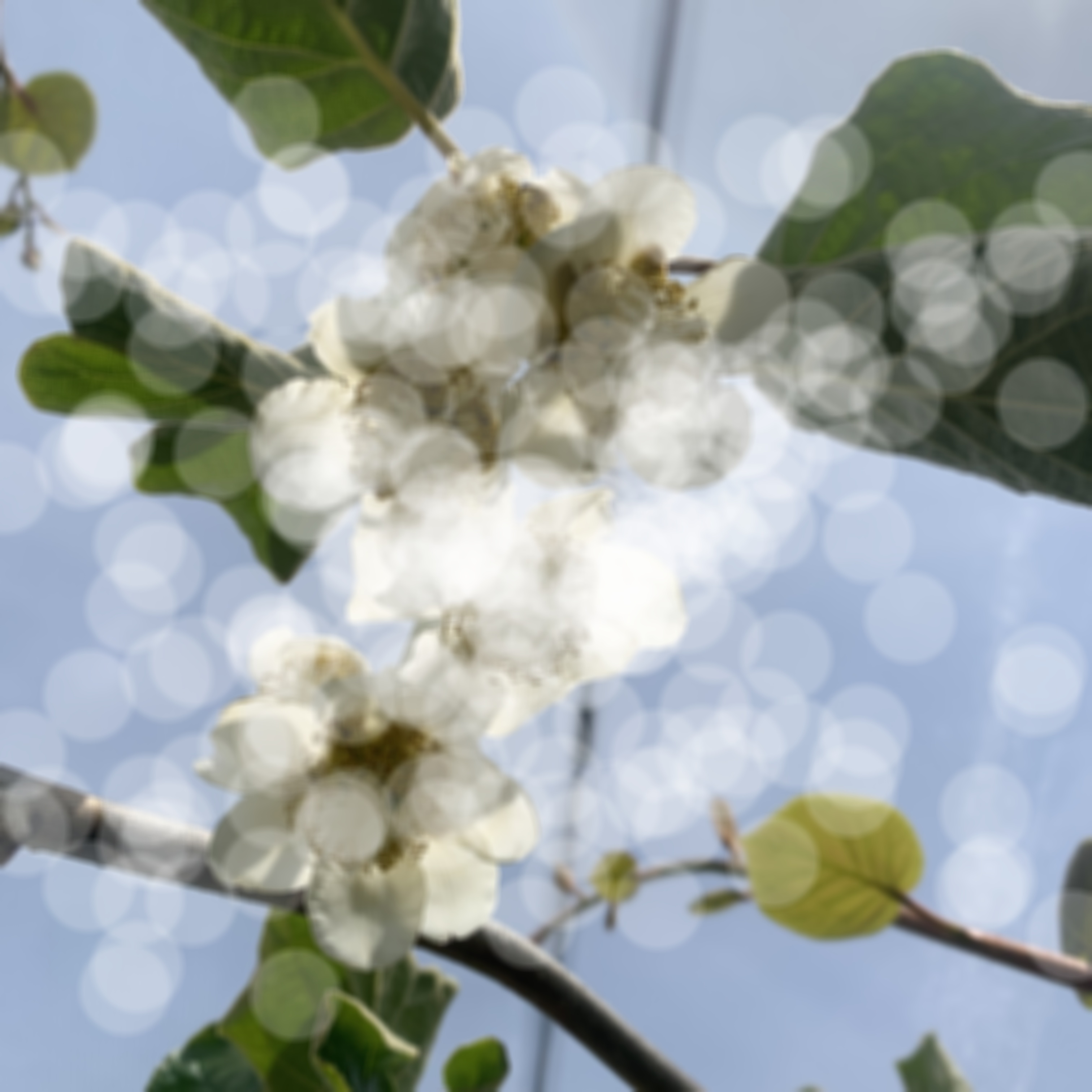

Supplement: Supplementary file 1 — Supplementary Information 1. [file 41598_2024_73035_MOESM1_ESM.zip › images/random_fog__female.jpg]

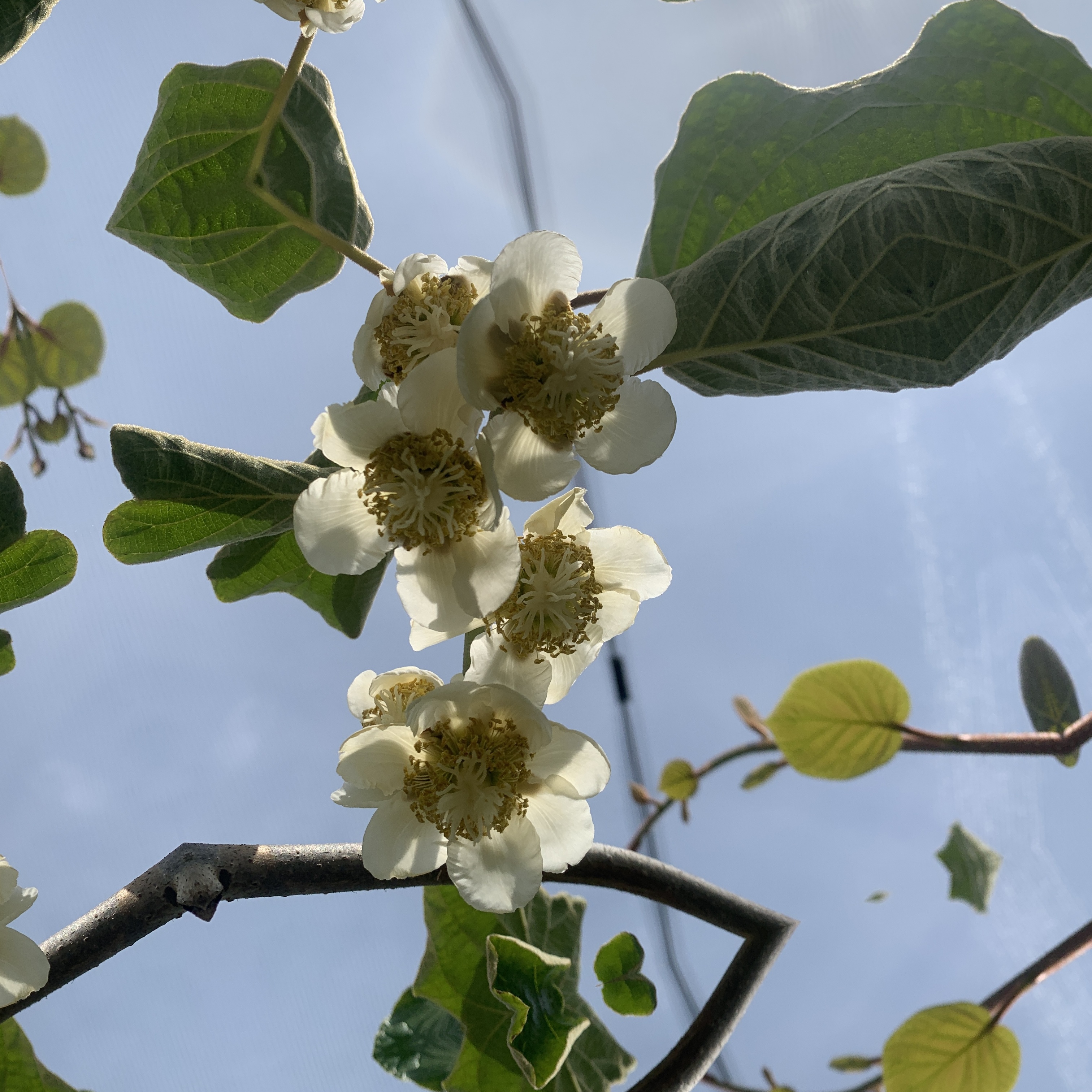

Supplement: Supplementary file 1 — Supplementary Information 1. [file 41598_2024_73035_MOESM1_ESM.zip › images/rotate__female.jpg]

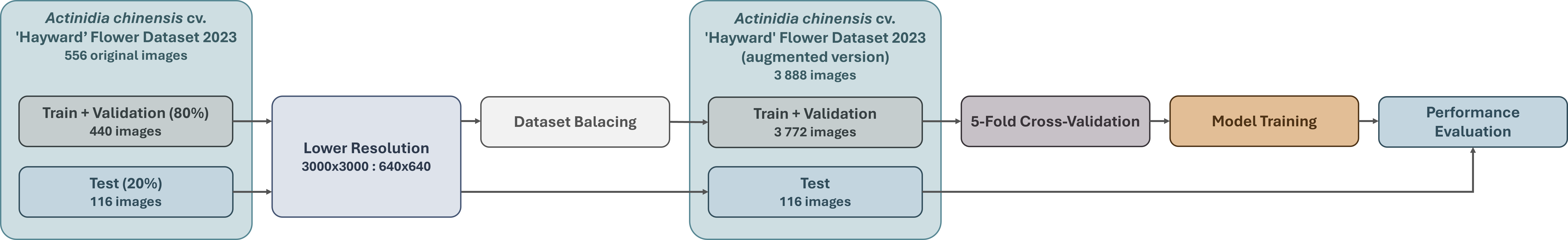

Supplement: Supplementary file 1 — Supplementary Information 1. [file 41598_2024_73035_MOESM1_ESM.zip › images/flow.png]

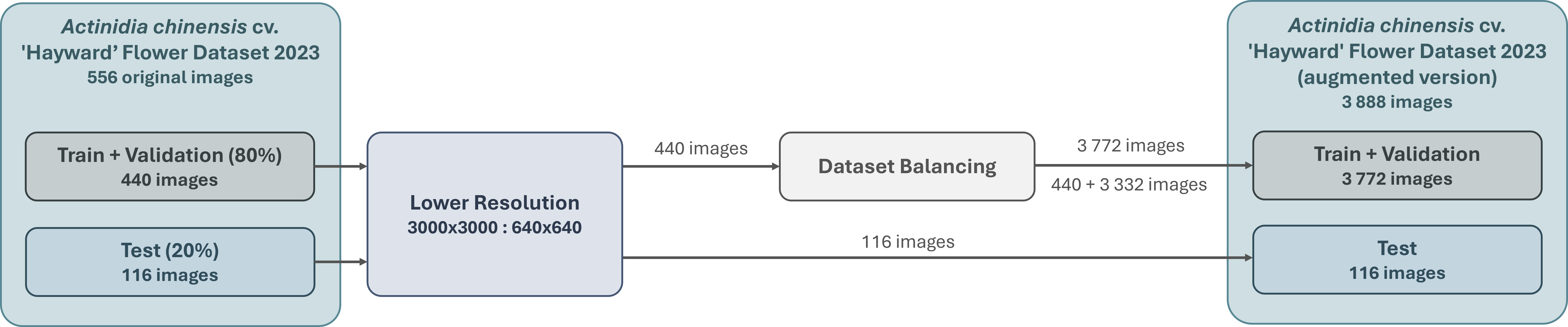

Supplement: Supplementary file 1 — Supplementary Information 1. [file 41598_2024_73035_MOESM1_ESM.zip › images/datasets.png]

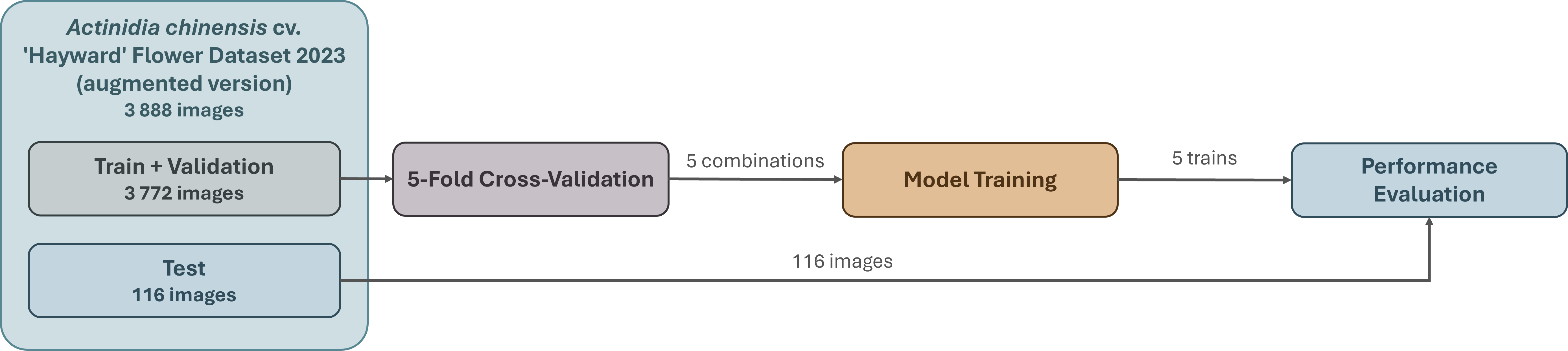

Supplement: Supplementary file 1 — Supplementary Information 1. [file 41598_2024_73035_MOESM1_ESM.zip › images/kfoldsimple.png]

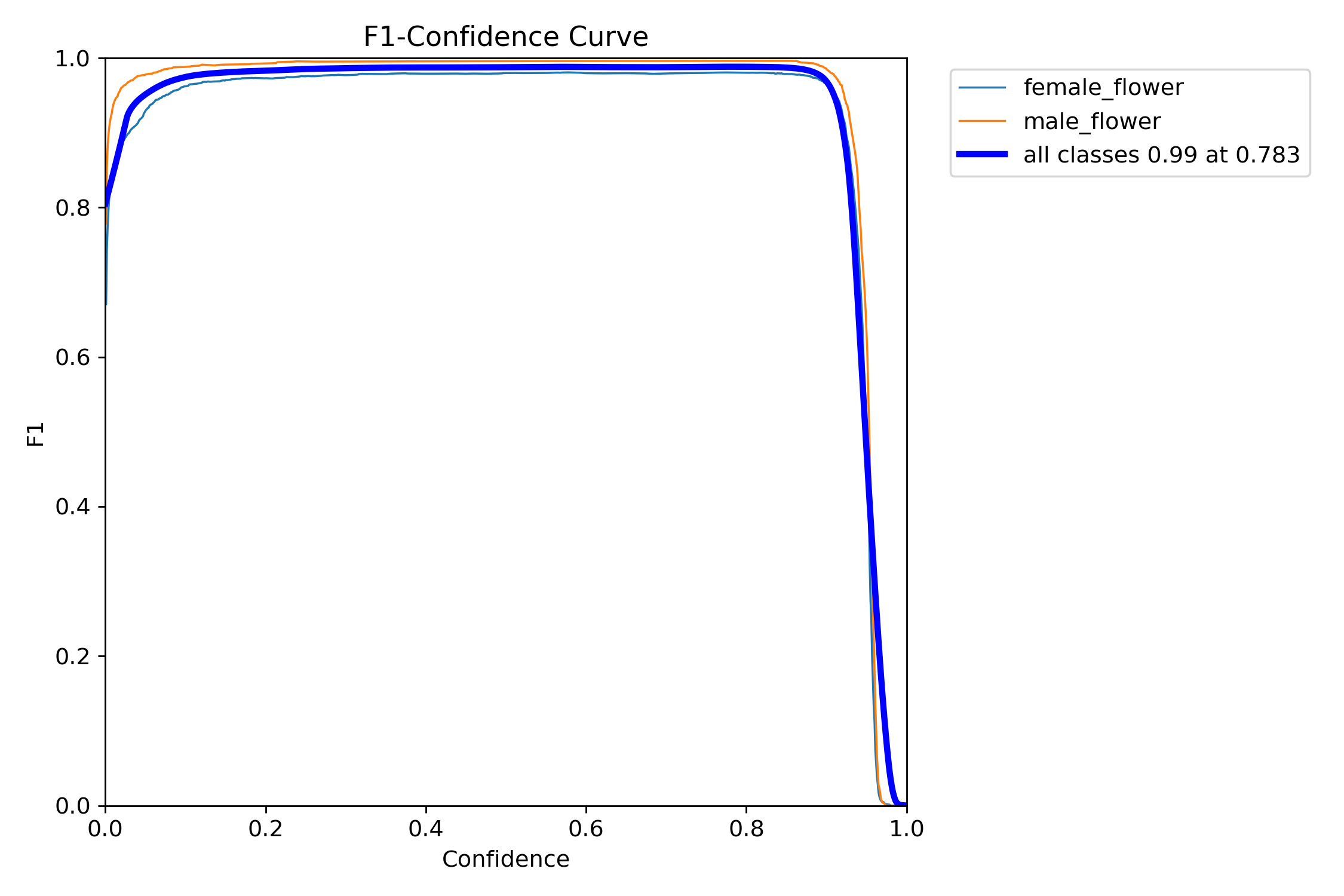

Supplement: Supplementary file 1 — Supplementary Information 1. [file 41598_2024_73035_MOESM1_ESM.zip › images/F1_curve.png]

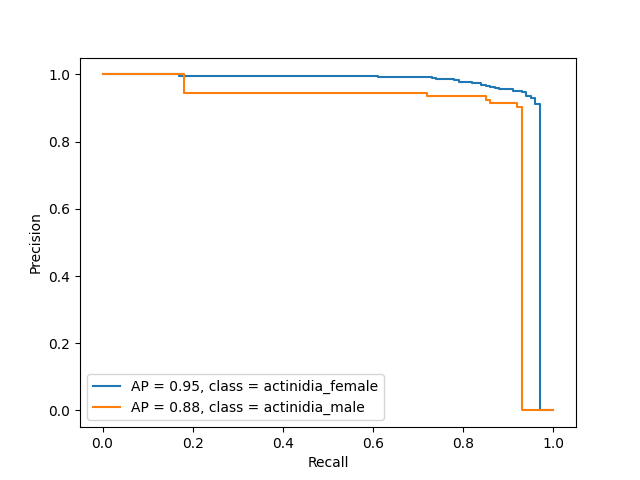

Supplement: Supplementary file 1 — Supplementary Information 1. [file 41598_2024_73035_MOESM1_ESM.zip › images/detr_split1.png]

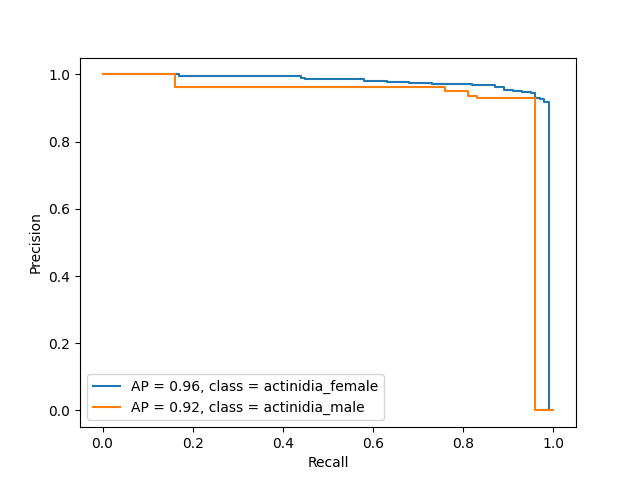

Supplement: Supplementary file 1 — Supplementary Information 1. [file 41598_2024_73035_MOESM1_ESM.zip › images/detr_split2.png]

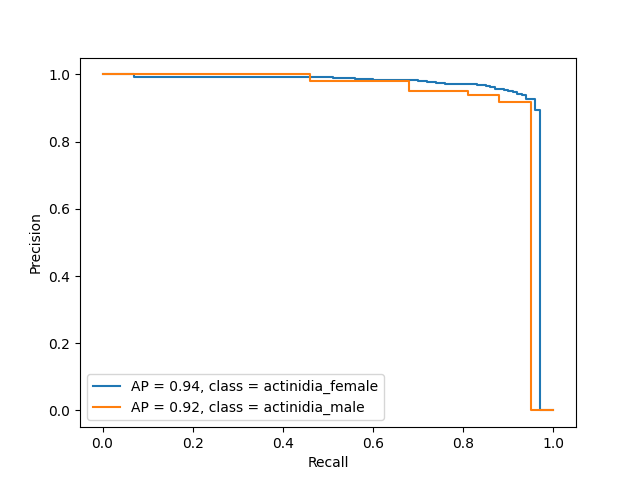

Supplement: Supplementary file 1 — Supplementary Information 1. [file 41598_2024_73035_MOESM1_ESM.zip › images/detr_split3.png]

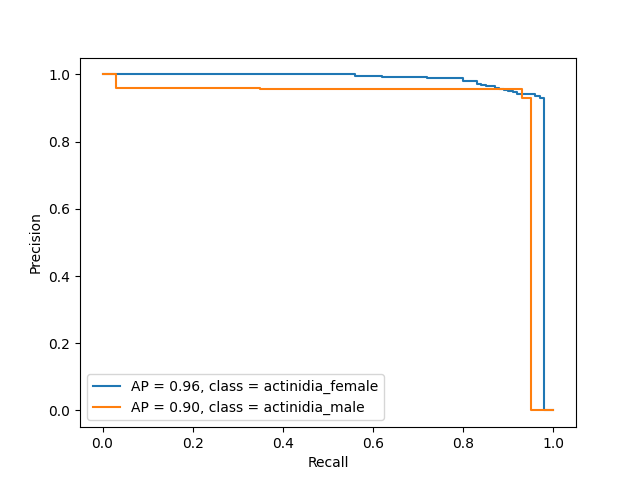

Supplement: Supplementary file 1 — Supplementary Information 1. [file 41598_2024_73035_MOESM1_ESM.zip › images/detr_split4.png]

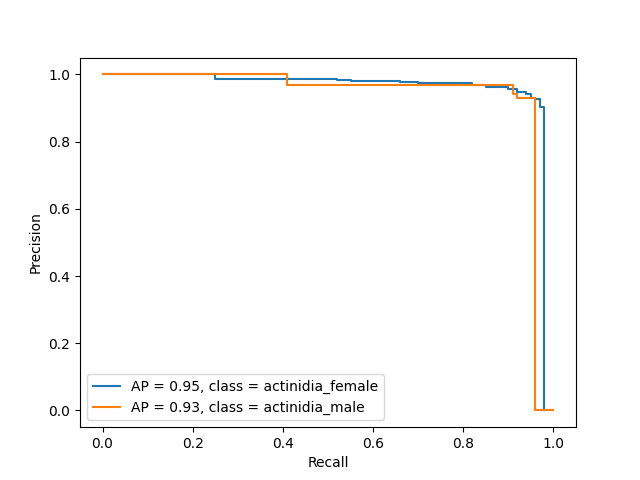

Supplement: Supplementary file 1 — Supplementary Information 1. [file 41598_2024_73035_MOESM1_ESM.zip › images/detr_split5.png]

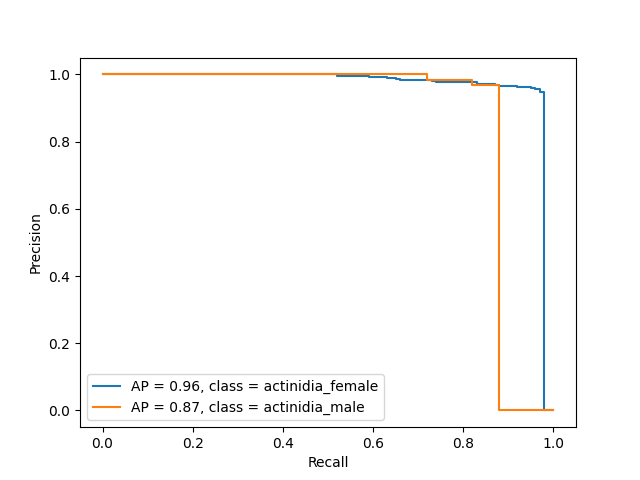

Supplement: Supplementary file 1 — Supplementary Information 1. [file 41598_2024_73035_MOESM1_ESM.zip › images/rtdetr_split1.png]

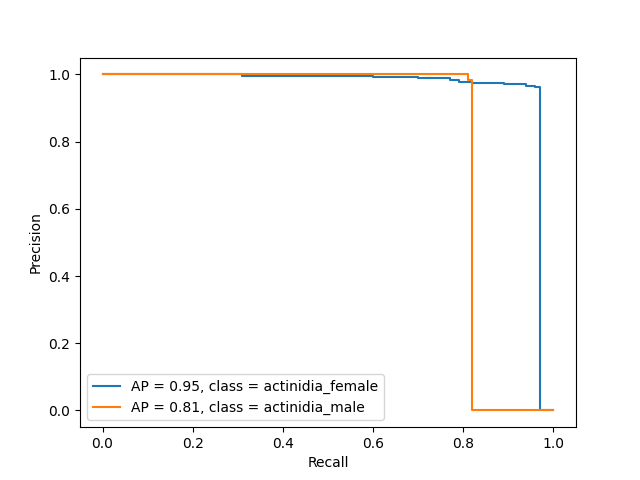

Supplement: Supplementary file 1 — Supplementary Information 1. [file 41598_2024_73035_MOESM1_ESM.zip › images/rtdetr_split2.png]

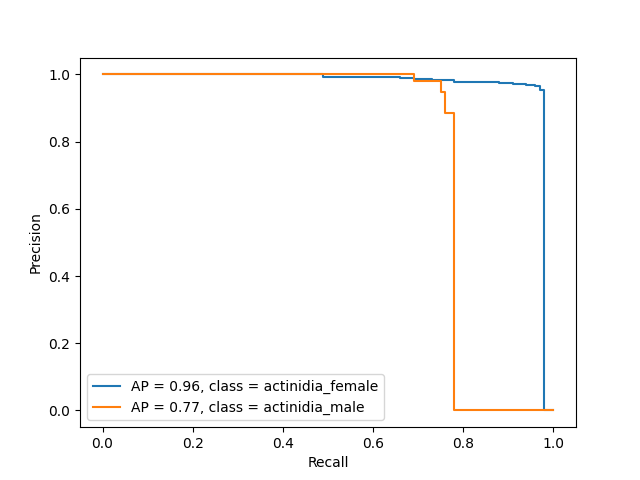

Supplement: Supplementary file 1 — Supplementary Information 1. [file 41598_2024_73035_MOESM1_ESM.zip › images/rtdetr_split3.png]

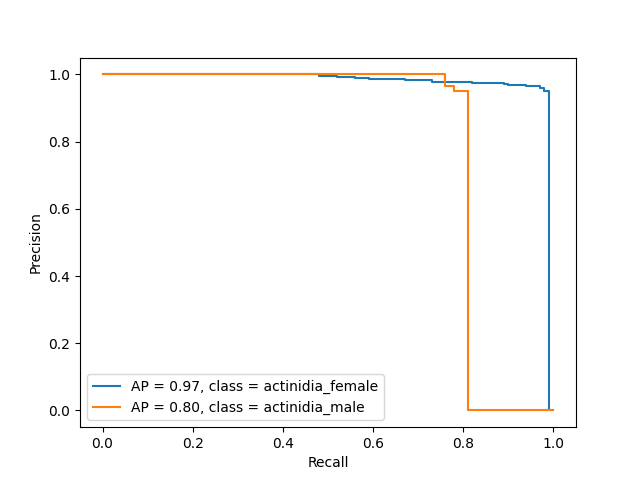

Supplement: Supplementary file 1 — Supplementary Information 1. [file 41598_2024_73035_MOESM1_ESM.zip › images/rtdetr_split4.png]

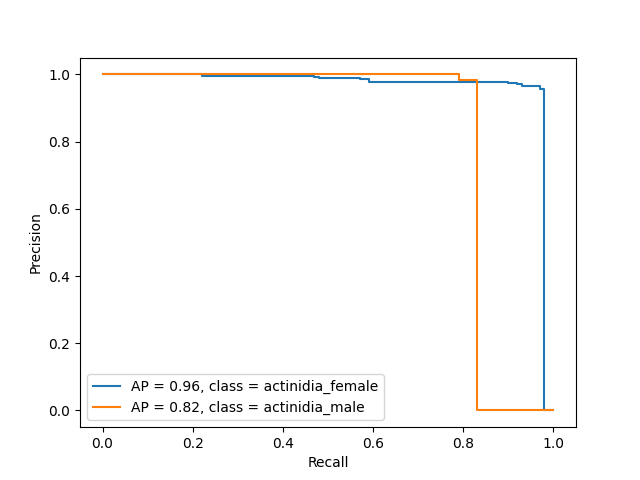

Supplement: Supplementary file 1 — Supplementary Information 1. [file 41598_2024_73035_MOESM1_ESM.zip › images/rtdetr_split5.png]

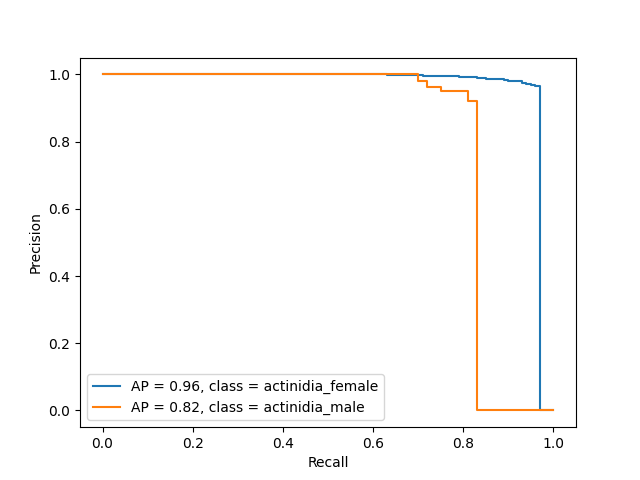

Supplement: Supplementary file 1 — Supplementary Information 1. [file 41598_2024_73035_MOESM1_ESM.zip › images/yolov5_split1.png]

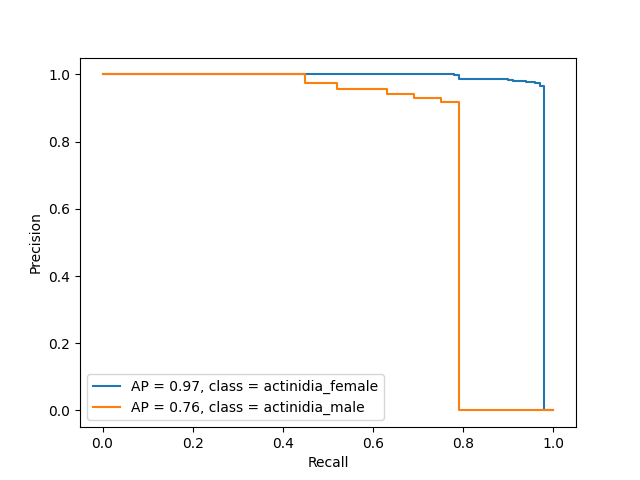

Supplement: Supplementary file 1 — Supplementary Information 1. [file 41598_2024_73035_MOESM1_ESM.zip › images/yolov5_split2.png]

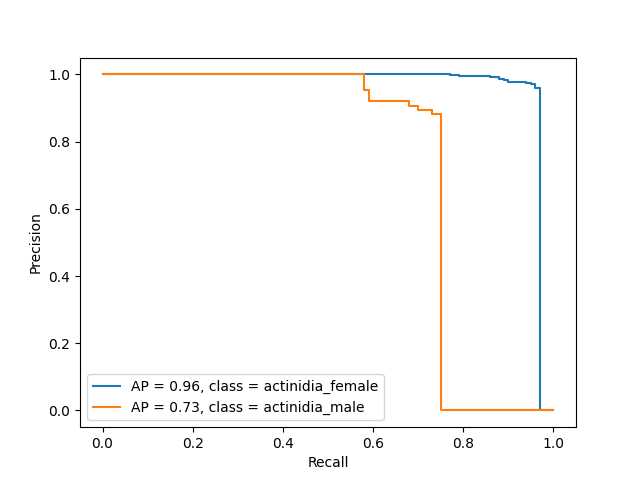

Supplement: Supplementary file 1 — Supplementary Information 1. [file 41598_2024_73035_MOESM1_ESM.zip › images/yolov5_split3.png]

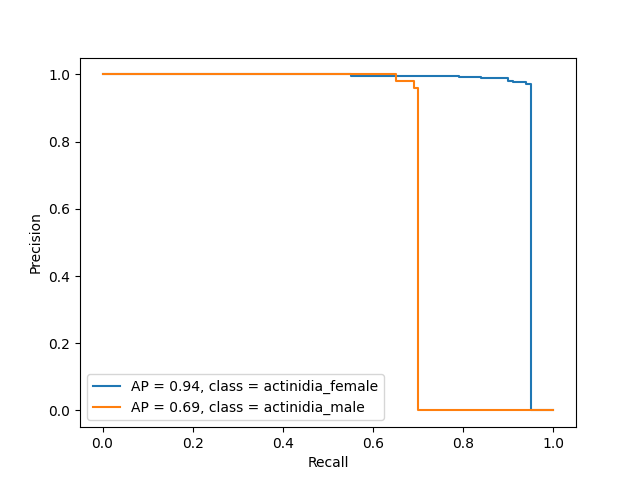

Supplement: Supplementary file 1 — Supplementary Information 1. [file 41598_2024_73035_MOESM1_ESM.zip › images/yolov5_split4.png]

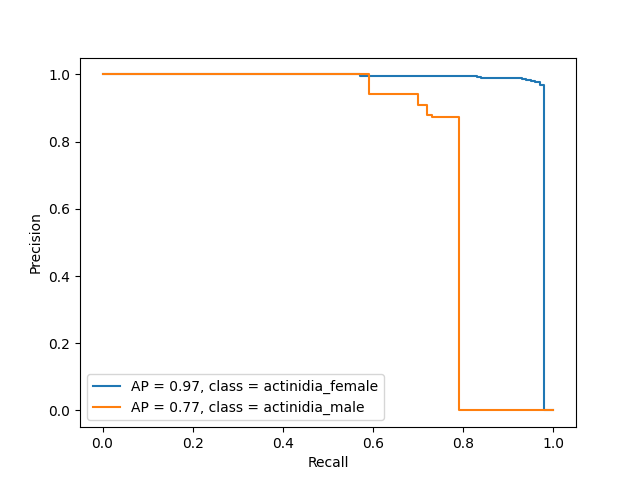

Supplement: Supplementary file 1 — Supplementary Information 1. [file 41598_2024_73035_MOESM1_ESM.zip › images/yolov5_split5.png]

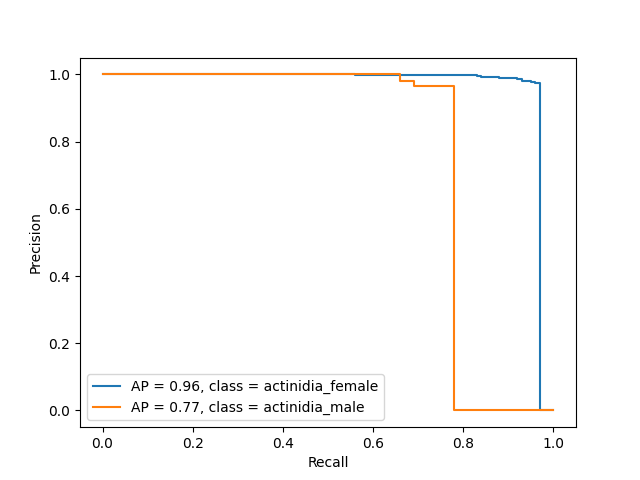

Supplement: Supplementary file 1 — Supplementary Information 1. [file 41598_2024_73035_MOESM1_ESM.zip › images/yolov8_split1.png]

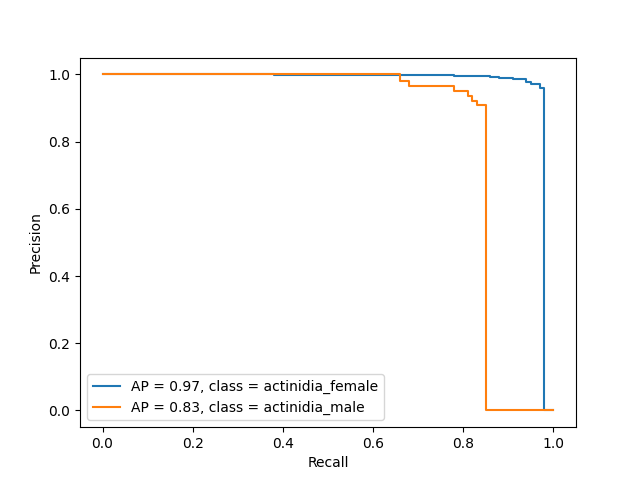

Supplement: Supplementary file 1 — Supplementary Information 1. [file 41598_2024_73035_MOESM1_ESM.zip › images/yolov8_split2.png]

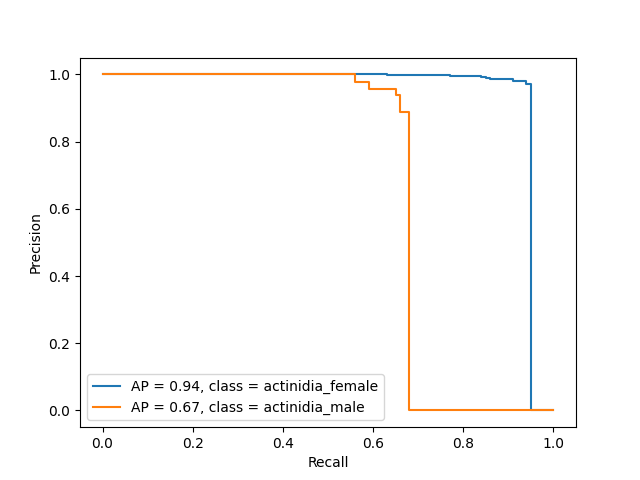

Supplement: Supplementary file 1 — Supplementary Information 1. [file 41598_2024_73035_MOESM1_ESM.zip › images/yolov8_split3.png]

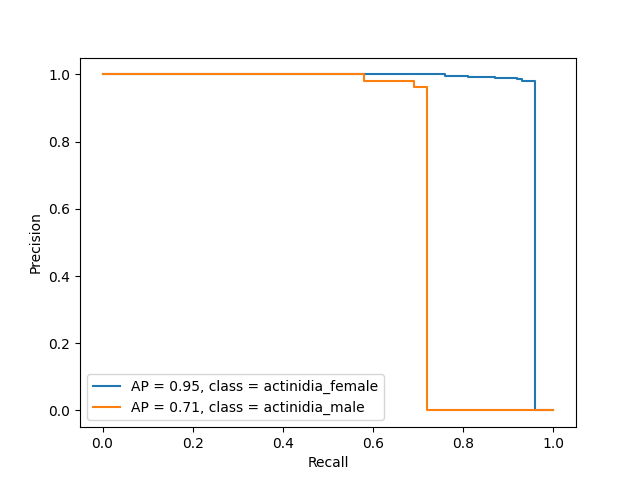

Supplement: Supplementary file 1 — Supplementary Information 1. [file 41598_2024_73035_MOESM1_ESM.zip › images/yolov8_split4.png]

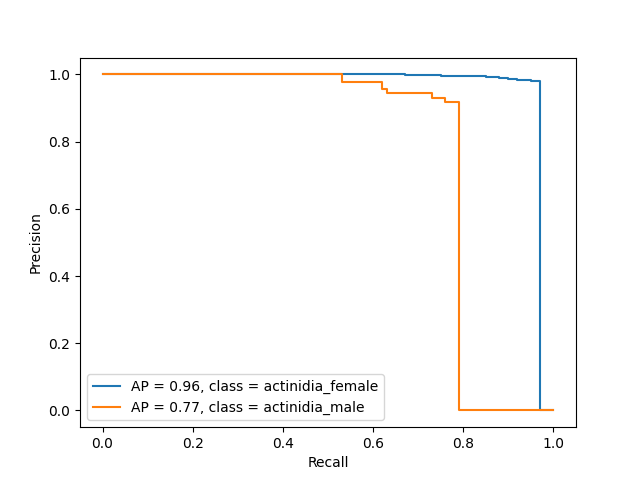

Supplement: Supplementary file 1 — Supplementary Information 1. [file 41598_2024_73035_MOESM1_ESM.zip › images/yolov8_split5.png]

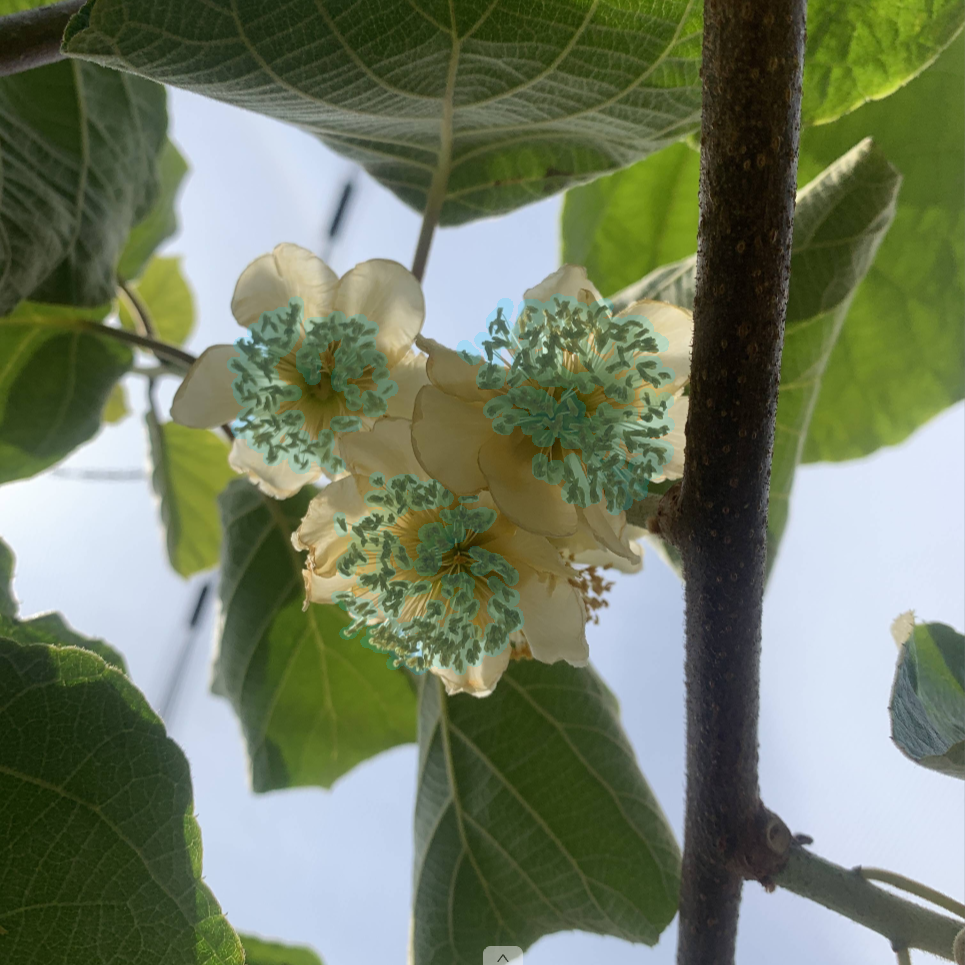

Supplement: Supplementary file 1 — Supplementary Information 1. [file 41598_2024_73035_MOESM1_ESM.zip › images/male_legend.png]

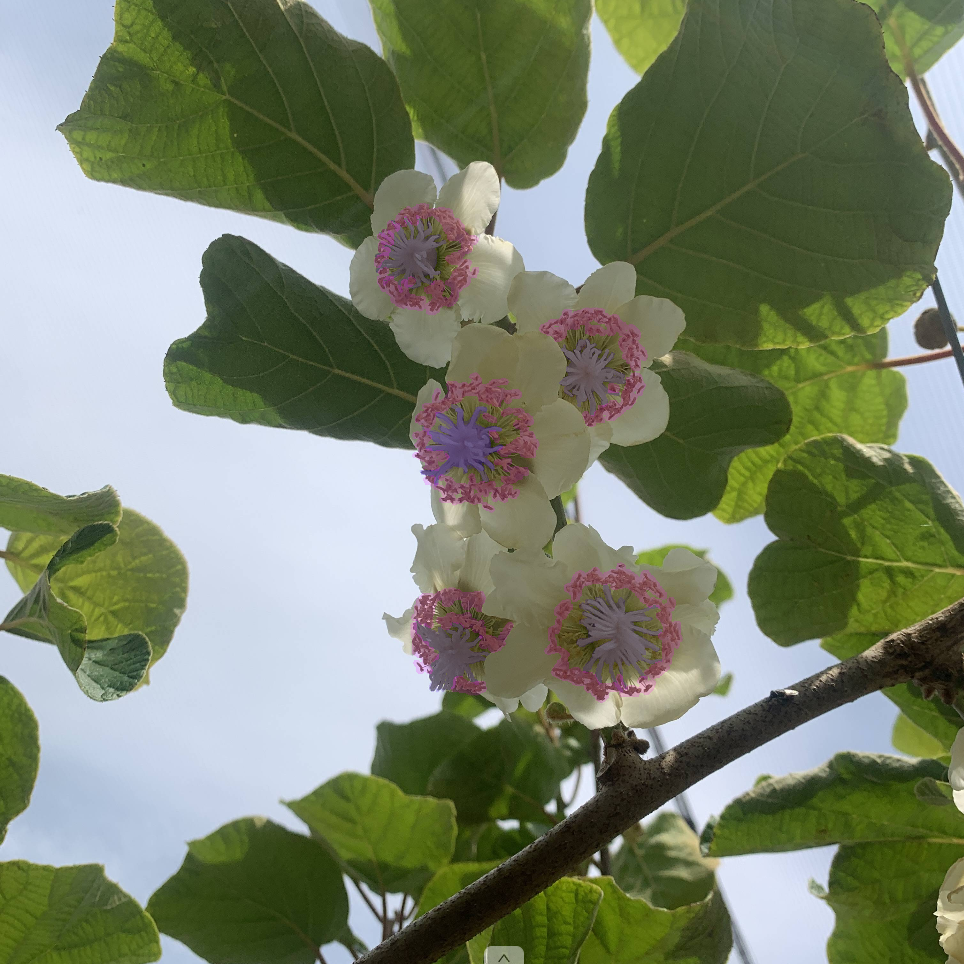

Supplement: Supplementary file 1 — Supplementary Information 1. [file 41598_2024_73035_MOESM1_ESM.zip › images/female_legend.png]

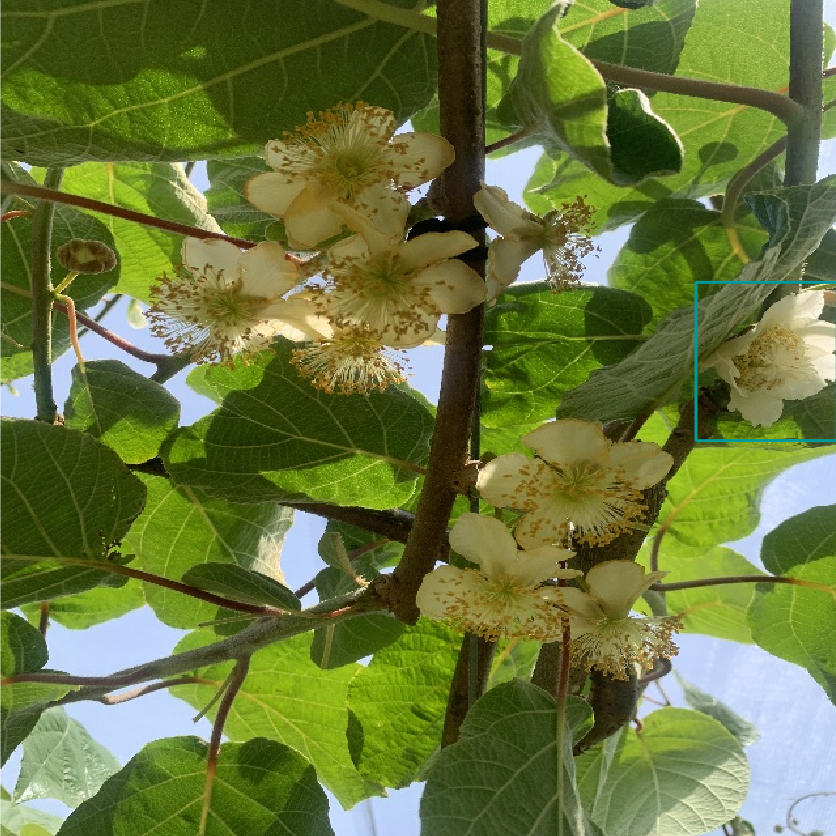

Supplement: Supplementary file 1 — Supplementary Information 1. [file 41598_2024_73035_MOESM1_ESM.zip › images/detr_female_fn1.png]

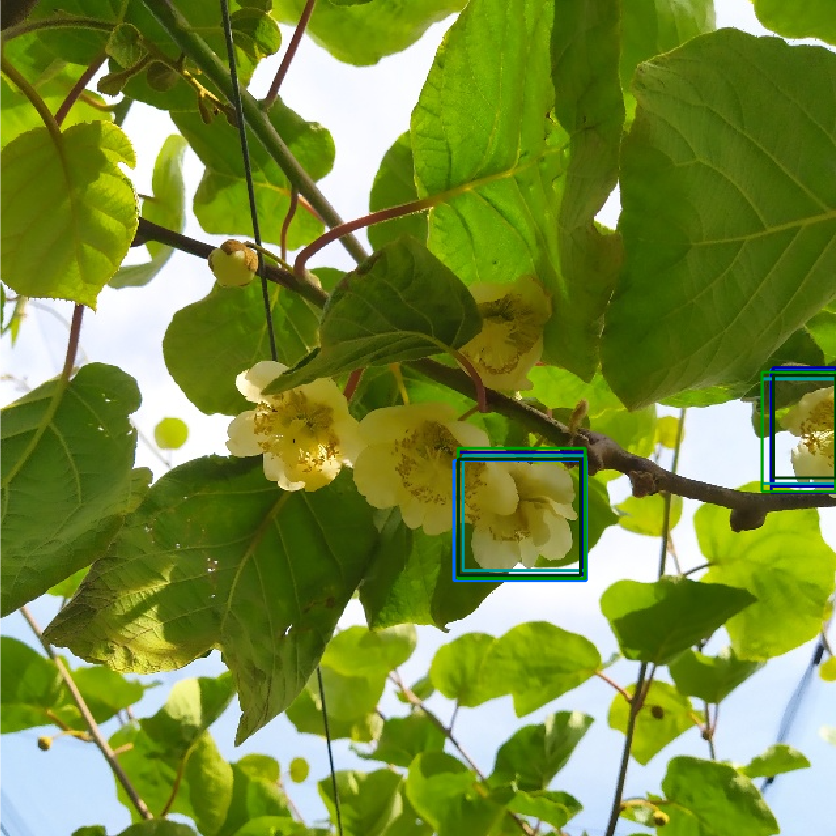

Supplement: Supplementary file 1 — Supplementary Information 1. [file 41598_2024_73035_MOESM1_ESM.zip › images/detr_female_fp1.png]

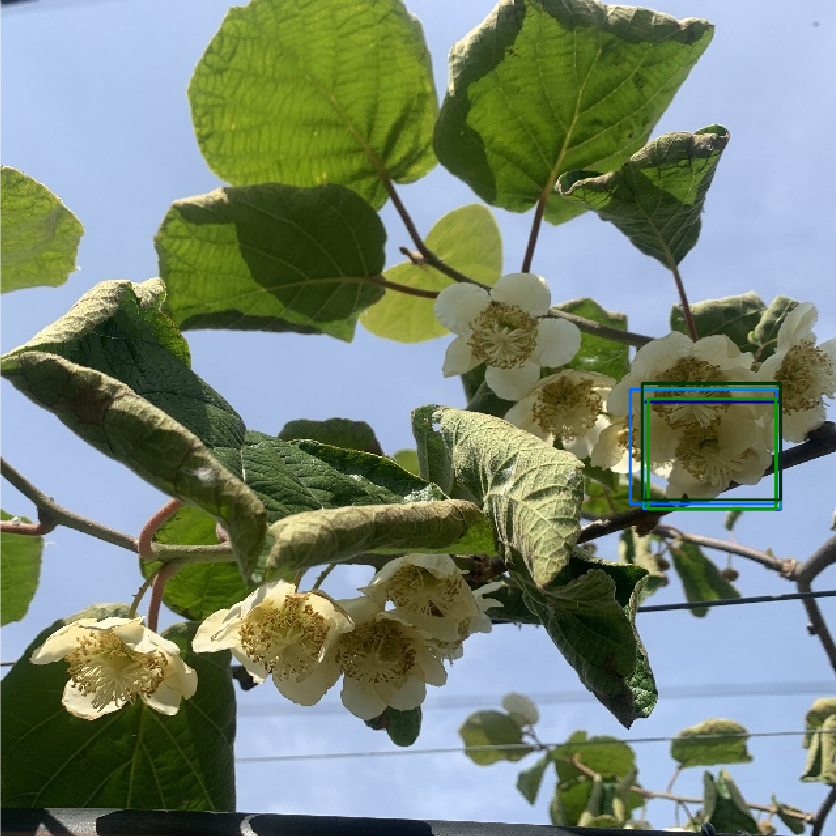

Supplement: Supplementary file 1 — Supplementary Information 1. [file 41598_2024_73035_MOESM1_ESM.zip › images/detr_female_fp2.png]

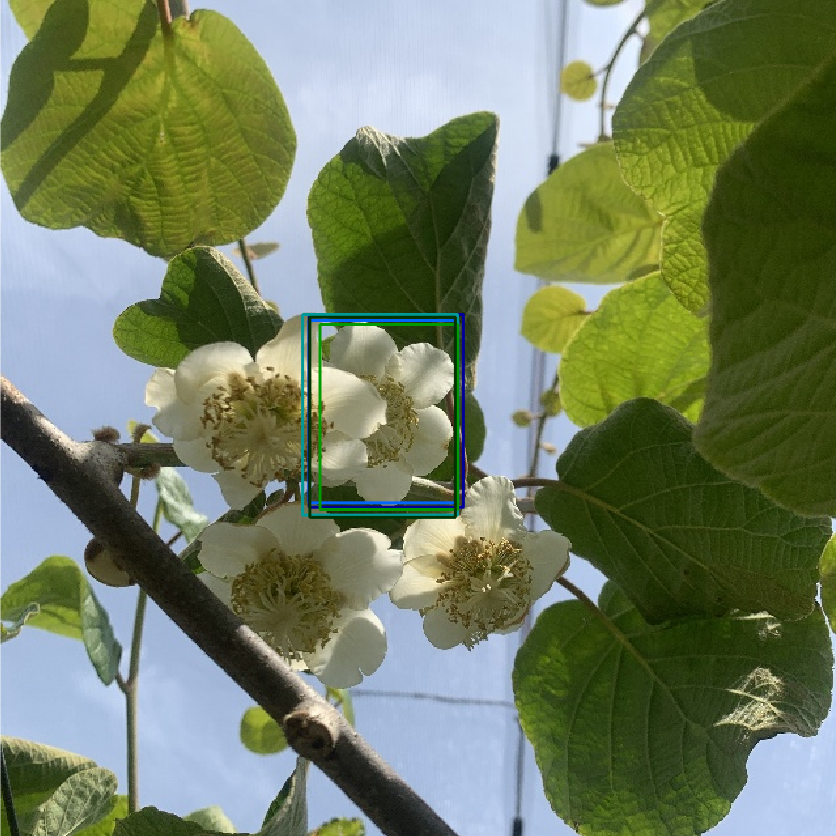

Supplement: Supplementary file 1 — Supplementary Information 1. [file 41598_2024_73035_MOESM1_ESM.zip › images/detr_female_fp3.png]

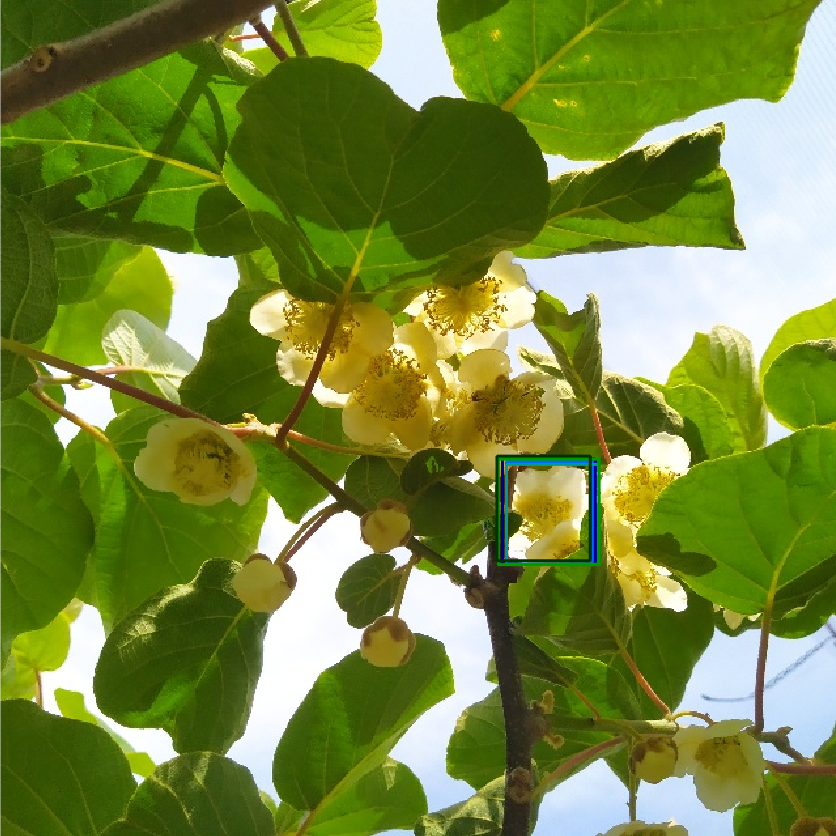

Supplement: Supplementary file 1 — Supplementary Information 1. [file 41598_2024_73035_MOESM1_ESM.zip › images/detr_female_fp4.png]

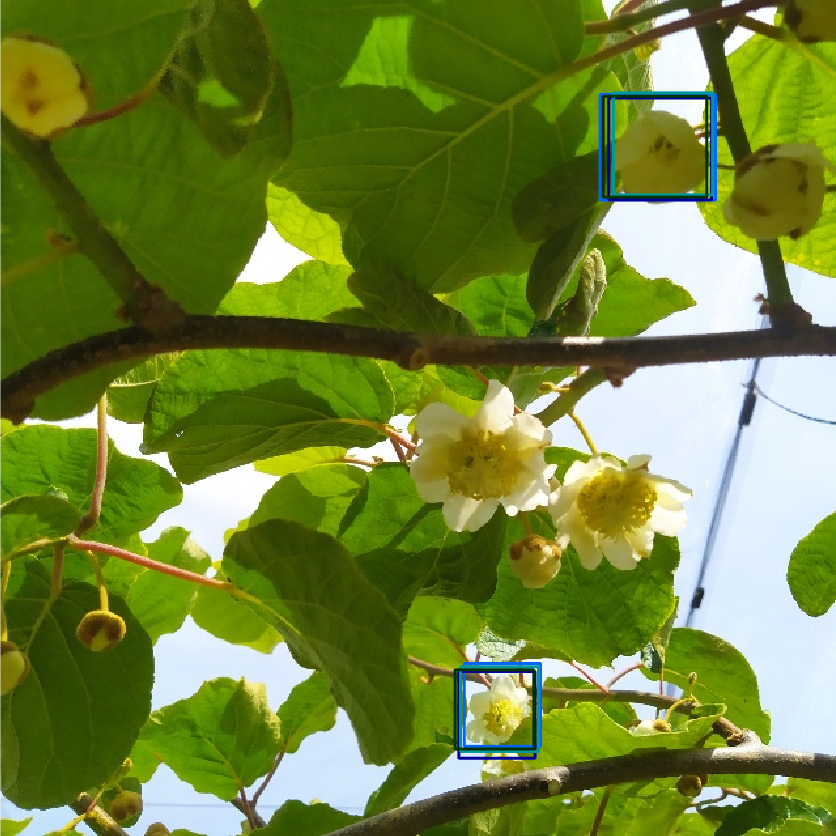

Supplement: Supplementary file 1 — Supplementary Information 1. [file 41598_2024_73035_MOESM1_ESM.zip › images/detr_female_fp5.png]

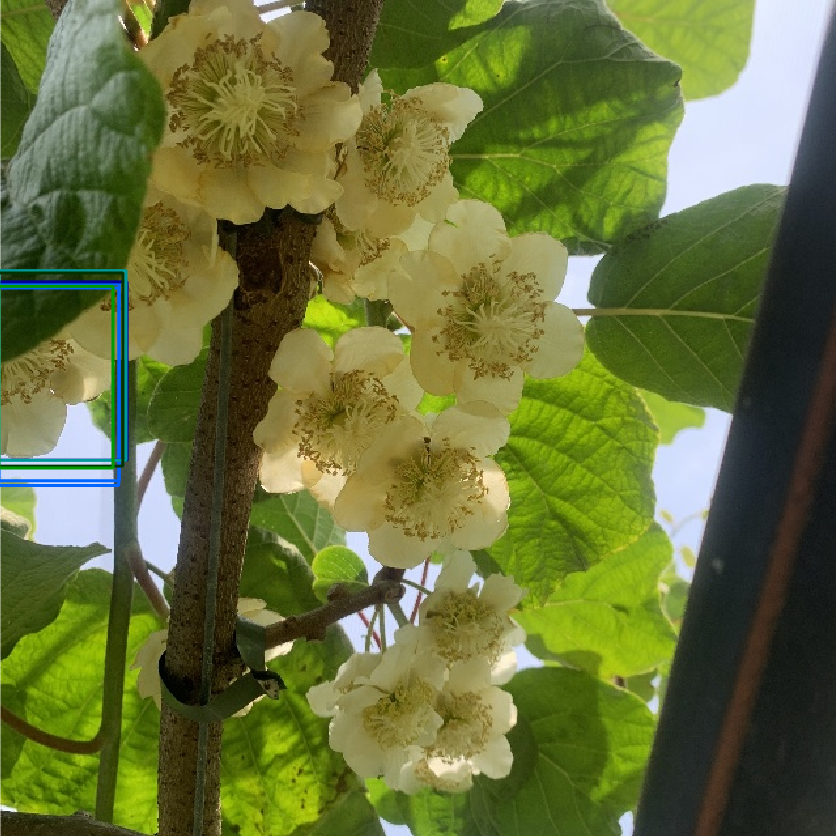

Supplement: Supplementary file 1 — Supplementary Information 1. [file 41598_2024_73035_MOESM1_ESM.zip › images/detr_female_fp6.png]

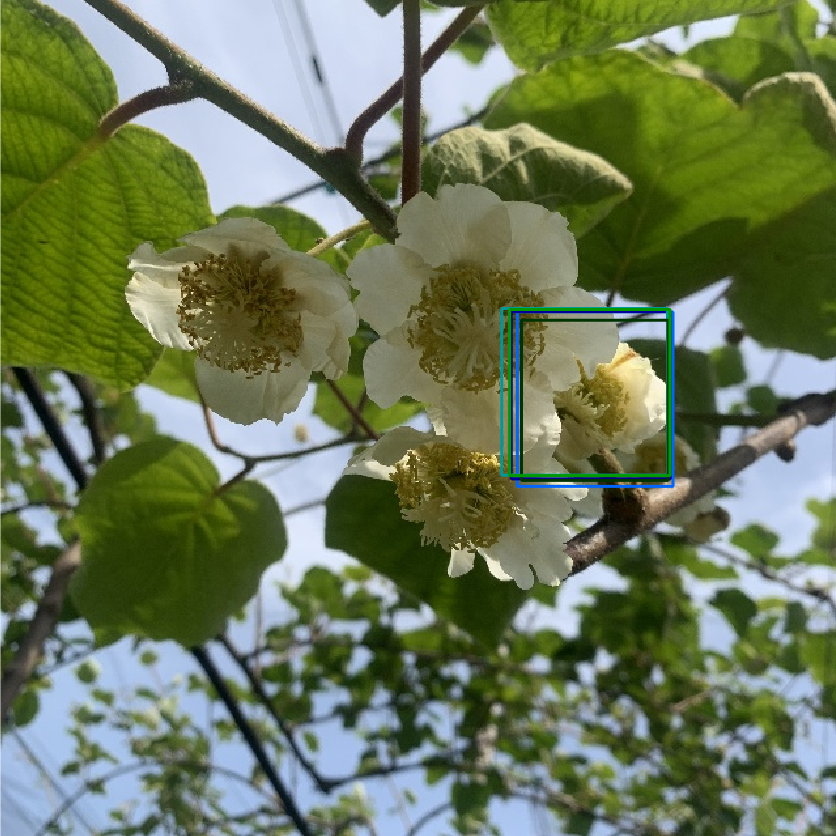

Supplement: Supplementary file 1 — Supplementary Information 1. [file 41598_2024_73035_MOESM1_ESM.zip › images/detr_female_fp7.png]

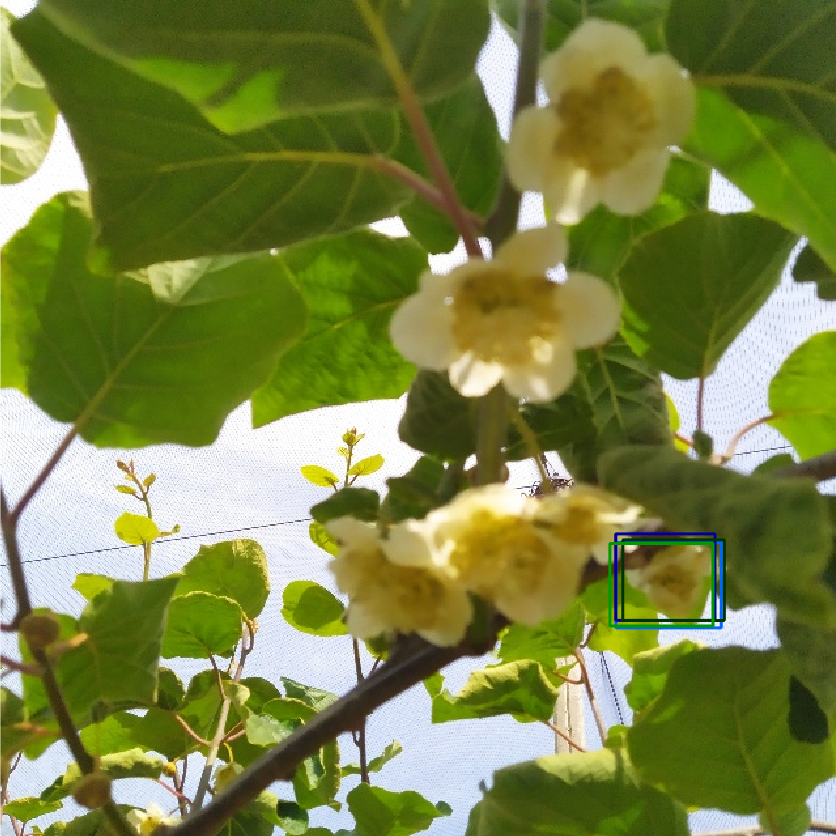

Supplement: Supplementary file 1 — Supplementary Information 1. [file 41598_2024_73035_MOESM1_ESM.zip › images/detr_female_fp8.png]

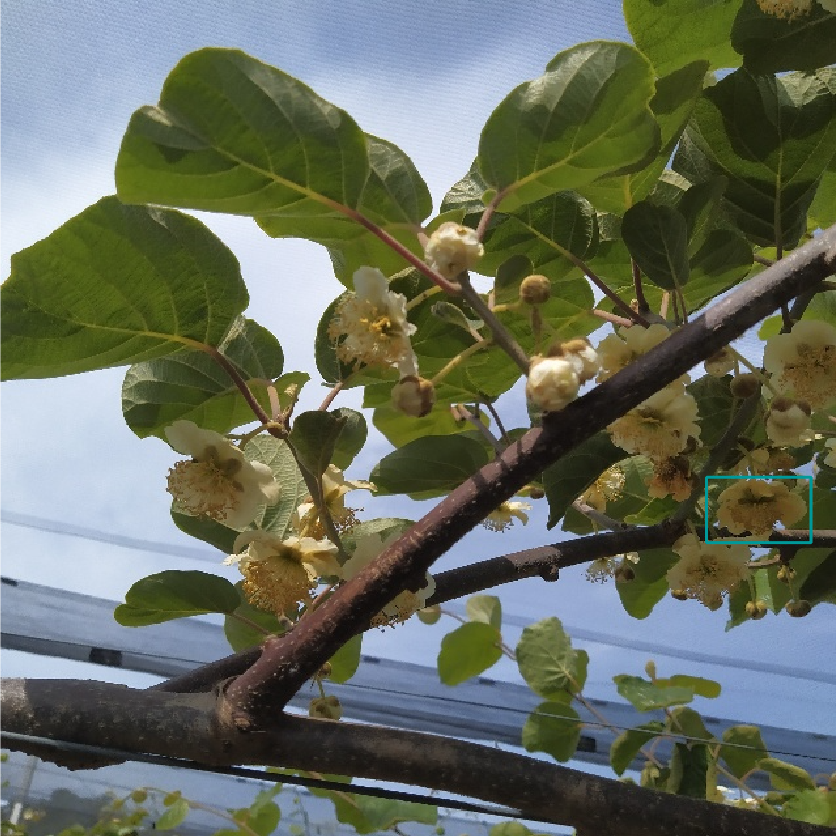

Supplement: Supplementary file 1 — Supplementary Information 1. [file 41598_2024_73035_MOESM1_ESM.zip › images/detr_male_fn1.png]

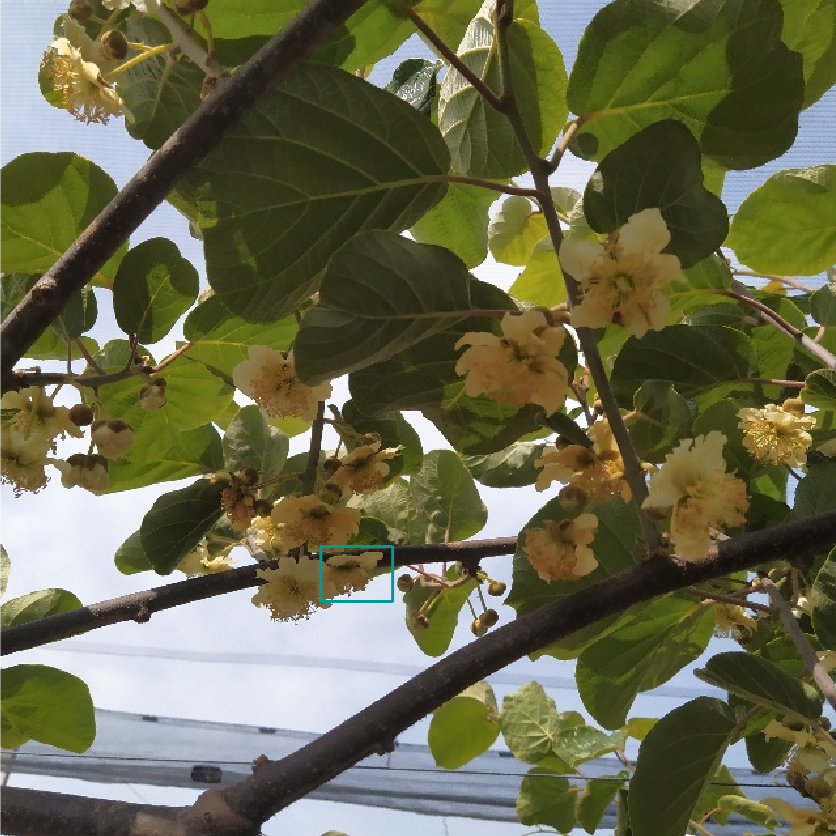

Supplement: Supplementary file 1 — Supplementary Information 1. [file 41598_2024_73035_MOESM1_ESM.zip › images/detr_male_fn2.png]

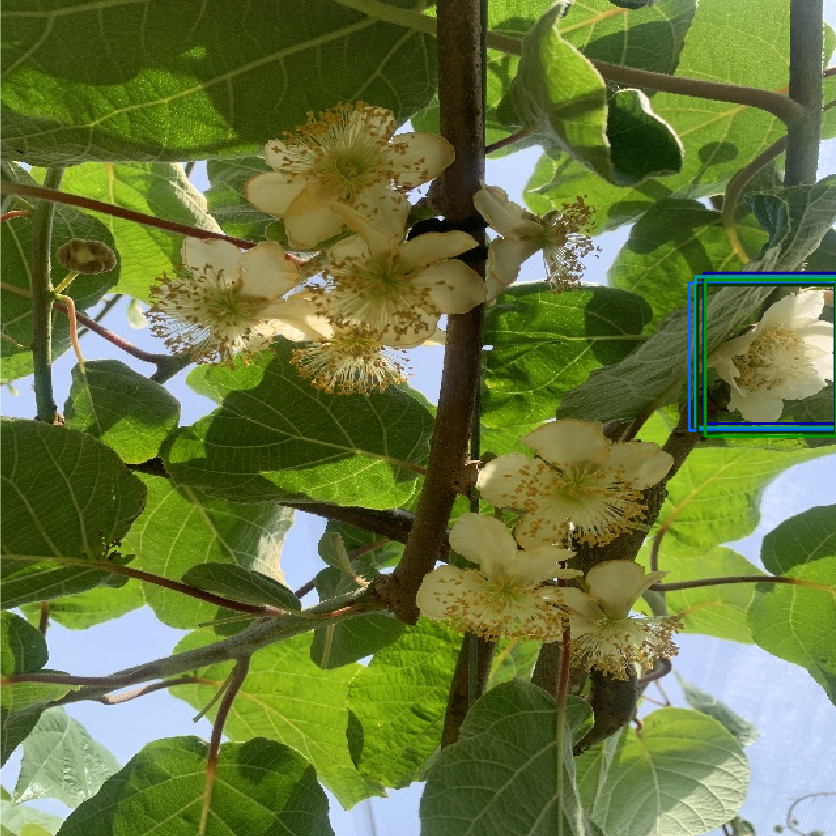

Supplement: Supplementary file 1 — Supplementary Information 1. [file 41598_2024_73035_MOESM1_ESM.zip › images/detr_male_fp1.png]

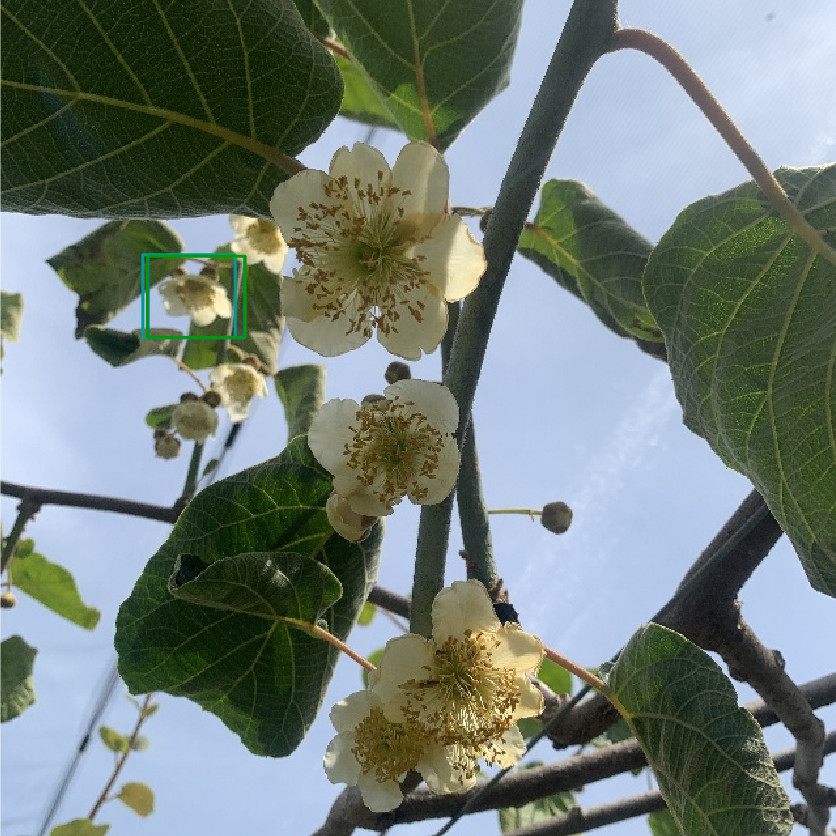

Supplement: Supplementary file 1 — Supplementary Information 1. [file 41598_2024_73035_MOESM1_ESM.zip › images/detr_male_fp2.png]

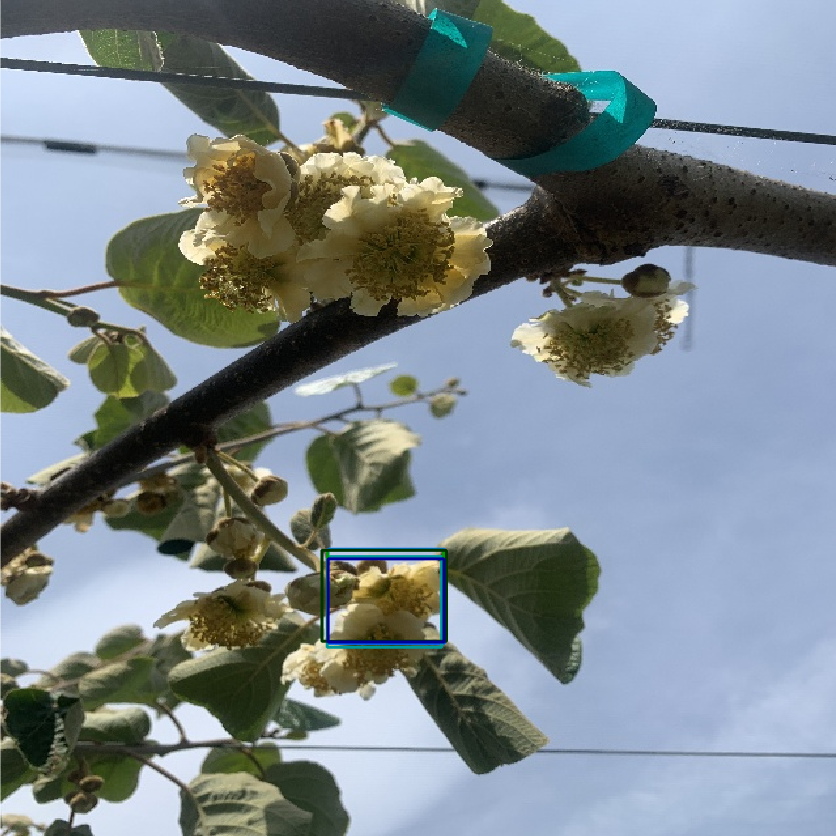

Supplement: Supplementary file 1 — Supplementary Information 1. [file 41598_2024_73035_MOESM1_ESM.zip › images/detr_male_fp3.png]

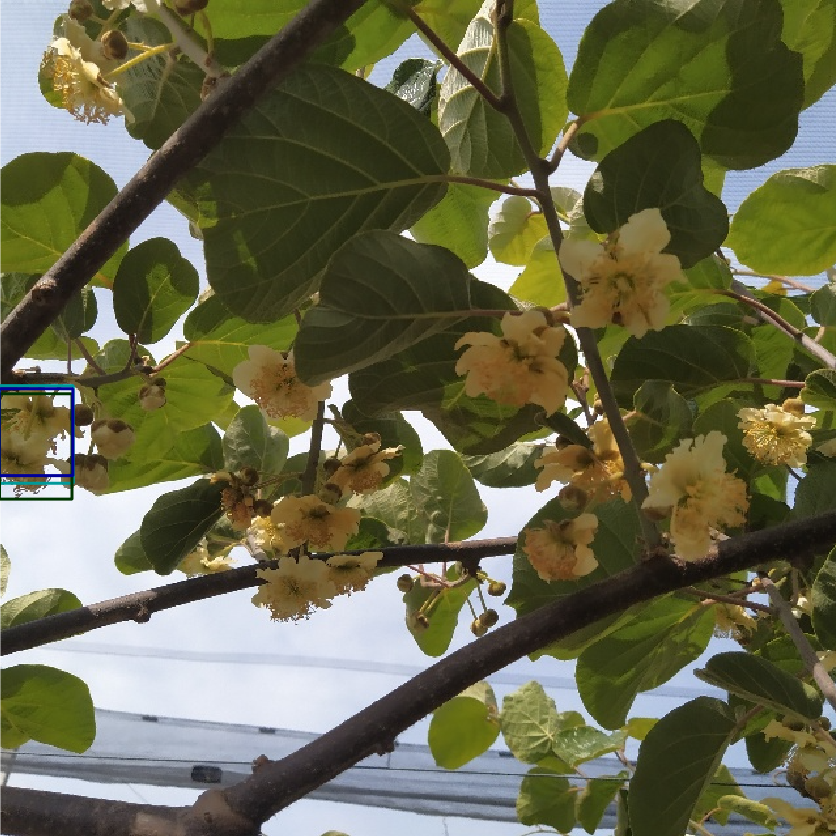

Supplement: Supplementary file 1 — Supplementary Information 1. [file 41598_2024_73035_MOESM1_ESM.zip › images/detr_male_fp4.png]

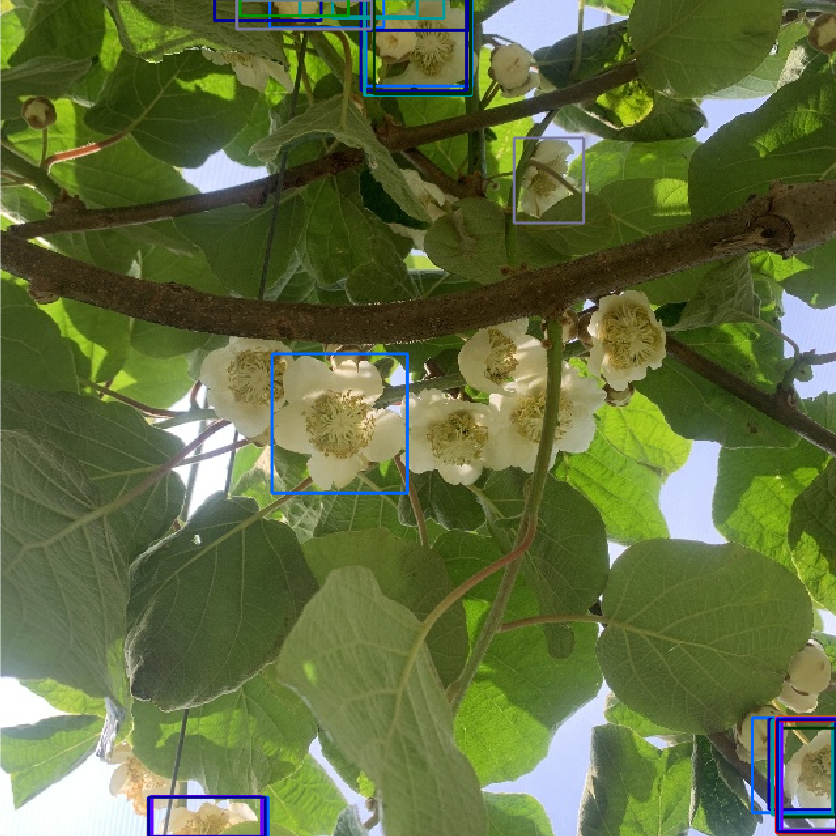

Supplement: Supplementary file 1 — Supplementary Information 1. [file 41598_2024_73035_MOESM1_ESM.zip › images/female_fp1.png]

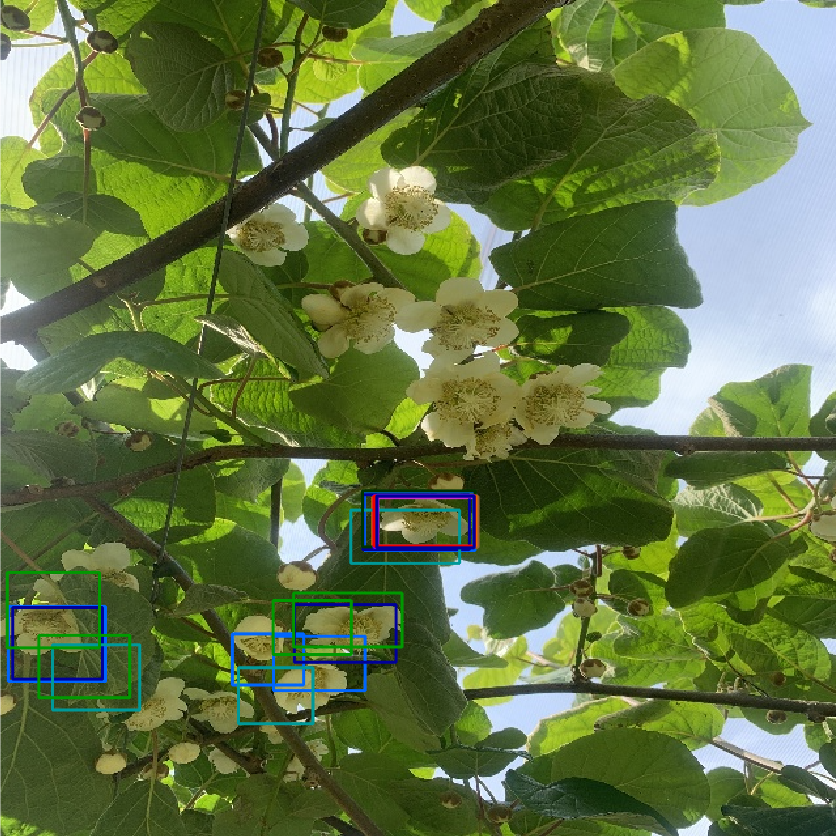

Supplement: Supplementary file 1 — Supplementary Information 1. [file 41598_2024_73035_MOESM1_ESM.zip › images/female_fp2.png]

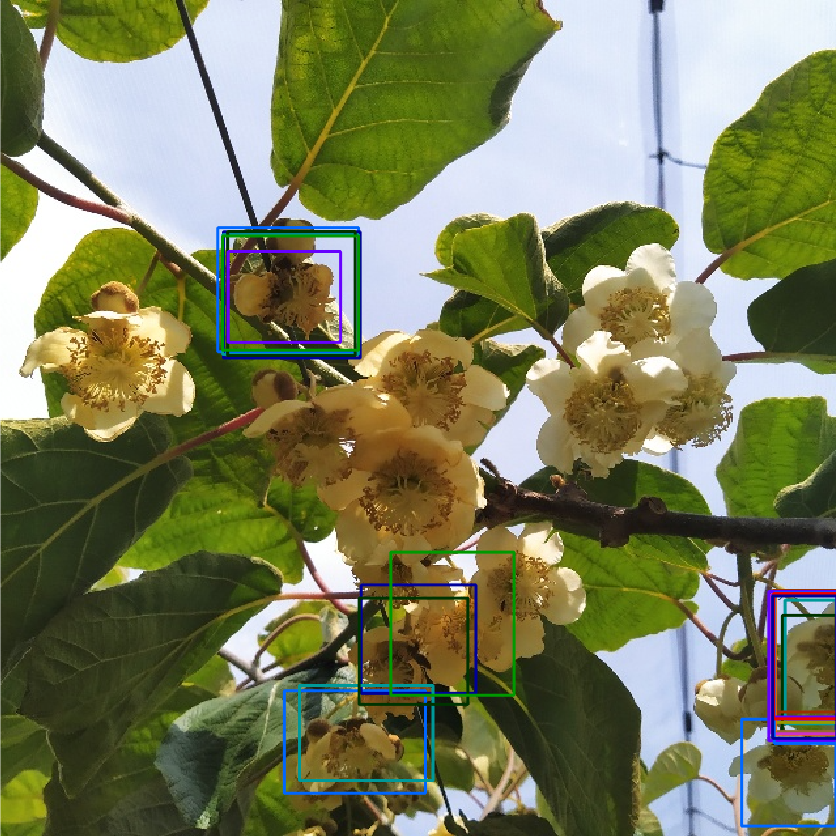

Supplement: Supplementary file 1 — Supplementary Information 1. [file 41598_2024_73035_MOESM1_ESM.zip › images/female_fp3.png]

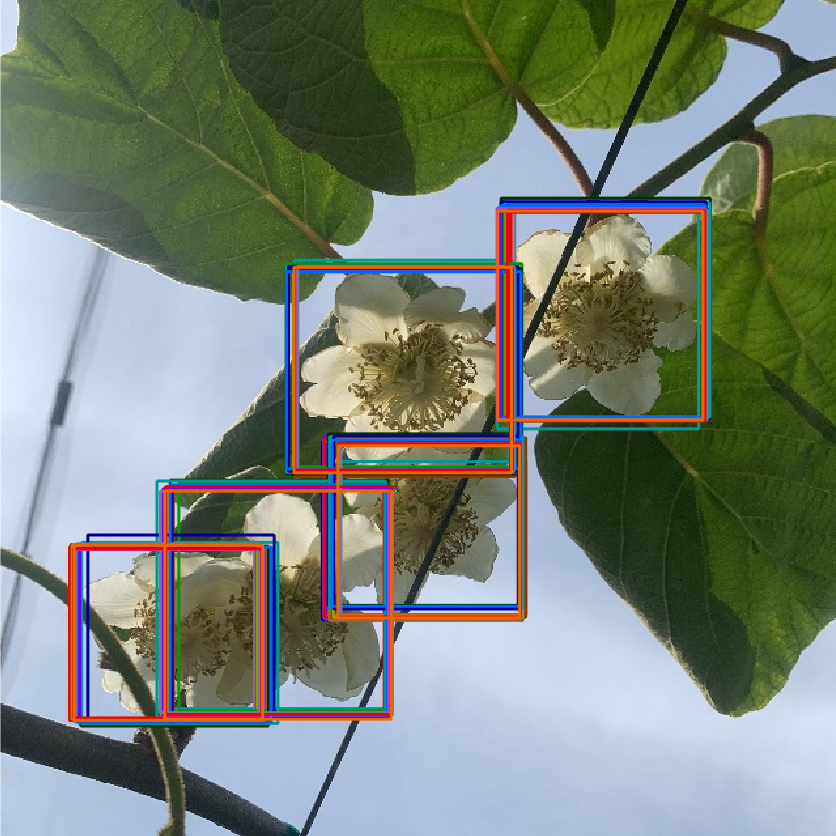

Supplement: Supplementary file 1 — Supplementary Information 1. [file 41598_2024_73035_MOESM1_ESM.zip › images/female_tp1.png]

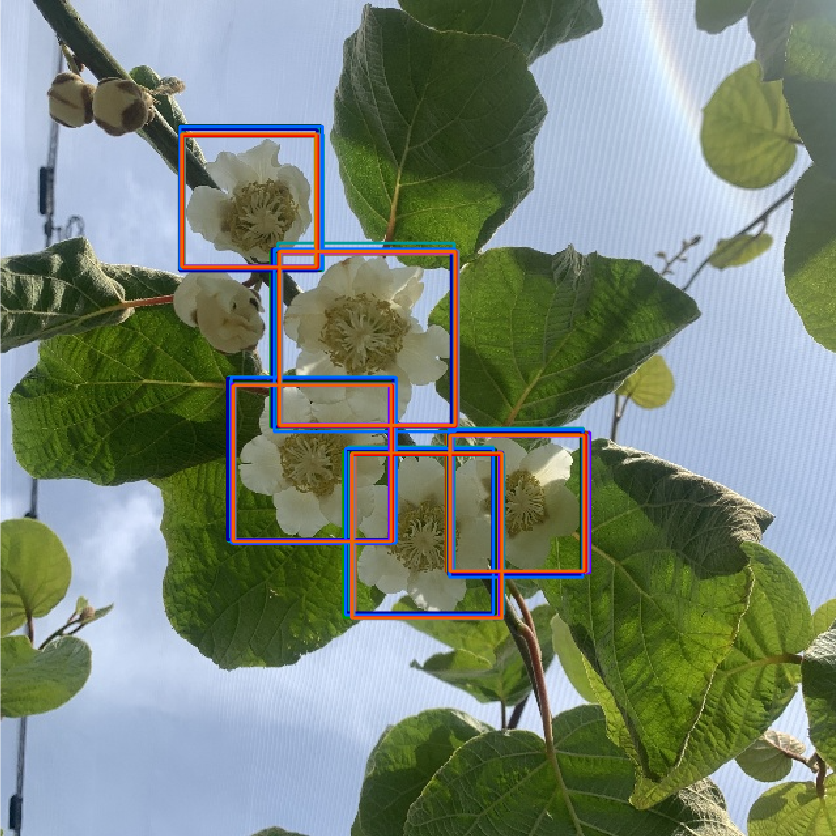

Supplement: Supplementary file 1 — Supplementary Information 1. [file 41598_2024_73035_MOESM1_ESM.zip › images/female_tp2.png]

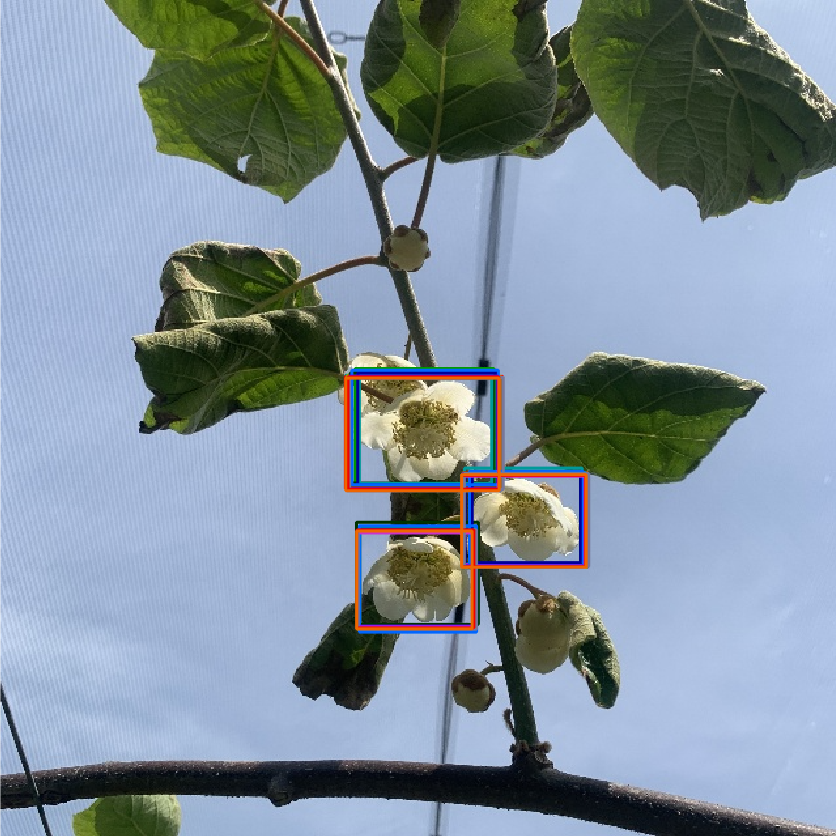

Supplement: Supplementary file 1 — Supplementary Information 1. [file 41598_2024_73035_MOESM1_ESM.zip › images/female_tp3.png]

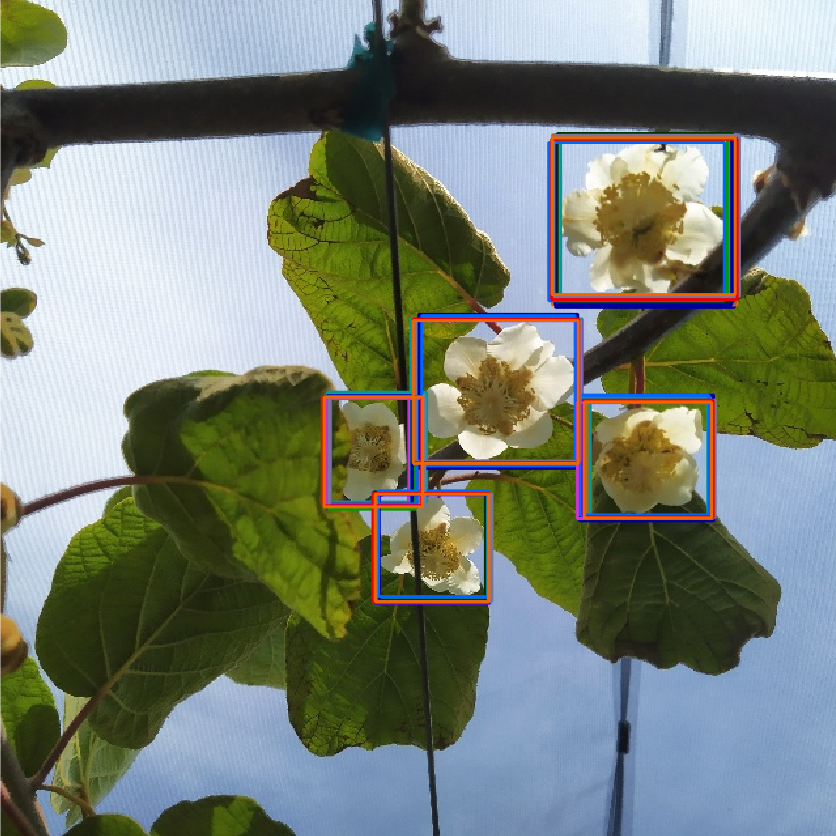

Supplement: Supplementary file 1 — Supplementary Information 1. [file 41598_2024_73035_MOESM1_ESM.zip › images/female_tp4.png]

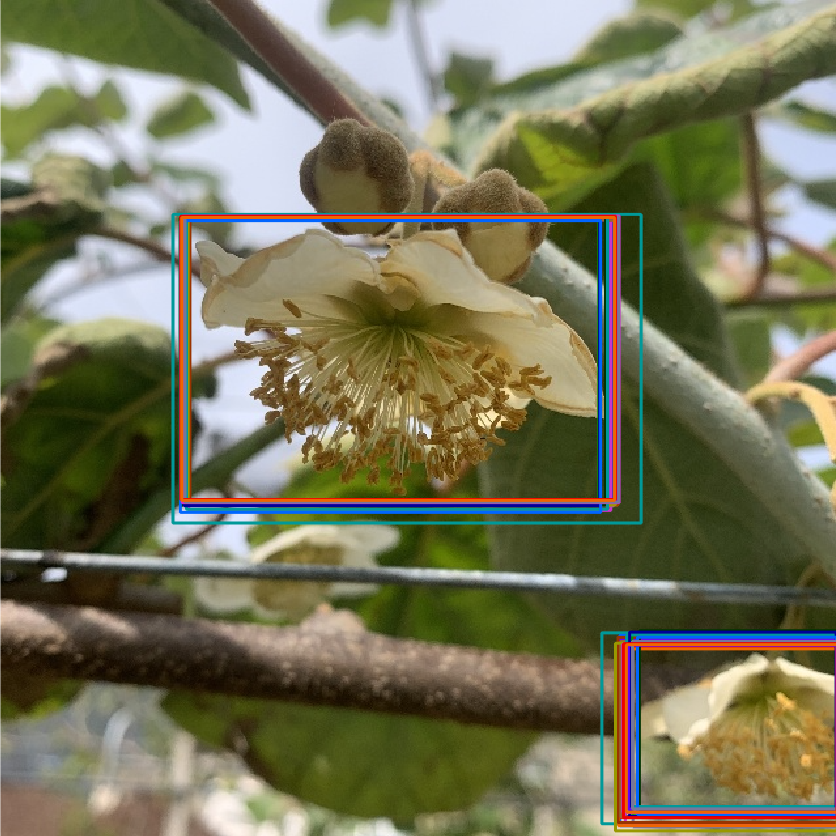

Supplement: Supplementary file 1 — Supplementary Information 1. [file 41598_2024_73035_MOESM1_ESM.zip › images/male_tp1.png]

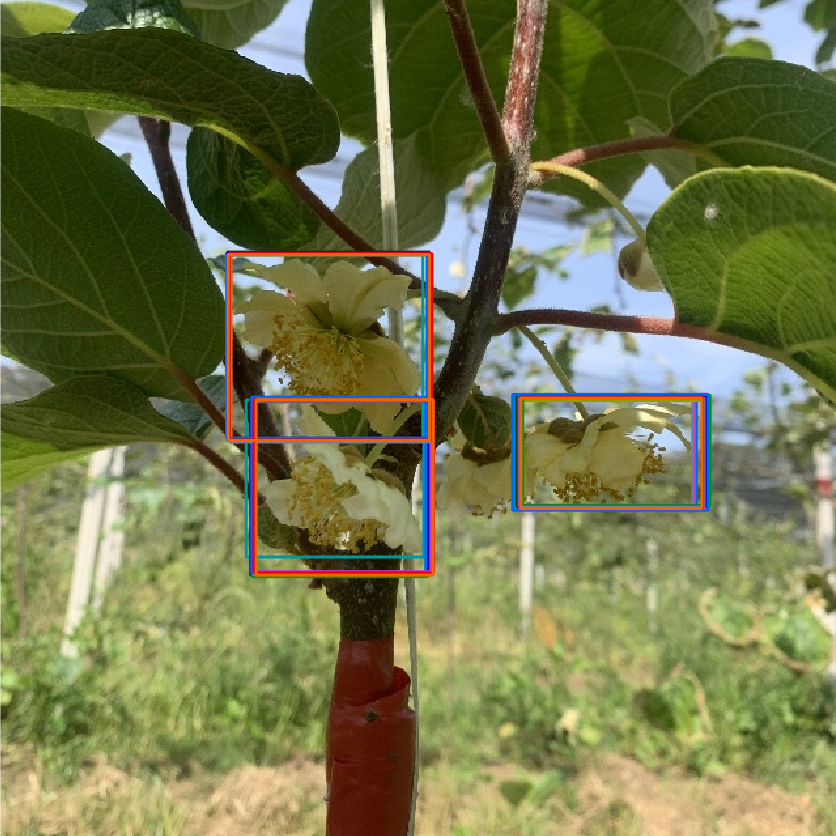

Supplement: Supplementary file 1 — Supplementary Information 1. [file 41598_2024_73035_MOESM1_ESM.zip › images/male_tp2.png]

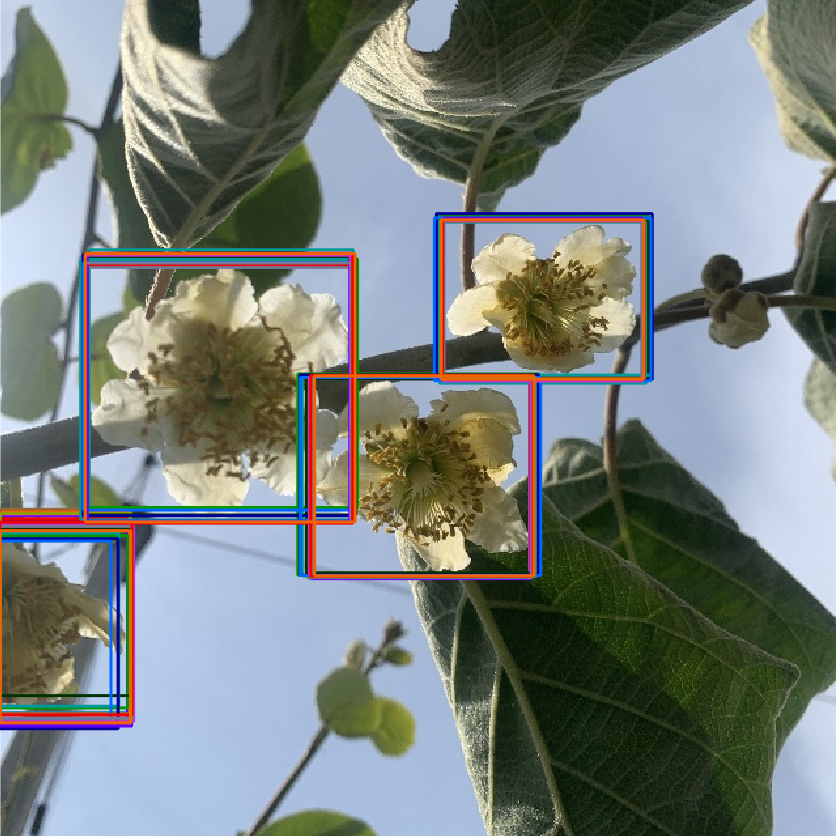

Supplement: Supplementary file 1 — Supplementary Information 1. [file 41598_2024_73035_MOESM1_ESM.zip › images/male_tp3.png]

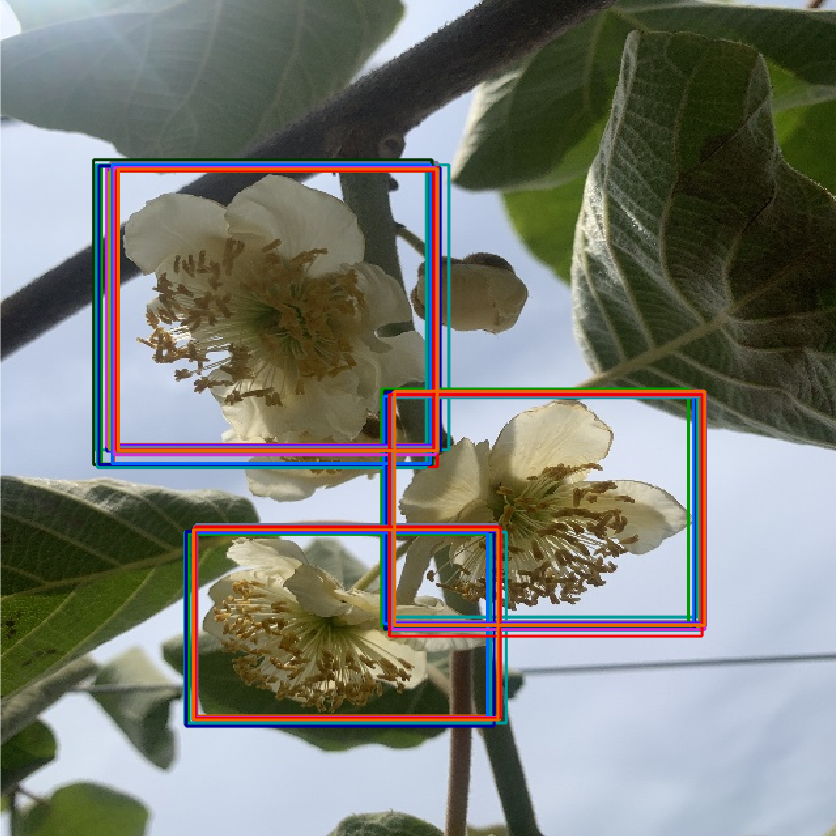

Supplement: Supplementary file 1 — Supplementary Information 1. [file 41598_2024_73035_MOESM1_ESM.zip › images/male_tp4.png]

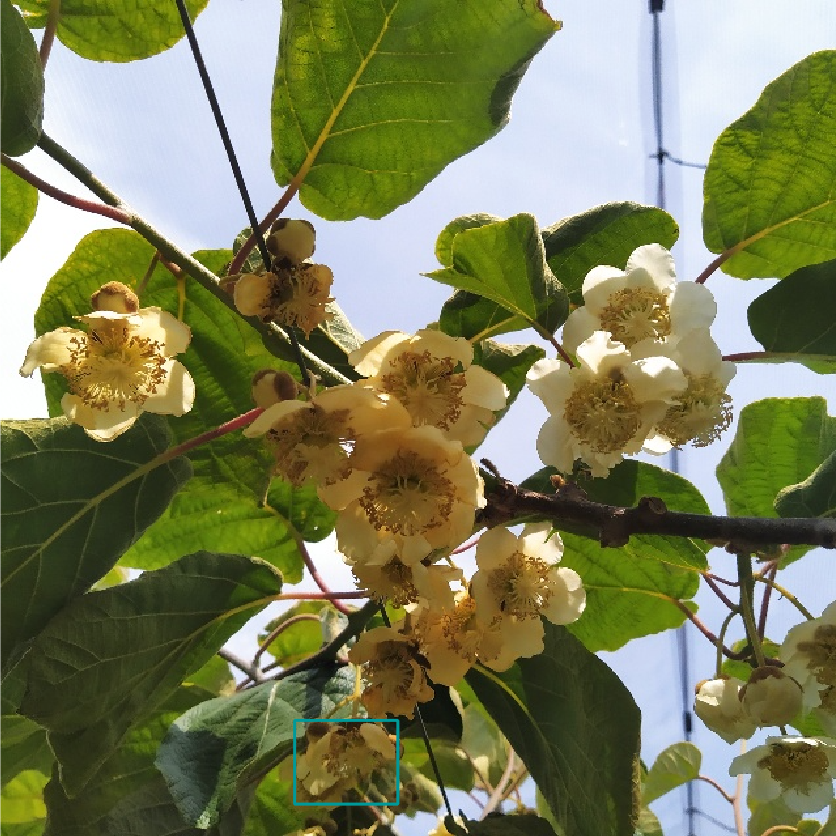

Supplement: Supplementary file 1 — Supplementary Information 1. [file 41598_2024_73035_MOESM1_ESM.zip › images/rtdetr_female_fn1.png]

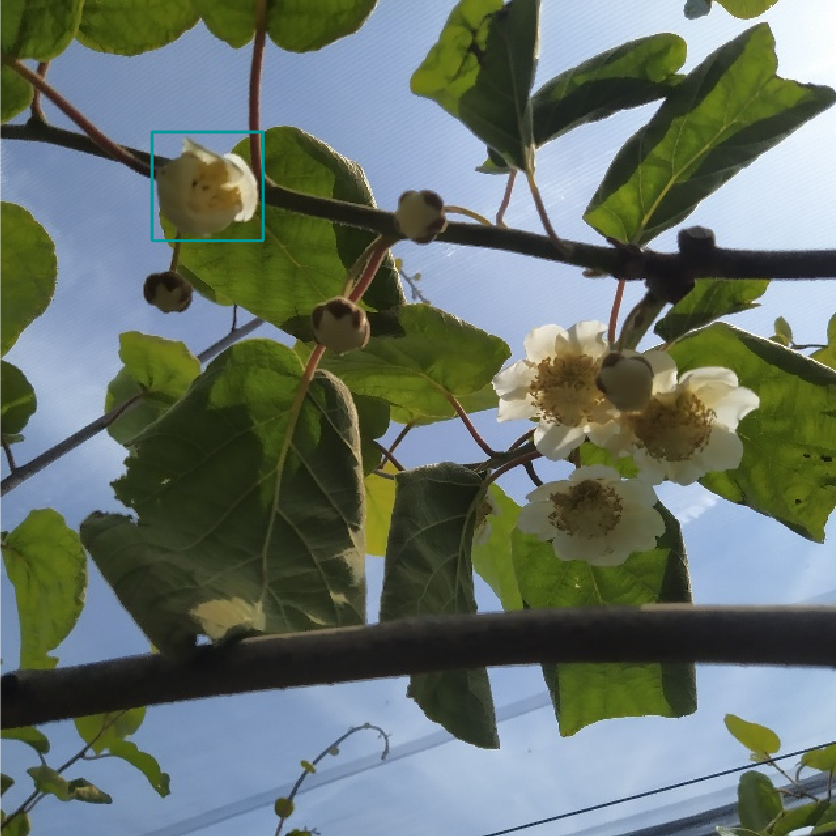

Supplement: Supplementary file 1 — Supplementary Information 1. [file 41598_2024_73035_MOESM1_ESM.zip › images/rtdetr_female_fn2.png]

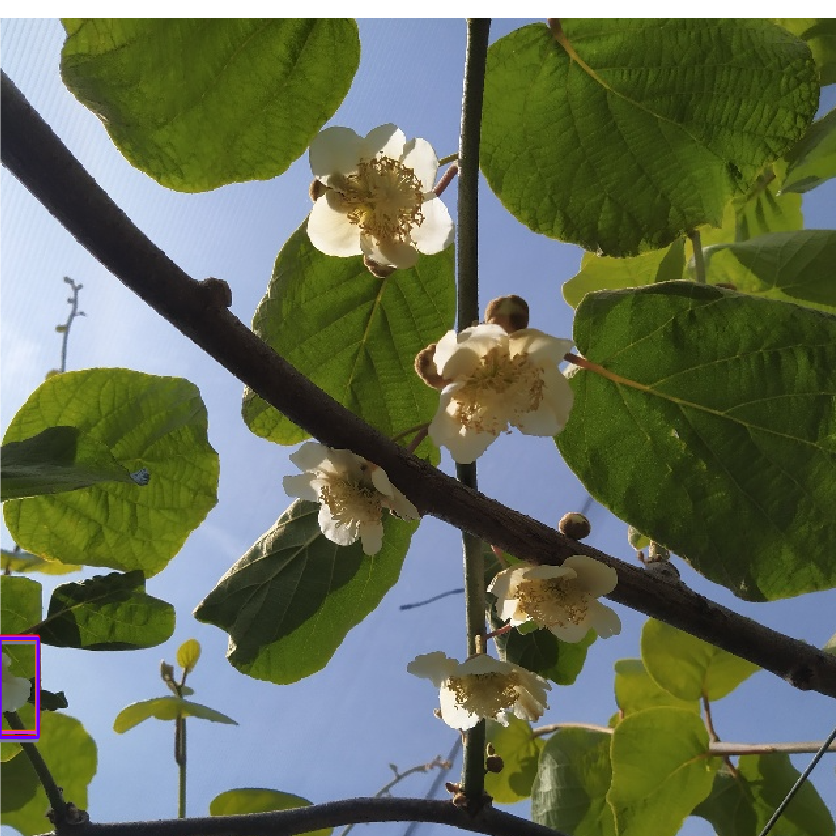

Supplement: Supplementary file 1 — Supplementary Information 1. [file 41598_2024_73035_MOESM1_ESM.zip › images/rtdetr_female_fp1.png]

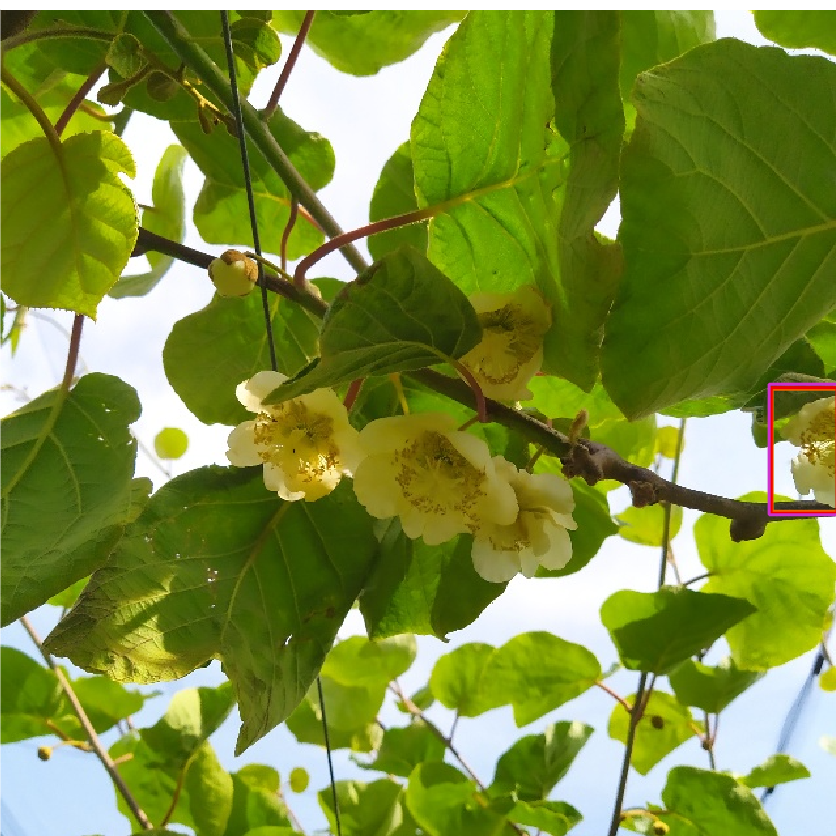

Supplement: Supplementary file 1 — Supplementary Information 1. [file 41598_2024_73035_MOESM1_ESM.zip › images/rtdetr_female_fp2.png]

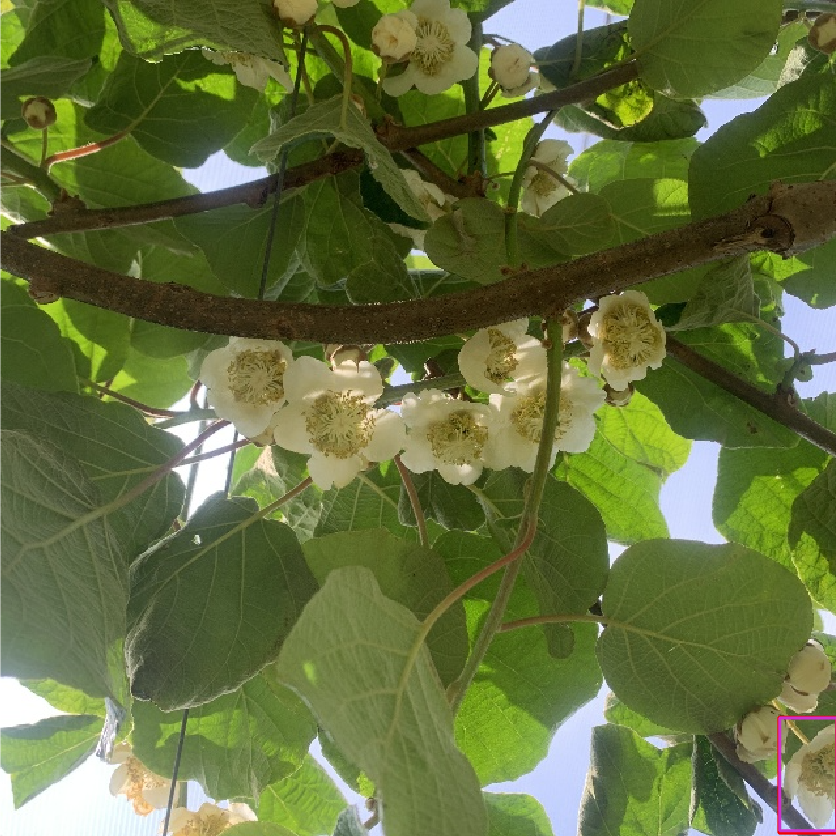

Supplement: Supplementary file 1 — Supplementary Information 1. [file 41598_2024_73035_MOESM1_ESM.zip › images/rtdetr_female_fp3.png]

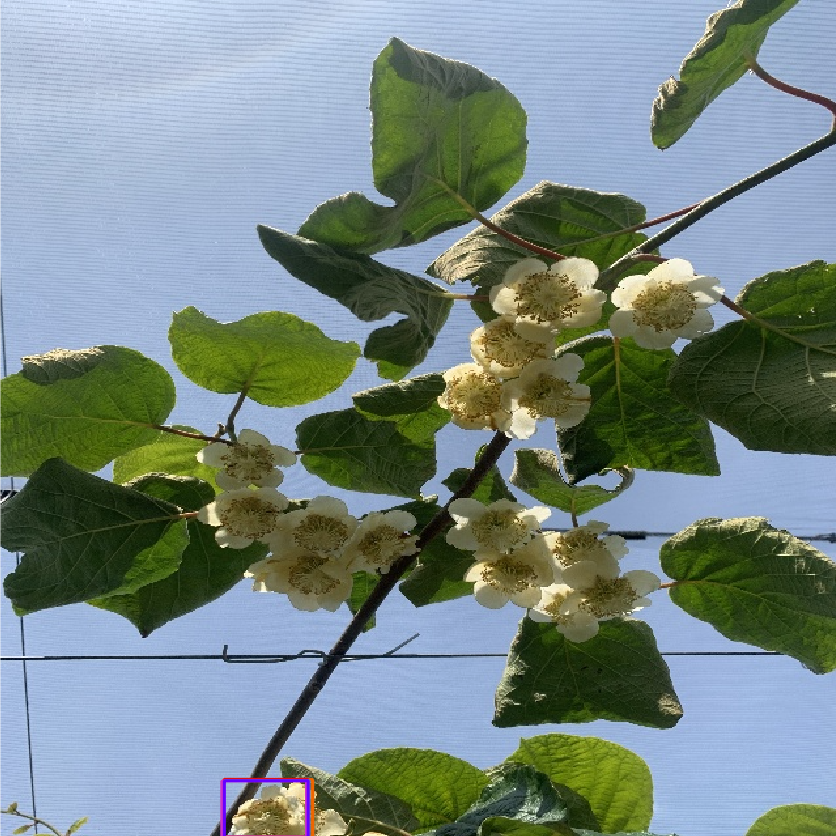

Supplement: Supplementary file 1 — Supplementary Information 1. [file 41598_2024_73035_MOESM1_ESM.zip › images/rtdetr_female_fp4.png]

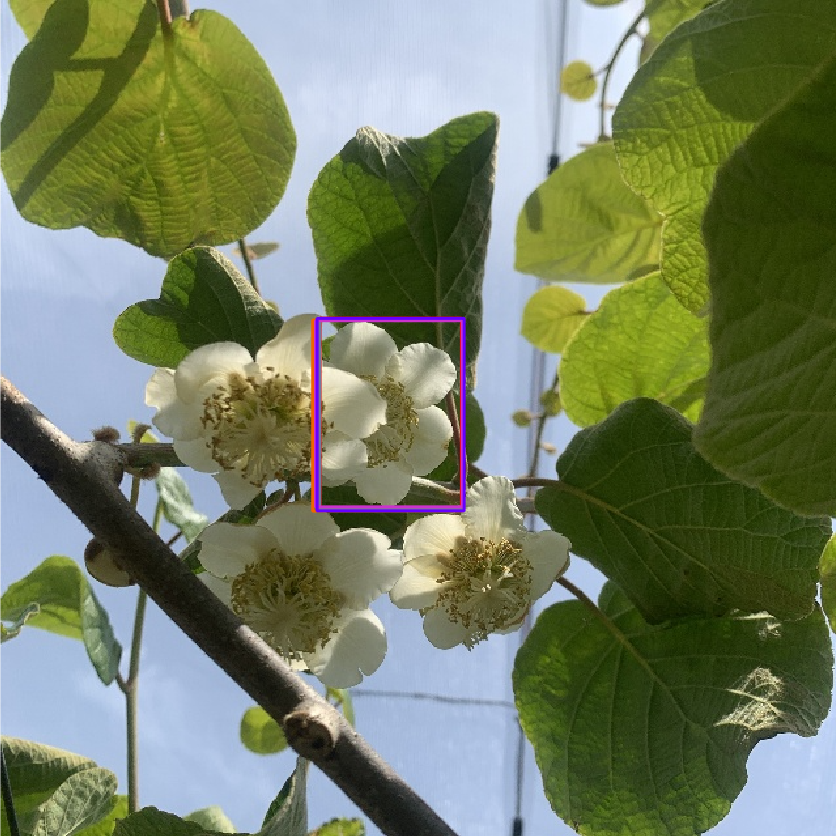

Supplement: Supplementary file 1 — Supplementary Information 1. [file 41598_2024_73035_MOESM1_ESM.zip › images/rtdetr_female_fp5.png]

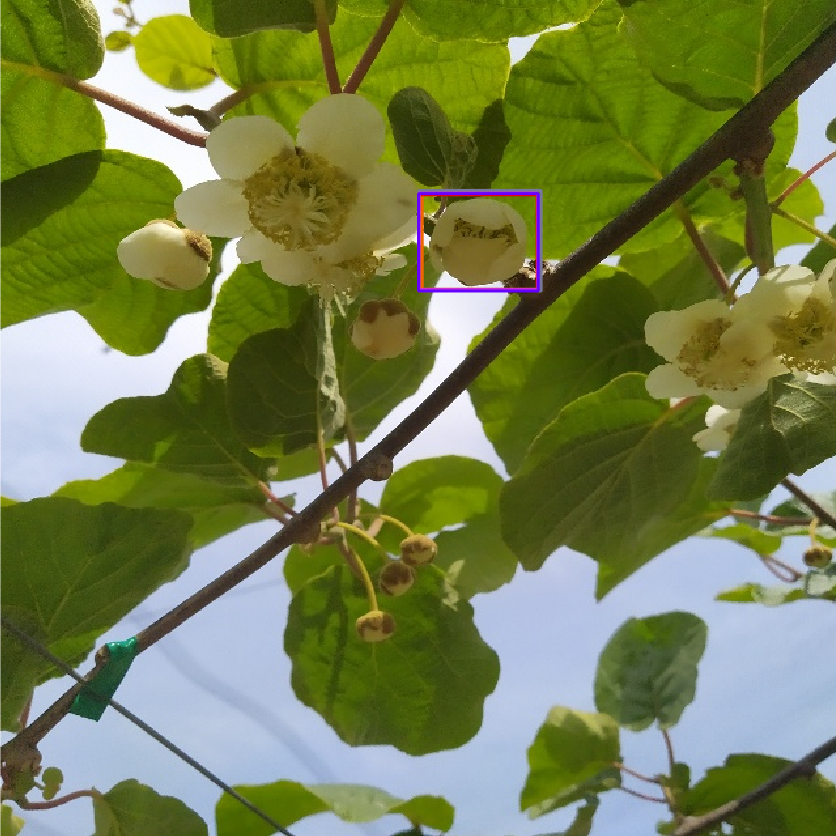

Supplement: Supplementary file 1 — Supplementary Information 1. [file 41598_2024_73035_MOESM1_ESM.zip › images/rtdetr_female_fp6.png]

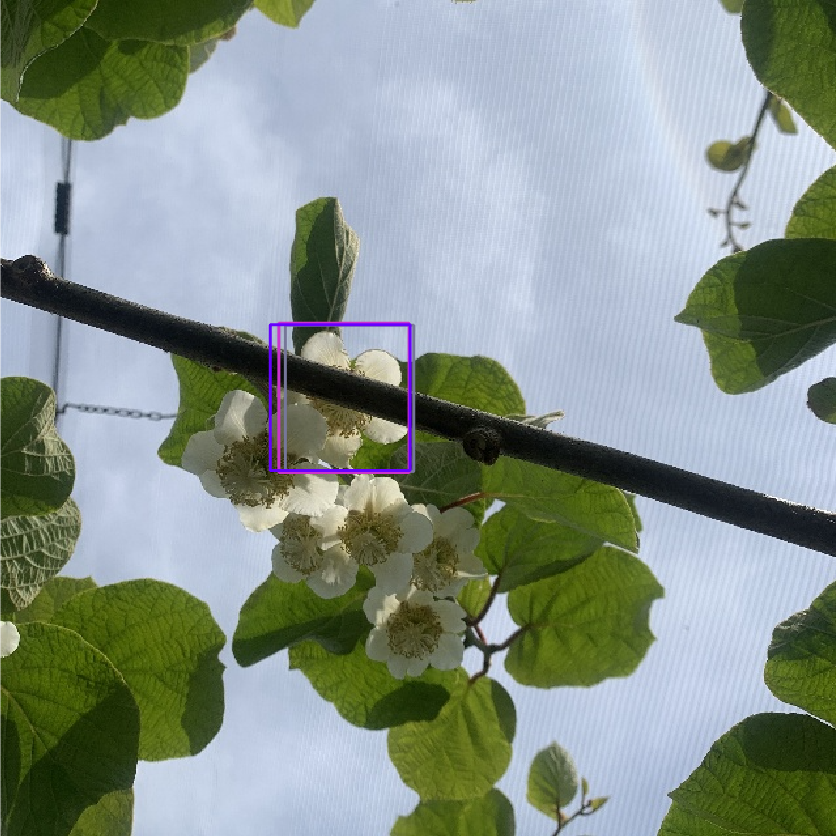

Supplement: Supplementary file 1 — Supplementary Information 1. [file 41598_2024_73035_MOESM1_ESM.zip › images/rtdetr_female_fp7.png]

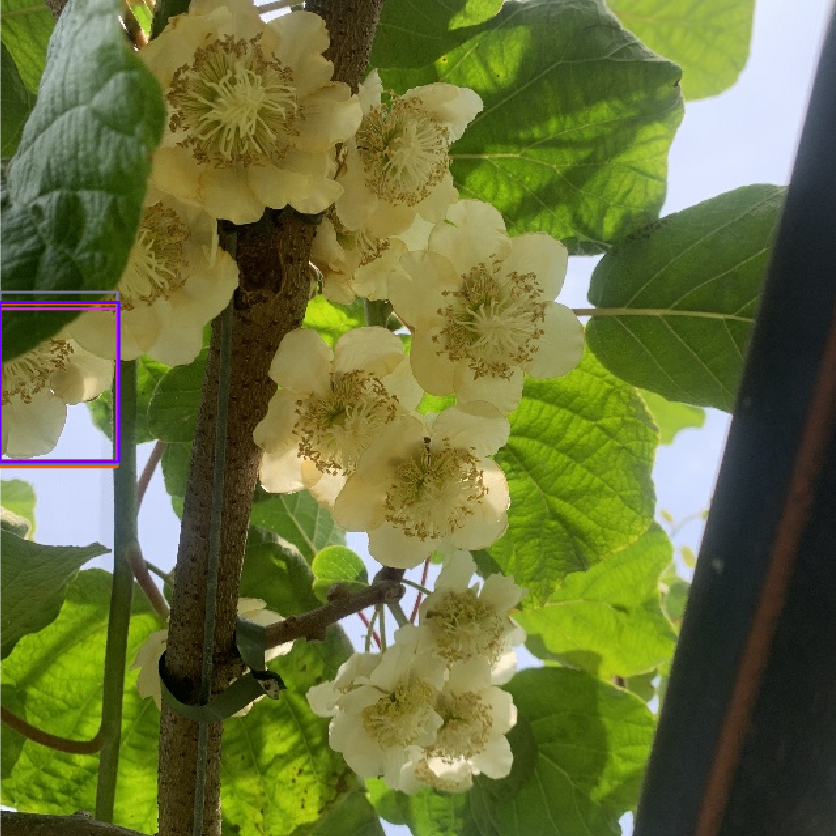

Supplement: Supplementary file 1 — Supplementary Information 1. [file 41598_2024_73035_MOESM1_ESM.zip › images/rtdetr_female_fp8.png]

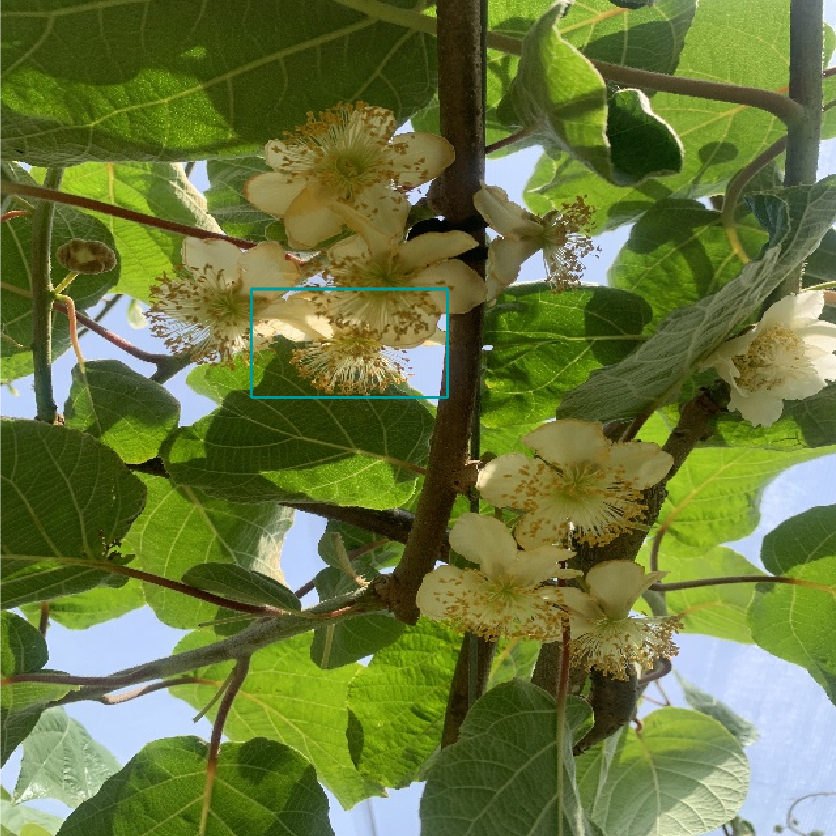

Supplement: Supplementary file 1 — Supplementary Information 1. [file 41598_2024_73035_MOESM1_ESM.zip › images/rtdetr_male_fn1.png]

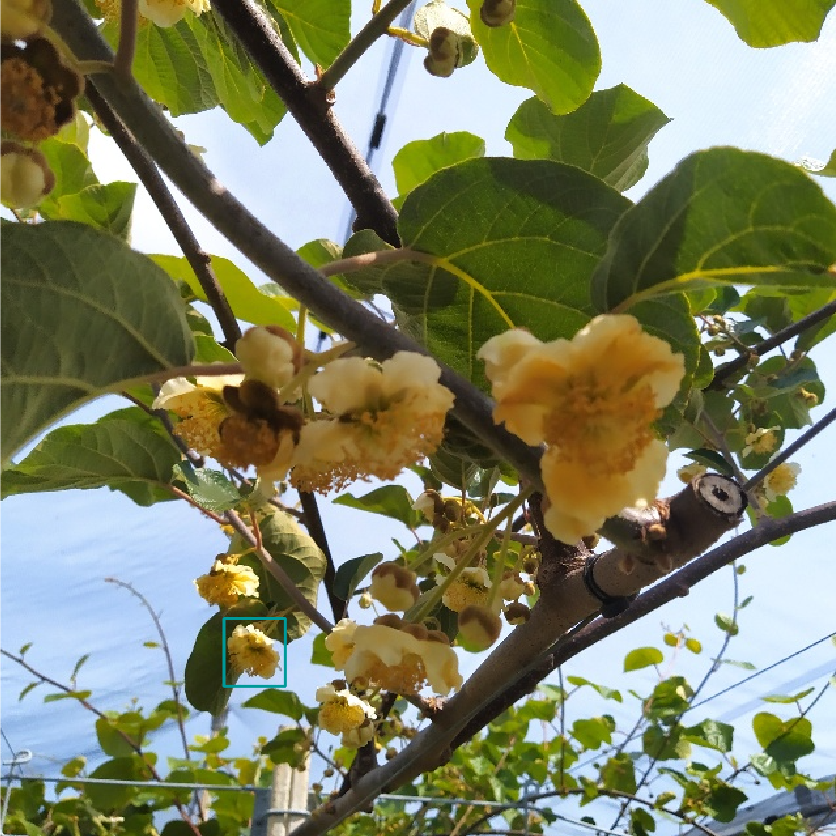

Supplement: Supplementary file 1 — Supplementary Information 1. [file 41598_2024_73035_MOESM1_ESM.zip › images/rtdetr_male_fn2.png]

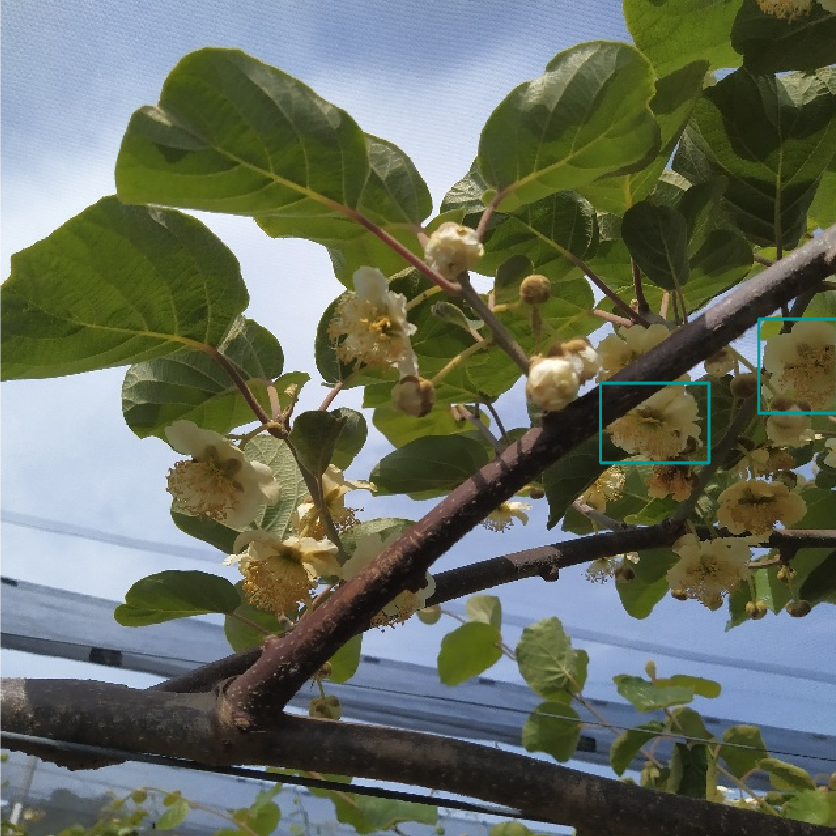

Supplement: Supplementary file 1 — Supplementary Information 1. [file 41598_2024_73035_MOESM1_ESM.zip › images/rtdetr_male_fn3.png]

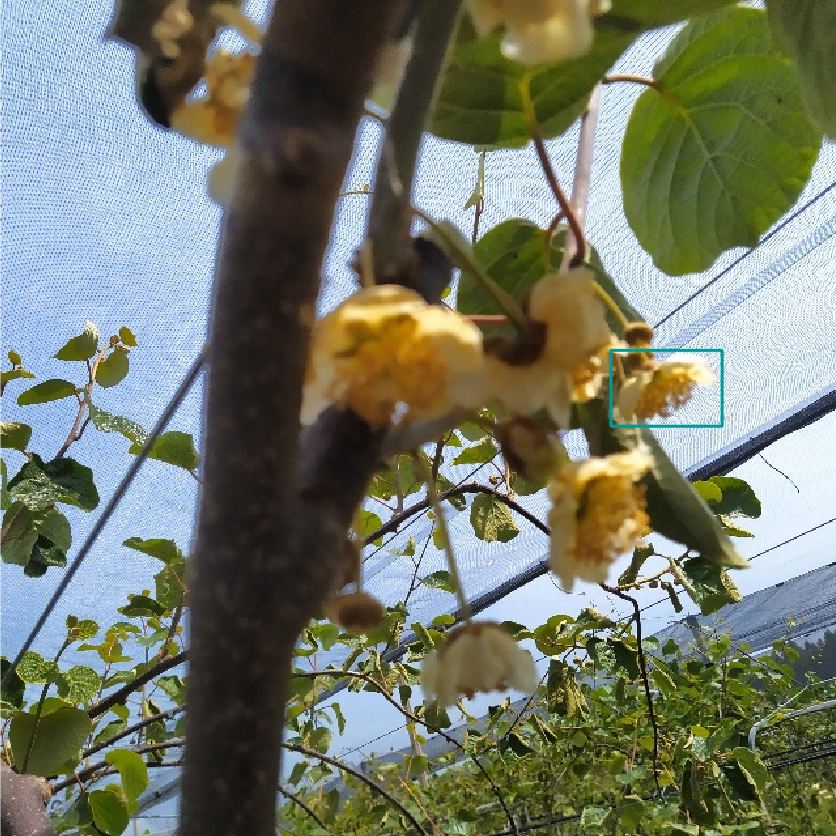

Supplement: Supplementary file 1 — Supplementary Information 1. [file 41598_2024_73035_MOESM1_ESM.zip › images/rtdetr_male_fn4.png]

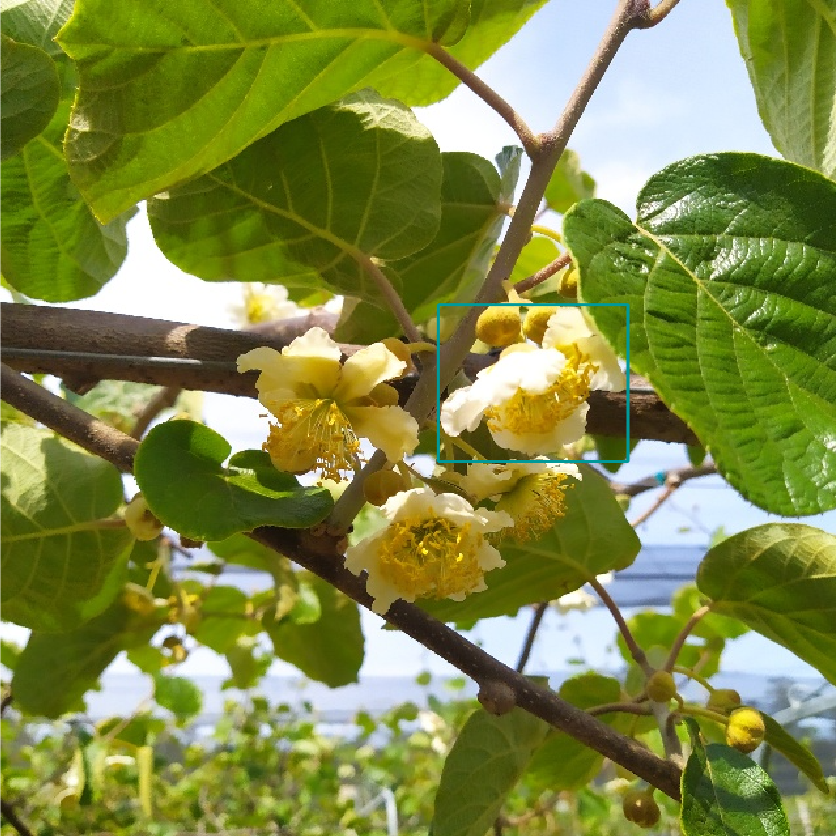

Supplement: Supplementary file 1 — Supplementary Information 1. [file 41598_2024_73035_MOESM1_ESM.zip › images/rtdetr_male_fn5.png]

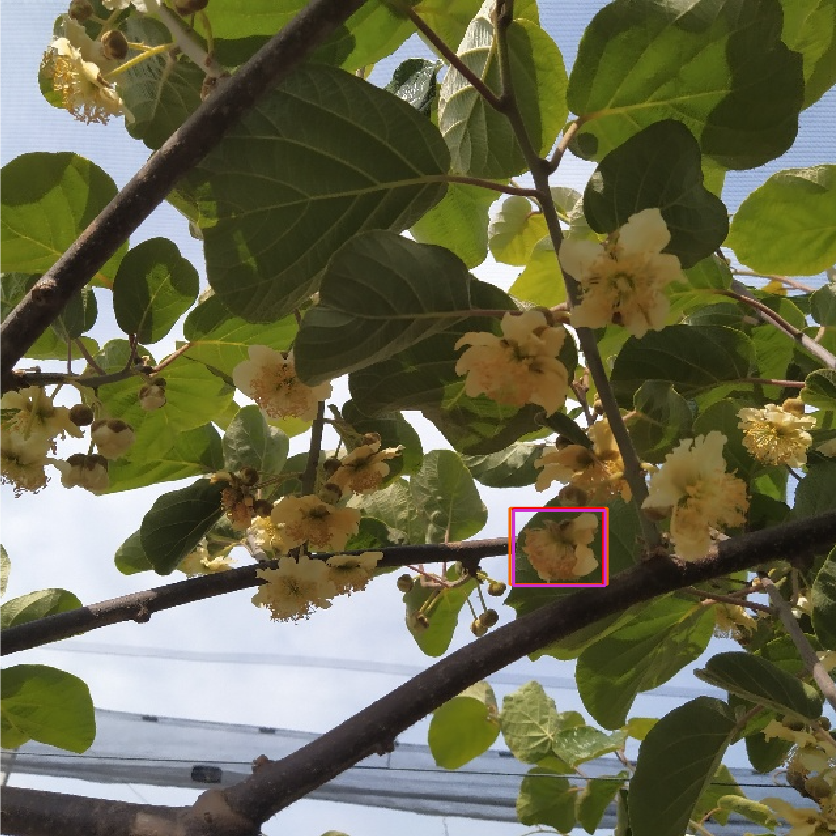

Supplement: Supplementary file 1 — Supplementary Information 1. [file 41598_2024_73035_MOESM1_ESM.zip › images/rtdetr_male_fp1.png]

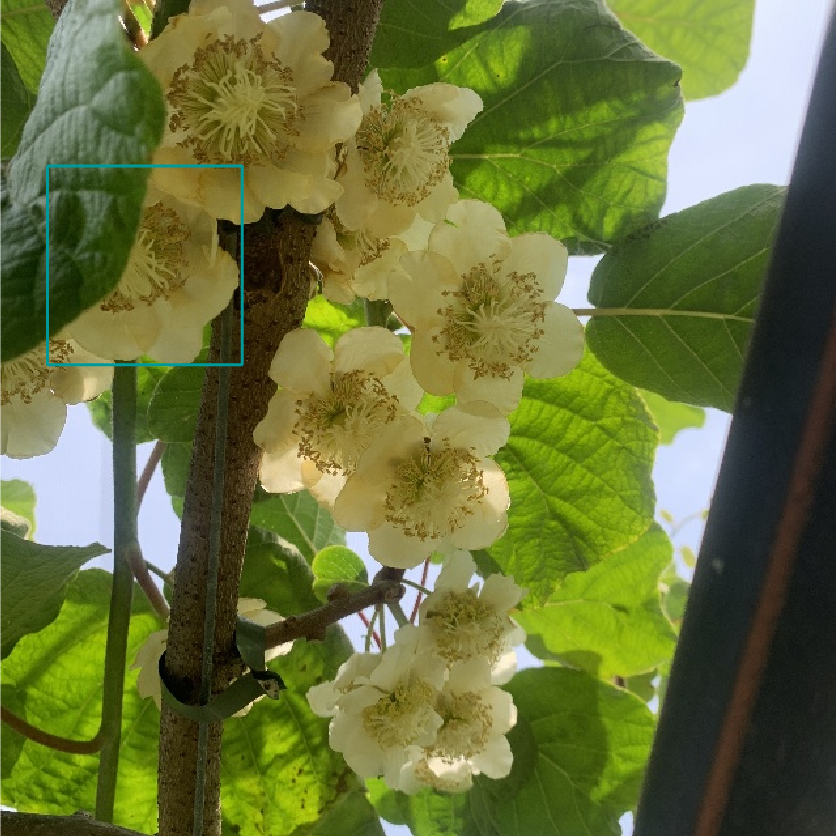

Supplement: Supplementary file 1 — Supplementary Information 1. [file 41598_2024_73035_MOESM1_ESM.zip › images/yolov5_female_fn1.png]

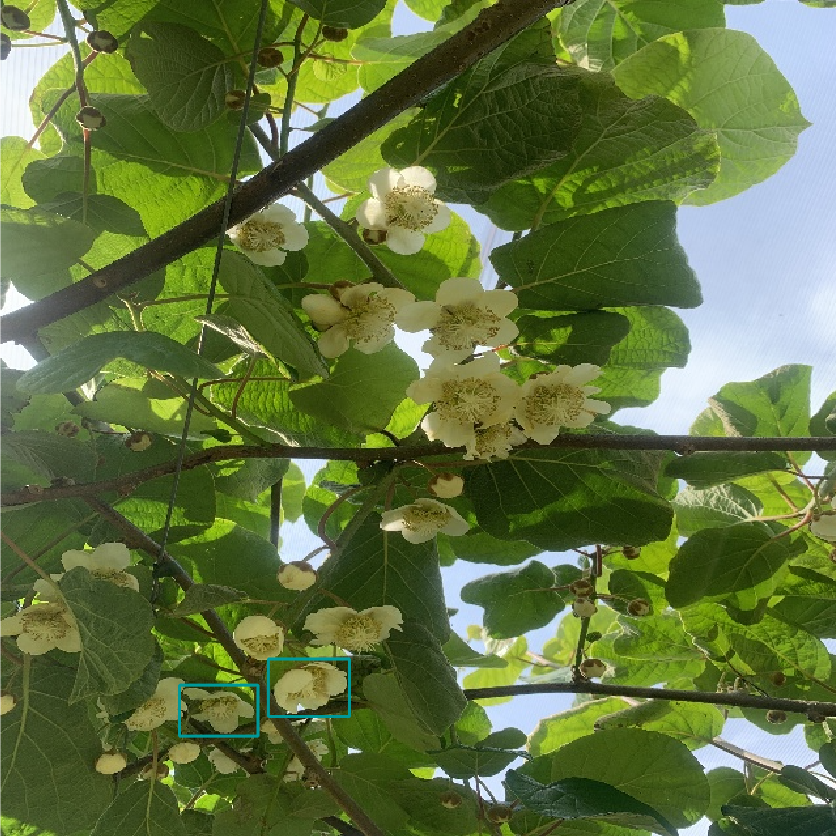

Supplement: Supplementary file 1 — Supplementary Information 1. [file 41598_2024_73035_MOESM1_ESM.zip › images/yolov5_female_fn2.png]

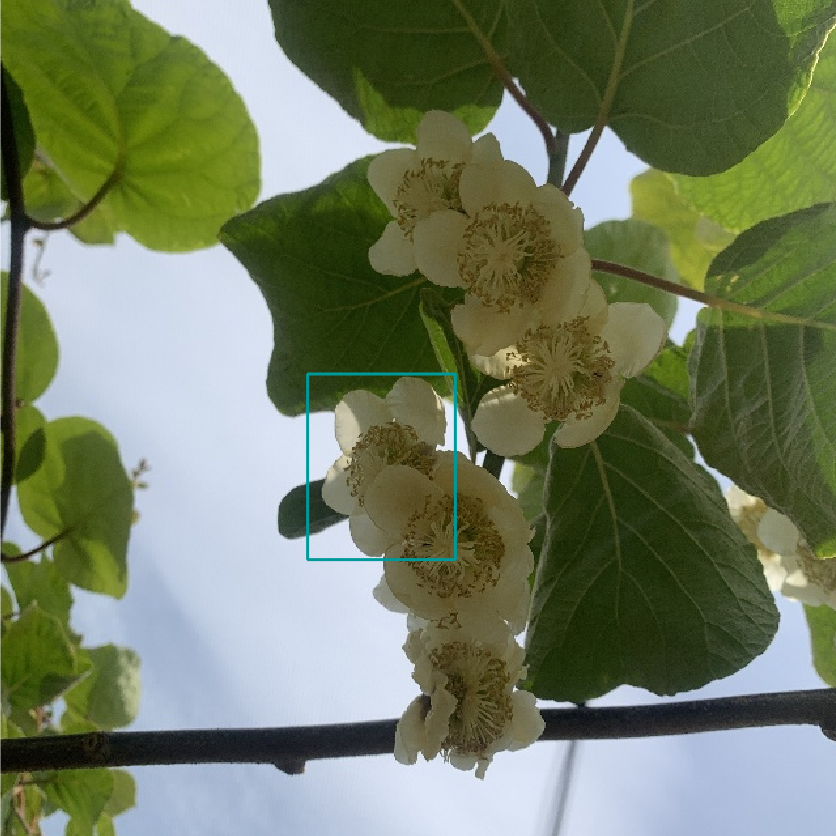

Supplement: Supplementary file 1 — Supplementary Information 1. [file 41598_2024_73035_MOESM1_ESM.zip › images/yolov5_female_fn3.png]

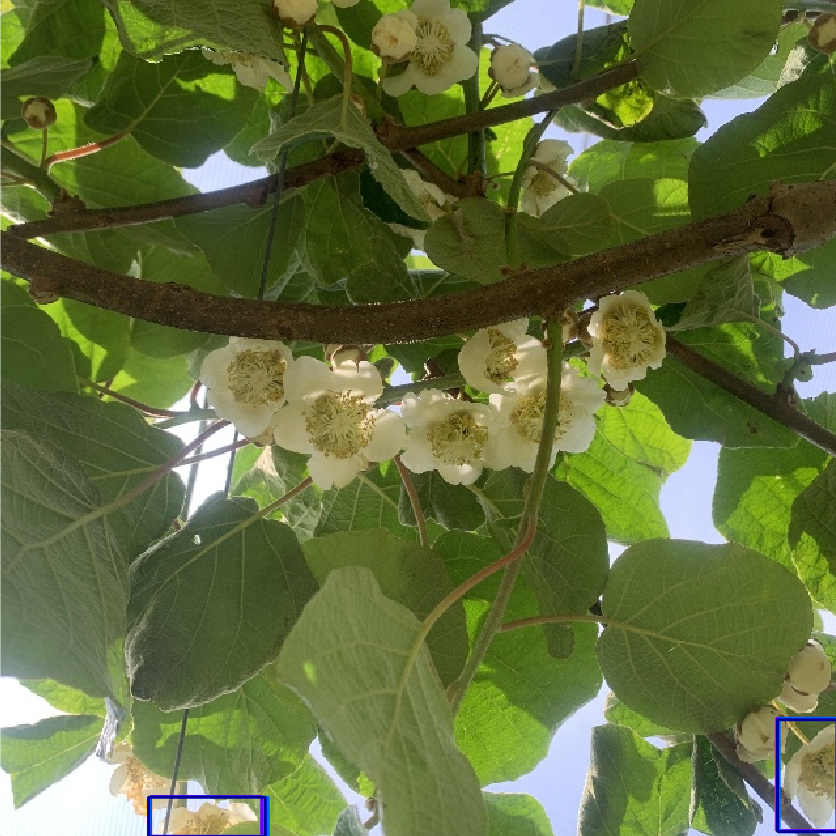

Supplement: Supplementary file 1 — Supplementary Information 1. [file 41598_2024_73035_MOESM1_ESM.zip › images/yolov5_female_fp1.png]

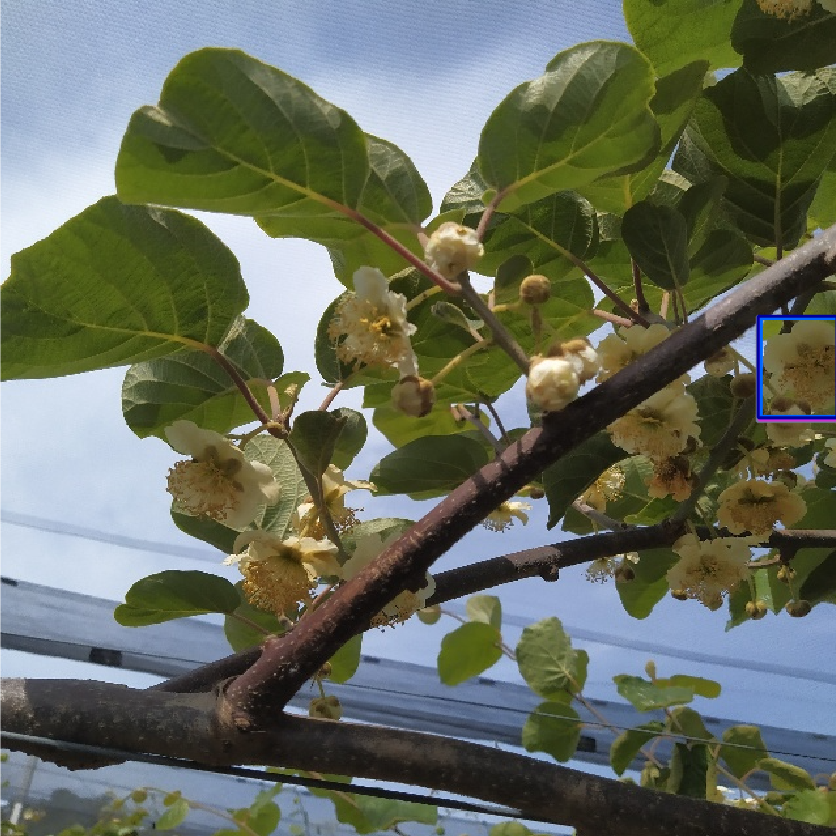

Supplement: Supplementary file 1 — Supplementary Information 1. [file 41598_2024_73035_MOESM1_ESM.zip › images/yolov5_female_fp2.png]

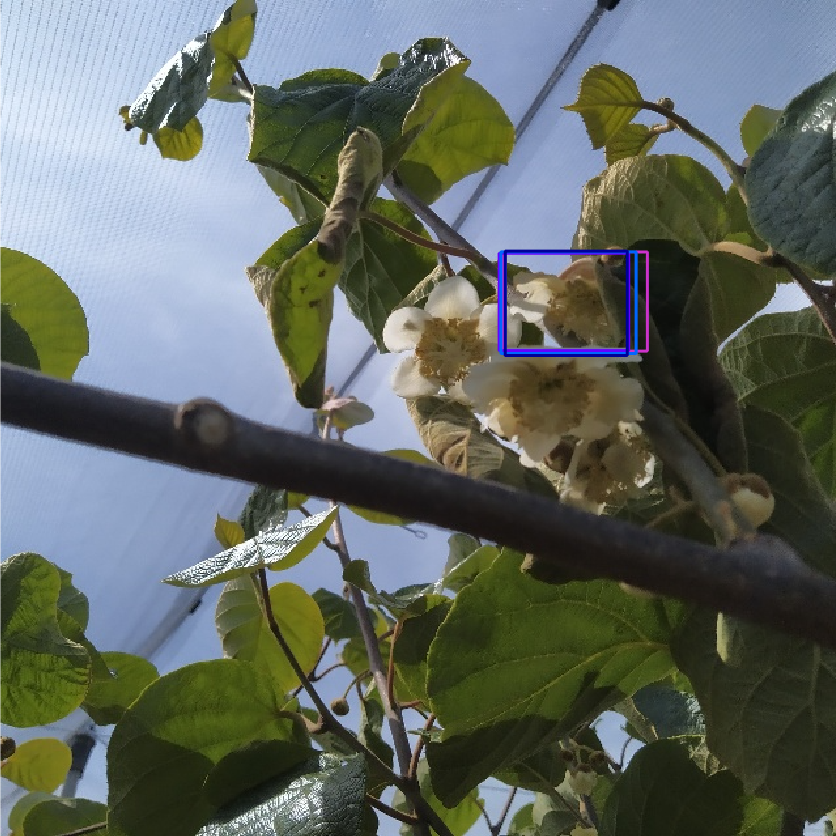

Supplement: Supplementary file 1 — Supplementary Information 1. [file 41598_2024_73035_MOESM1_ESM.zip › images/yolov5_female_fp3.png]

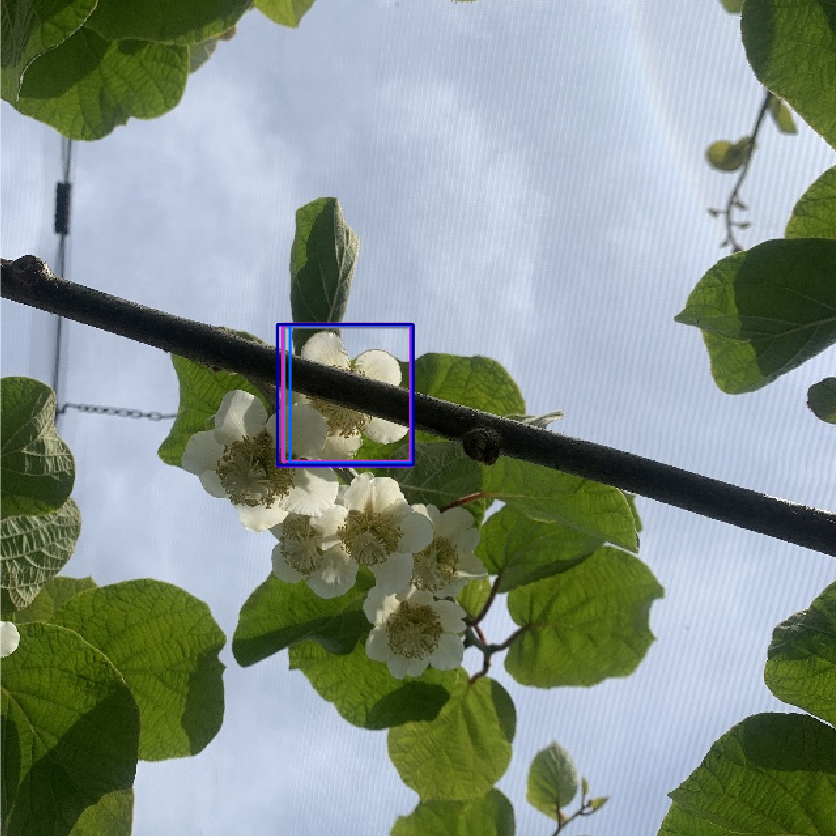

Supplement: Supplementary file 1 — Supplementary Information 1. [file 41598_2024_73035_MOESM1_ESM.zip › images/yolov5_female_fp4.png]

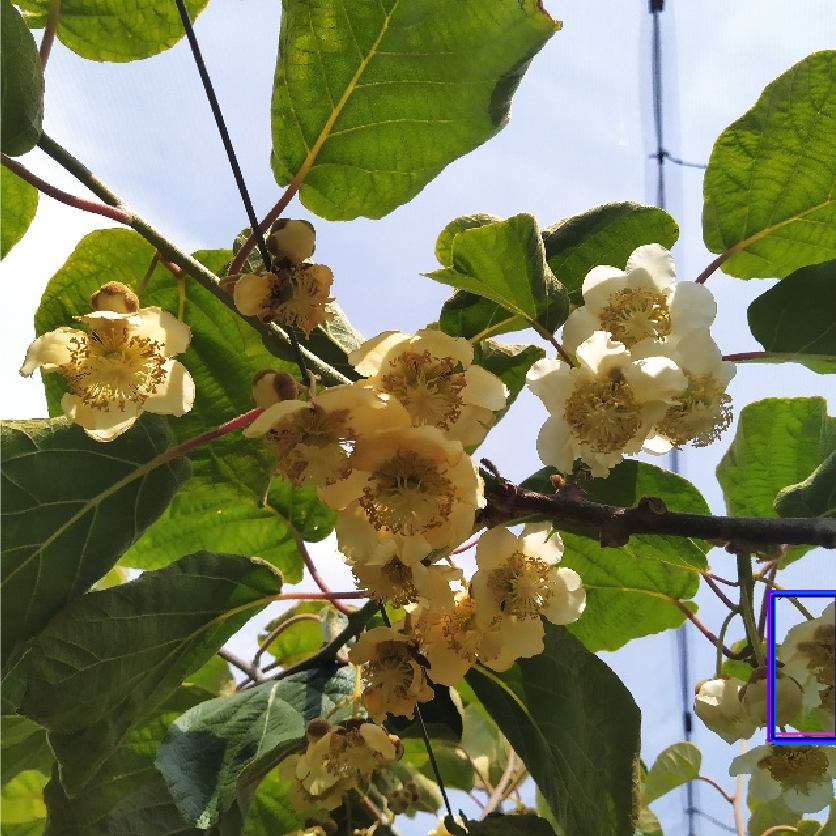

Supplement: Supplementary file 1 — Supplementary Information 1. [file 41598_2024_73035_MOESM1_ESM.zip › images/yolov5_female_fp5.png]

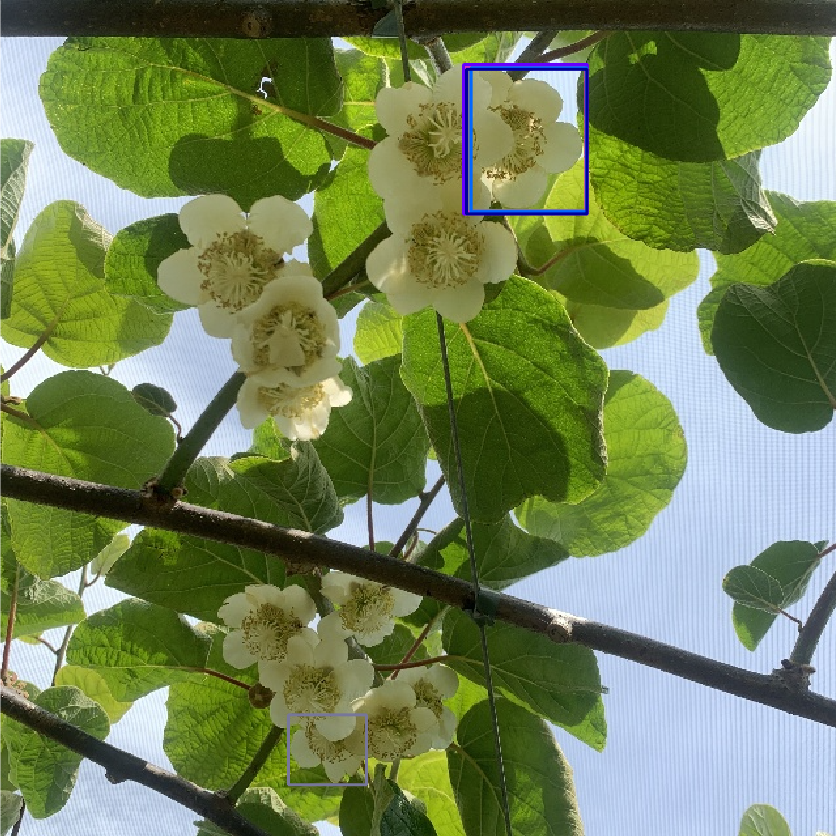

Supplement: Supplementary file 1 — Supplementary Information 1. [file 41598_2024_73035_MOESM1_ESM.zip › images/yolov5_female_fp6.png]

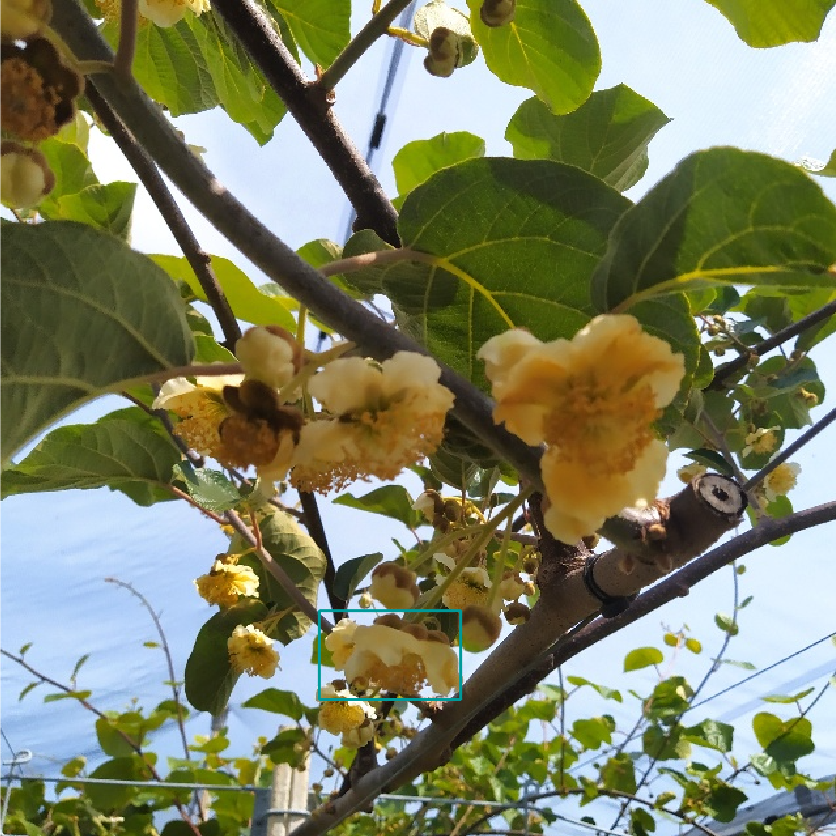

Supplement: Supplementary file 1 — Supplementary Information 1. [file 41598_2024_73035_MOESM1_ESM.zip › images/yolov5_male_fn1.png]

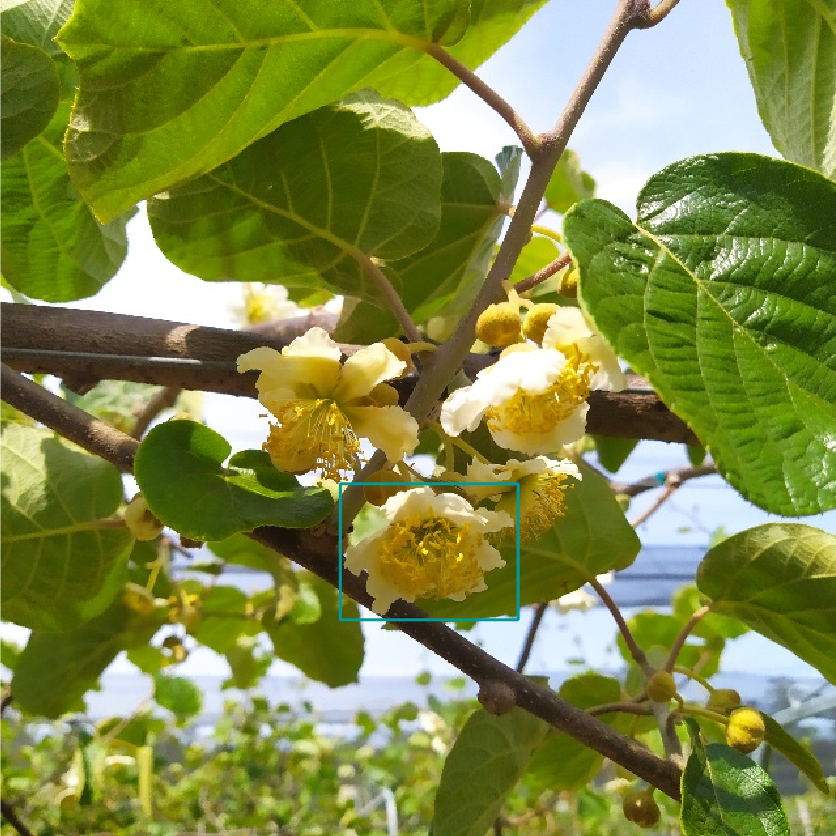

Supplement: Supplementary file 1 — Supplementary Information 1. [file 41598_2024_73035_MOESM1_ESM.zip › images/yolov5_male_fn4.png]

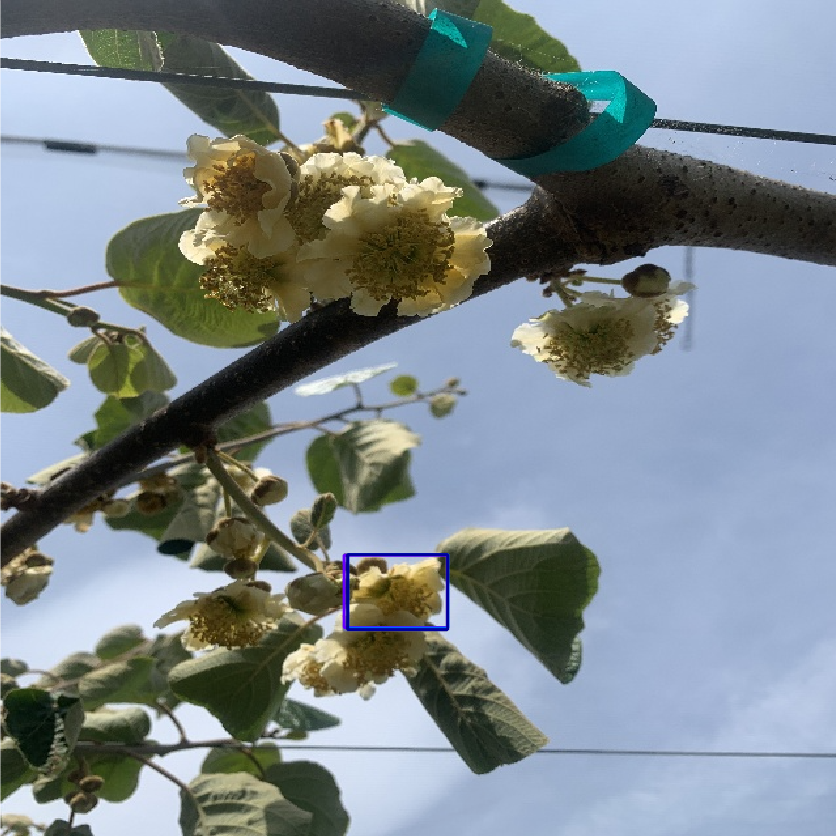

Supplement: Supplementary file 1 — Supplementary Information 1. [file 41598_2024_73035_MOESM1_ESM.zip › images/yolov5_male_fp1.png]

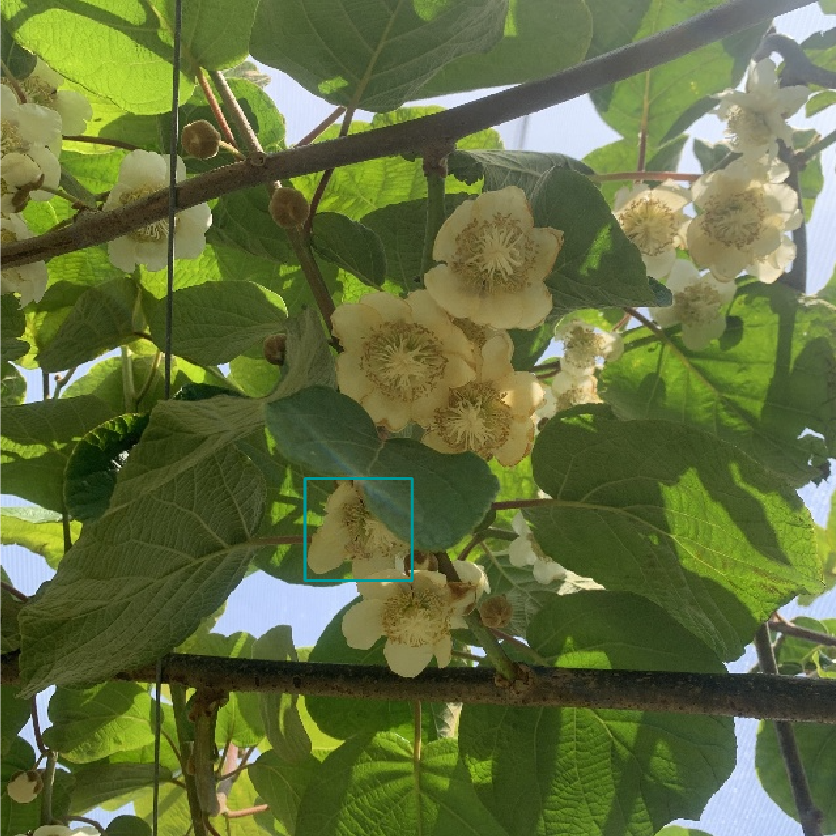

Supplement: Supplementary file 1 — Supplementary Information 1. [file 41598_2024_73035_MOESM1_ESM.zip › images/yolov8_female_fn2.png]

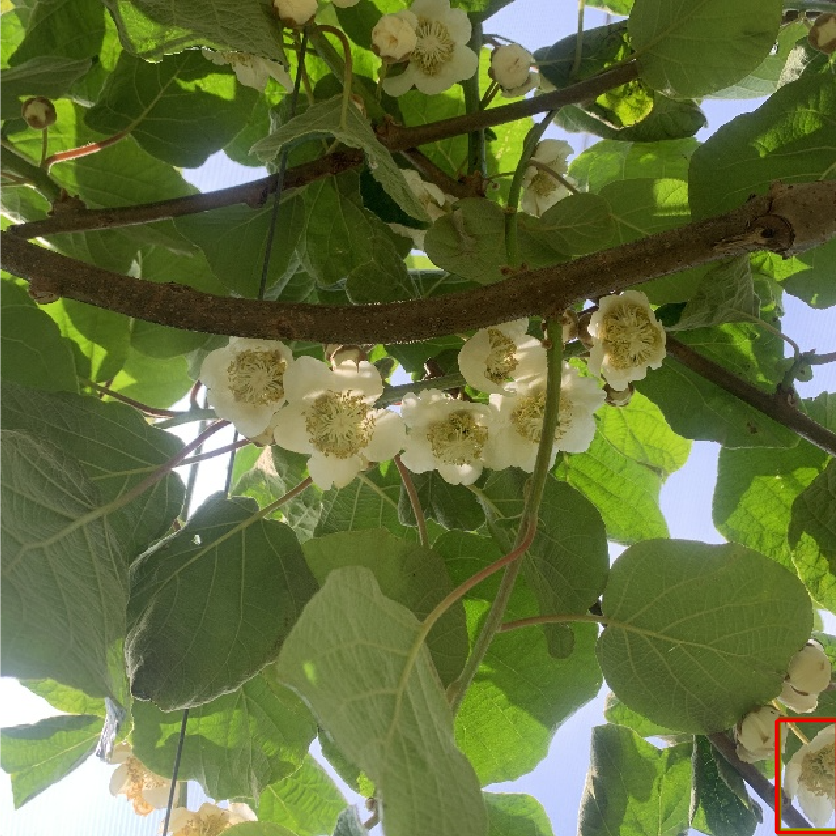

Supplement: Supplementary file 1 — Supplementary Information 1. [file 41598_2024_73035_MOESM1_ESM.zip › images/yolov8_female_fp1.png]

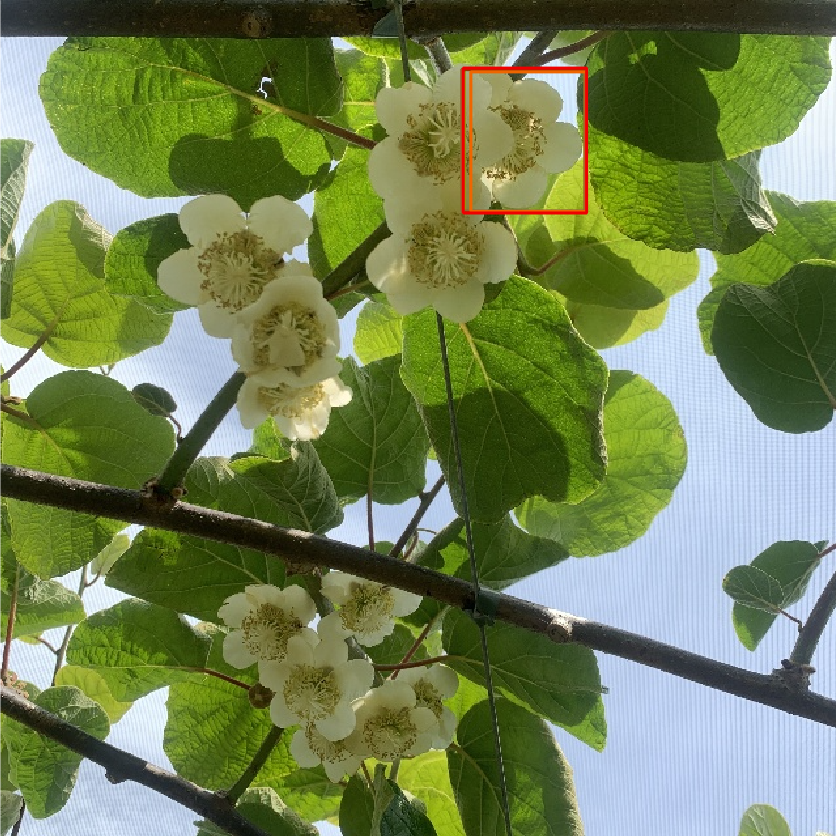

Supplement: Supplementary file 1 — Supplementary Information 1. [file 41598_2024_73035_MOESM1_ESM.zip › images/yolov8_female_fp2.png]

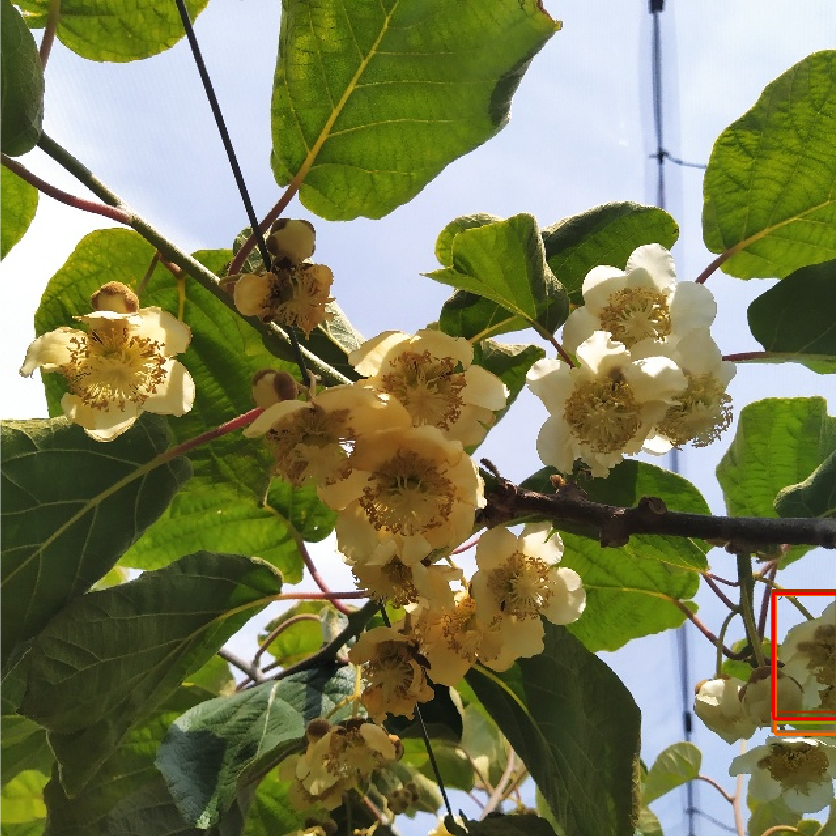

Supplement: Supplementary file 1 — Supplementary Information 1. [file 41598_2024_73035_MOESM1_ESM.zip › images/yolov8_female_fp3.png]
